# Shared cloud interactions unveil a candidate binary-system supernova pair with no known analogue

**DOI:** 10.1038/s41467-026-74978-x

**Published:** 2026-07-21

**Authors:** Miltiadis Michailidis, Marianne Lemoine-Goumard, Reinhold Willcox, Stefano Gabici, Niccolò Di Lalla, Nicola Omodei

**Affiliations:** 1 W. W. Hansen Experimental Physics Laboratory, Stanford, CA USA; 2 Kavli Institute for Particle Astrophysics and Cosmology, Stanford, CA USA; 3 Department of Physics and SLAC National Accelerator Laboratory, Stanford University, Stanford, CA USA; 4 Université Bordeaux, CNRS, LP2i Bordeaux, Gradignan, France; 5 Institute of Astronomy, KU Leuven, Leuven, Belgium; 6 Université Paris Cité, CNRS, Astroparticule et Cosmologie, Paris, France

**Keywords:** High-energy astrophysics, Particle astrophysics

## Abstract

IC 443 is one of the most extensively studied supernova remnants in the Galaxy, yet the surrounding region remains shrouded in mystery. Here we show that 16 years of *Fermi*-LAT observations uncover extended gigaelectronvolt gamma-ray emission from G189.6+3.3, a source long hidden in the shadow of the much brighter IC 443. The gamma-ray morphology is consistent with the X-ray shell detected by eROSITA, indicating an origin of the emission in the remnant. Spectral and spatial analyses identify distinct hadronic and leptonic gamma-ray components associated with regions containing and lacking molecular gas, respectively, revealing a clear spatial segregation of these emission mechanisms. In the northern shell boundary, hadronic gamma rays coincide with an H*α* filament linked to the S249 ionized hydrogen cloud. Morphological studies, crushed-cloud modelling and ultraviolet observations of this region support gamma-ray production through proton re-acceleration in compressed post-shock gas. The shared-cloud interaction positions G189.6+3.3 at the same distance as IC 443, with a linear separation between the two remnants and a time delay in their explosion events, bolstering the hypothesis that these two remnants are part of a binary system, both resulting from separate supernova events.

## Abstract (web summary)

Most stars are found in binaries, but no surviving pair of supernova remnants from the same stellar system has been found. Here, the authors show that progenitors of supernova remnants IC 443 and G189.6+3.3 may be a binary system.

## Introduction

The vast majority (if not all) of Galactic supernova remnants (SNRs), about 300 known to date^1,2^, are detected in the radio continuum band. This reflects the long lifetime of accelerated gigaelectronvolt (GeV) electrons emitting radio synchrotron radiation, which typically exceeds the 10^5^ − 10^6^ yr timescale over which an SNR remains observable before it dissipates in its surrounding interstellar medium (ISM). In fact, the thorough screening of the South African Radio Astronomy Observatory (SARAO) MeerKAT Galactic Plane Survey led to the publication of a new SNR candidate catalog that includes 237 new entries that, if confirmed, would nearly double the number of known Galactic SNRs^3^. Follow-up studies at X-ray energies have led to the detection of X-ray counterparts for approximately 30 − 40% of the total number of Galactic SNRs known to date, encompassing both thermal and non-thermal X-ray emission. Meanwhile, the new extended ROentgen Survey Imaging Telescope Array (eROSITA) discoveries have already started closing the gap between the total number of Galactic SNRs and the number of Galactic SNRs detected in X-rays (e.g.,^4–6^). This progress indicates that the previously observed discrepancy was, at least in part, driven by incomplete sky coverage and insufficient sensitivity in earlier X-ray surveys. By contrast, only about 10% of the Galactic SNRs have been detected at very high energies, that is, in gamma-rays.

Although most shell-type SNRs exhibit leptonic-induced gamma-ray emission, the discovery of a SNR in the gamma-ray domain may be interpreted as an indication of a strong hadronic component. This is because relativistic hadrons radiate predominantly at gamma-ray energies, above the kinematic threshold for *π*^0^ production. Further support for the latter hypothesis can be found in the spatial coincidence of gamma-ray emitting remnants with HII (ionized hydrogen) regions. Such findings contribute to increasing the number of objects that are available for studying the cosmic ray acceleration in the Milky Way (MW). Furthermore, these findings are crucial for expanding the number of hadronic SNR targets for next-generation neutrino telescopes, such as the Cubic Kilometer Neutrino Telescope (KM3NeT) and the IceCube-Generation 2 (IceCube-Gen2) observatory. Through dedicated individual analyses or stacked studies, these facilities will enable increasingly stringent constraints on the high-energy neutrino flux and may ultimately permit neutrino detections from this source class.

Although radio observations remain the preferred and most widely established channel for SNR identification, both because synchrotron radio emission can remain observable over long evolutionary timescales and because of the high sensitivity of modern radio surveys to extended non-thermal structures, several extended sources discovered in X-rays and/or gamma rays have been classified as Galactic SNR candidates based purely on morphological criteria, namely a shell-like appearance at high energies (e.g., refs. ^7,8^). A classic example of this class of objects is G189.6+3.3; a source initially identified as a Galactic SNR candidate based on the detection of a shell-type morphology in ROentgen SATellite (ROSAT) X-ray data^9^. The X-ray emission was found to be purely thermal, originating from shocked hot gas. Since then, no clear radio continuum counterpart has been found. Indications of an incomplete shell, primarily in the form of a strong arc, are visible across several radio surveys, most notably the Canadian Galactic Plane Survey, CGPS, and the GaLactic and Extragalactic All-sky MWA survey, GLEAM. The sole dedicated radio investigation on this object was reported by ref. ^10^, utilizing the Dominion Radio Astrophysical Observatory Synthesis Telescope (DRAO). The results show the detection of non-thermal radio synchrotron emission from the distorted incomplete shell, providing strong evidence of the object’s SNR nature. Despite this, the object continued to be classified as an SNR candidate owing to the absence of a well-defined shell-like morphology in radio continuum data.

Parts of the object were subsequently observed with the Suzaku X-ray Observatory, revealing evidence for recombining plasma^11^. Nevertheless, a complete X-ray view and a detailed spectral analysis only became available with eROSITA observations^12^, confirming earlier indications of its SNR nature.

At higher energies, above 10 GeV, ref. ^13^ reported extended GeV emission exceeding the size of IC 443 using six years of *Fermi* Large Area Telescope (Fermi-LAT;^14^) data. This source, designated Fermi Galactic Extended Source (FGES) J0619.6+2229, does not encompass the full remnant extent but appears as two distinct bright regions. It remains unclear whether this faint GeV emission originates from cosmic rays accelerated by IC 443 and diffusing into the surrounding region, or whether it is associated with a different object, speculatively including the nearby SNR candidate G189.6+3.3. Using a longer LAT data set (14 years,  > 10 GeV), ref. ^15^ subsequently updated this source to 2FGES J0618.3+2227, without reporting any association with Galactic SNRs.

**Fig. 1:**
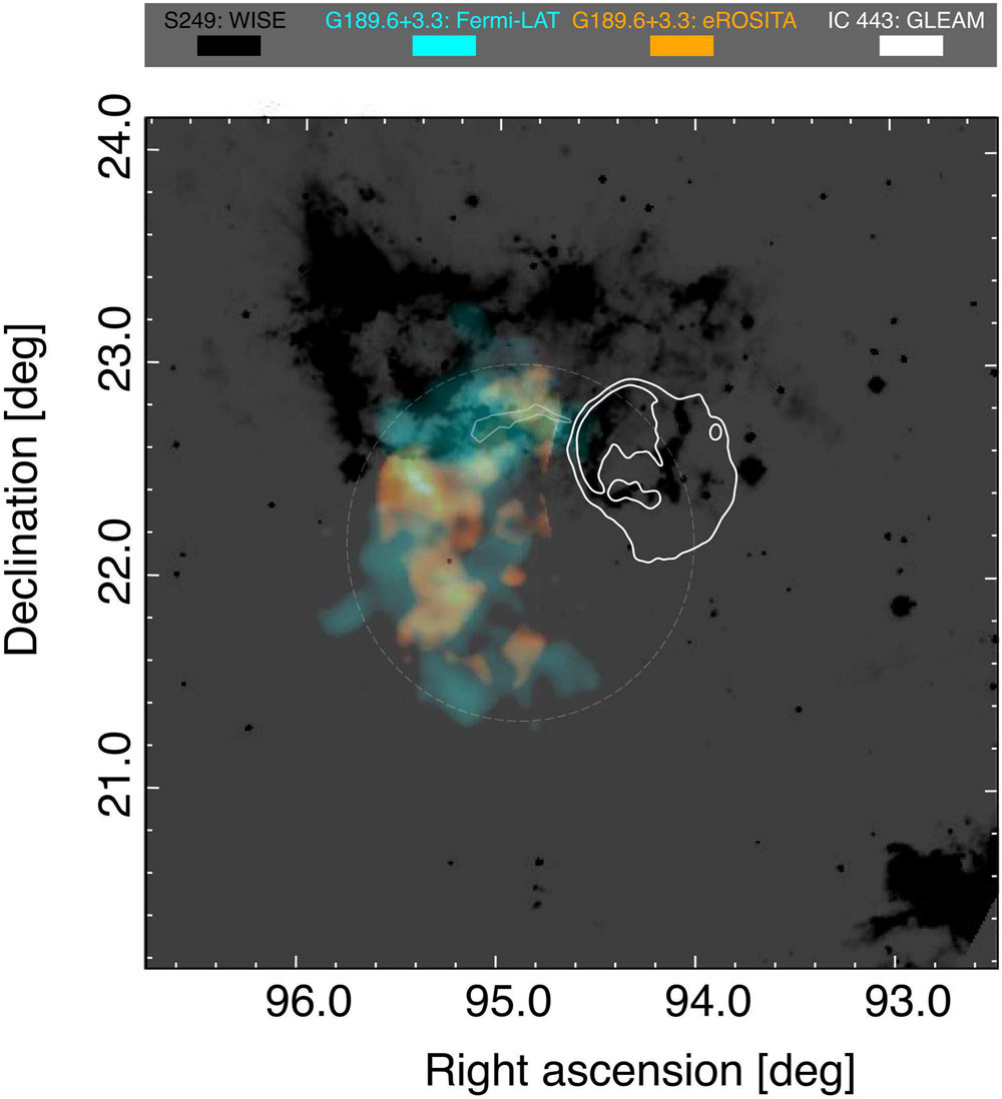
Composite map of the IC 443 complex region. Spatial comparison of X-ray, gamma-ray, and infrared (IR) emission in the vicinity of G189.6+3.3. Orange shows the eRASS1 exposure-corrected X-ray intensity map in the 0.2-5.0 keV band, identical to the right panel of Fig. 2. Cyan shows the *Fermi*-LAT TS map above 5 GeV, with gamma-ray emission from IC 443 included in the model, identical to the middle panel of Fig. 3. Black traces the S249 HII region as seen in WISE 12 micron all-sky survey data^98^. The same large-scale structure, composed of dense material, is independently detected in both CO Galactic Plane Survey data at 115 GHz^42^ and MWISP DR1 data^43^ integrated over the –10 to 10 km/s velocity range. In this interval, CO structures, in excellent alignment with the dust structures shown in the map, spatially associated with IC 443 and with the northern boundary of G189.6+3.3 are both clearly detected. Toward the Galactic anticenter, such small positive and negative velocities are compatible with material at distances of approximately 1.5–2.0 kpc, consistent with the distance of the SNRs. White contours outline the radio continuum extent of the IC 443 SNR as detected in the GLEAM radio survey. Semi-transparent overlays (dashed light-gray circle and light-gray filament) indicate the approximate spatial extent of the G189.6+3.3 SNR and the position of the H*α* filament, respectively. These overlays are included to highlight the relative extent of G189.6+3.3 and regions of overlap and potential interaction while preserving the visibility of the underlying multi-wavelength emission. The extent of G189.6+3.3 shown here is based on currently available radio and X-ray data and may represent a lower limit, given evidence from eROSITA for complete spatial overlap between G189.6+3.3 and IC 443^12^. The H*α* filament spatially coincides with the region where the hadronic *Fermi*-LAT emission overlaps with dense material traced by WISE and CO, delineating the interaction region between the G189.6+3.3 SNR and the S249 HII region.

Most studies of the IC 443 complex focus on the IC 443 SNR and its interaction with the S249 HII region, often neglecting the presence of the nearby SNR G189.6+3.3 (see Fig. 1 for the location of G189.6+3.3 relative to IC 443 and S249). Overall, G189.6+3.3 has not been extensively studied, largely because of the high complexity of the surrounding region and its faint appearance. Existing studies primarily focus on its X-ray spatial and spectral characterization. Early ROSAT observations revealed soft X-ray emission with a plasma temperature of 0.14 keV^9^, initially suggesting an old SNR with an age of about 10^5^ yr, a size of approximately 1. 5^∘^, and a distance of 1.5 kpc. The detection of recombining plasma from a portion of the remnant by Suzaku^11^ points towards a rather middle-aged remnant, although such features may also arise in older remnants depending on the ambient density. The latest X-ray study of the object using eROSITA concludes that G189.6+3.3 completely overlaps with the IC 443 SNR, based on the detection of a ubiquitous 0.7 keV plasma across both remnant’s area^12^. That study also examined the possible progenitor origin of the two remnants and explored potential pulsar associations, although no firm conclusions were reached for G189.6+3.3.

Complementary optical and HI (neutral atomic hydrogen) observations have further strengthened the SNR interpretation of G189.6+3.3. Reference ^16^ reported the detection of both filamentary and diffuse H*α* structures from both remnants. The obtained [SII]/H*α* ratio serves as an additional, and perhaps the strongest, confirmation of the object’s SNR nature. Shell-like HI structures found in the latter study could potentially be associated with the G189.6+3.3 SNR. The G189.6+3.3 SNR could be located at the same distance as IC 443 based on their HI analysis. Establishing a physical connection between IC 443 and G189.6+3.3 is particularly interesting in light of the expectation that a substantial fraction of stars form in binary systems. Despite this, no convincing case of two overlapping SNRs linked to a common binary progenitor has yet emerged. Motivated by the growing multiwavelength evidence supporting the SNR nature of G189.6+3.3, a detailed investigation of the IC 443 complex is warranted.

Here, we report the detection of gamma-ray emission from the SNR G189.6+3.3 using 16 years of *Fermi*-LAT observations. We investigate the relationship between G189.6+3.3, IC 443, and the S249 HII region by combining gamma-ray data with available X-ray, optical, and radio observations. We characterize the high-energy properties of G189.6+3.3, examine its interaction with the surrounding environment, and assess its physical connection to IC 443. Our results provide new insight into the nature of the IC 443 complex and the evolutionary history of these neighboring remnants.

## Results

**Fig. 2:**
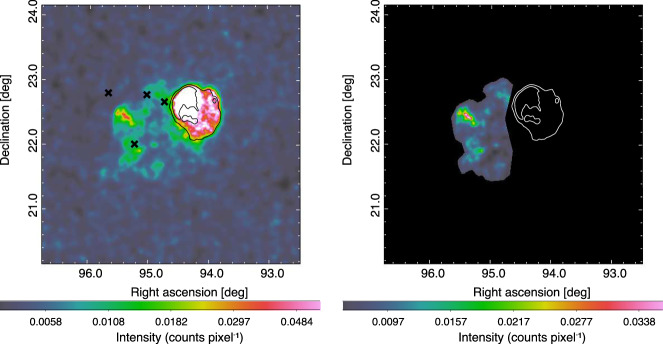
eROSITA view of G189.6+3.3. eRASS1 exposure corrected intensity sky maps in the 0.2-5.0 keV energy band, in units of counts/pixel with a pixel size of 10*″*. Point sources are filtered out, and the image is convolved with a Gaussian of *σ* = 15 pixels to enhance the visibility of the diffuse X-ray emission originating from the G189.6+3.3 SNR. The black contours (white on the right panel) illustrate the radio continuum extent of IC 443 SNR as detected in GLEAM radio survey data. Left panel: G189.6+3.3 is located just east of IC 443 SNR, and is likely to partially or completely overlap it. The positions of the three 4FGL *Fermi* sources falling within the remnant’s extension, as well as the nearby 4FGL point source lying just outside the remnant’s extension, are highlighted as black crosses. Right panel: A mask has been applied to the X-ray emission from IC 443. The map is utilized as a spatial template for the gamma-ray morphological analysis in Methods, subsection ‘Morphological analysis’, following the adjustment of pixel values to zero outside the G189.6+3.3 extension.

**Fig. 3:**
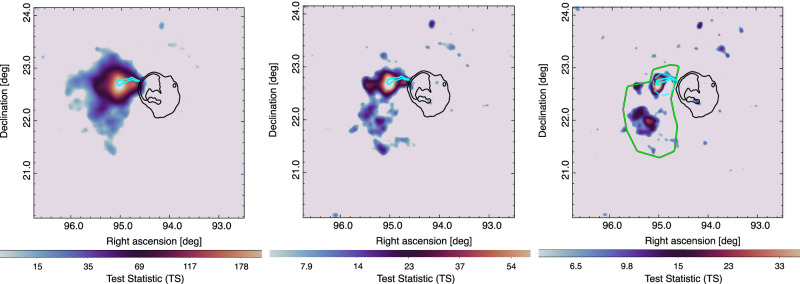
Energy-dependent gamma-ray morphology. *Fermi*-LAT TS maps with 4FGL J0617.2+2234e and 4FGL J0616.5+2235 (4FGL-DR4 sources reproducing gamma-ray emission from IC 443) included in the model. Left panel: *Fermi*-LAT TS map  > 1 GeV. Middle panel: *Fermi*-LAT TS map  > 5 GeV. Right panel: *Fermi*-LAT TS map  > 10 GeV. For display purposes, low-TS background fluctuations are suppressed in each panel using band-dependent minimum TS cuts, reflecting both the decreasing photon statistics and the improving PSF at higher energies. The adopted thresholds were chosen to remain representative of the actual extension of the emission in each energy band and to reduce low-significance residual structures likely associated with statistical fluctuations, always retaining a hard cut below approximately 2.5*σ*. Specifically, minimum cuts of TS = 7, 5.5, and 4.5 were applied to the  > 1 GeV,  > 5 GeV, and  > 10 GeV maps, respectively. The maps are intended for qualitative comparison only and do not affect any quantitative results presented in this work. The black contours illustrate the radio continuum extent of IC 443 SNR as detected in GLEAM radio survey data. The cyan contours represent the position of the H*α* filament, which is used as a spatial template to represent the gamma-ray emission from the northern part of the remnant. The dashed cyan circle indicates the one-sigma (*σ* = 0.19) extent of the best-fit Gaussian spatial model (the second component of best-fit model 13 in Table 1) and is shown for visualization purposes only; it does not correspond to a sharp boundary of the emission. The green contour outlines the polygonal region within which X-ray emission from G189.6+3.3 is traced using eROSITA (the first component of best-fit model 13 in Table 1).

This work reports a firm detection of GeV gamma-ray emission from the G189.6+3.3 SNR by using 16 yr of *Fermi*-LAT data. To this end, we demonstrate that the scenario of an extended source better describes the origin of the gamma-ray emission compared to four unidentified distinct point sources currently included in the 4FGL-DR4 catalog^17,18^ (LAT 14-year incremental point source catalog), as also illustrated by the comparison shown in Supplementary Fig. 1. These sources, namely 4FGL J0618.9+2240c, 4FGL J0620.1+2246, J0620.9+2201, and 4FGL J0622.7+2248, were previously thought to reproduce the gamma-ray emission from this sky region (see Fig. 2 for their positions relative to the system). We provide a thorough morphological analysis of both the extended GeV emission and the diffuse X-ray emission detected with eROSITA from the remnant’s location, which demonstrates the spatial coincidence of the gamma-ray enhancement with the well defined purely thermal X-ray emitting shell, as illustrated in Figs. 1 and 3. Nevertheless, the morphological analysis, together with the observed variation in gamma-ray intensity across the remnant, indicates that the emission cannot be adequately described by a single shell-like component alone and instead requires the inclusion of a secondary localized component associated with the northern boundary of the SNR. To this end, we subdivided the remnant emission into two distinct but partially overlapping regions, hereafter referred to as G189N and G189S (see Methods, subsection ‘LAT data’), as illustrated by the spatial templates shown in Fig. 3, right panel. These regions are defined to isolate physically different emission components rather than to represent strictly disjoint spatial areas. This two-component model comprises a polygonal region derived from the eROSITA X-ray image, primarily tracing the southern extension of the X-ray shell. The second component is a two-dimensional Gaussian to the north, accounting for gamma-ray emission associated with proton (re-)acceleration within an H*α* filament of confirmed SNR origin. Together, these two components provide an optimal description of the spatial extension of the gamma-ray emission.

**Fig. 4:**
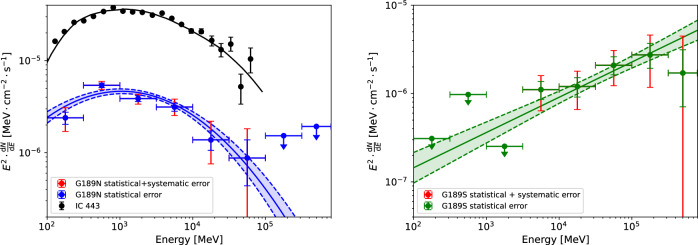
Fermi-LAT spectral energy distributions. *Fermi*-LAT SED in the 100 MeV to 800 GeV energy range from different source components of the G189.6+3.3 SNR. Left panel: G189N SED obtained using the 2D Gaussian spatial template (the second component of best-fit model 13 in Table 1). IC 443 *Fermi*-LAT SED is shown in black^26^. The best fit gamma-ray spectrum from pion decay is indicated by a solid black line. The corresponding data points are scaled downwards by a factor of 5 × 10^−2^ to facilitate comparison with G189N. Right panel: G189S SED obtained using the X-ray eROSITA polygon as spatial template (the first component of best-fit model 13 in Table 1). For both SEDs, error bars correspond to the 1*σ* statistical uncertainties, while the red points show the statistical and systematic uncertainties added in quadrature. Upper limits correspond to energy bins with TS < 4 and are shown at the 95% confidence level; therefore, they do not constitute statistically significant constraints. In this low-significance regime, small excursions of the global best-fit spectral model relative to individual upper limits are expected, particularly in the presence of partially overlapping spatial components, and do not indicate tension with the fit.

**Fig. 5:**
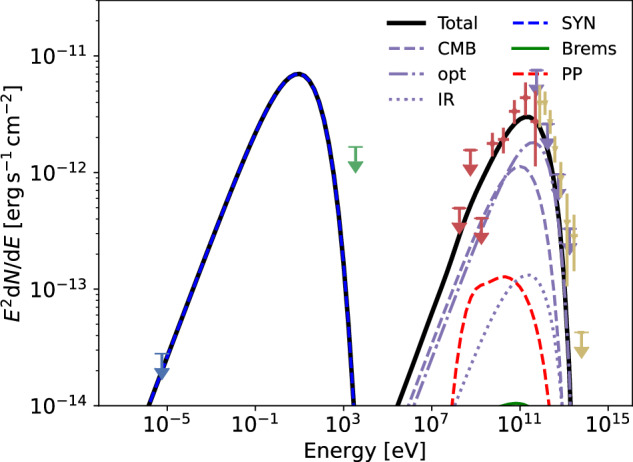
SED modeling of G189S. Multiwavelength SED modeling of the southern region of G189.6+3.3 SNR (G189S). For all data sets, only statistical errors are presented. The blue dashed line represents the synchrotron emission. The black solid and red dashed lines display the total and pion decay emission. The observed gamma-ray data are predominantly described by IC scattering. The latter component is best represented by the combination of the purple dashed, the purple dash-dotted, and the purple dotted lines which account for IC scattering off the cosmic microwave background (CMB), optical, and infrared (IR) photon fields, respectively. The Bremsstrahlung emission (green solid line) is suppressed by the low density of the medium. TeV data points (gold;^68^) and upper limits (purple;^67^) from the LHAASO C1 component and HAWC, respectively, are overlaid for reference only and were not used to constrain the model. Differences between these measurements likely reflect the use of different spatial templates.

**Fig. 6:**
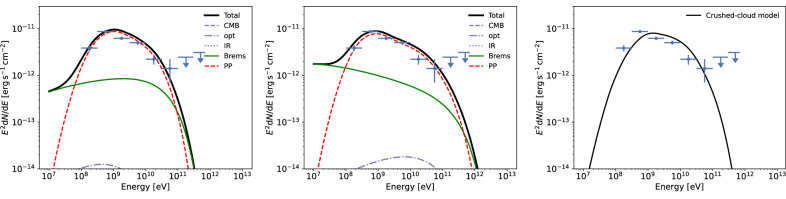
SED modeling of G189N. Multiwavelength SED modeling of the northern region of G189.6+3.3 SNR (G189N; same convention as Fig. 5). Left panel: Injection spectrum fixed at 2.0 and energy cut-off at 100 GeV; Middle panel: Softer injection spectrum of 2.3 with an energy cut-off at 600 GeV. Right panel: Crushed-cloud modeling scenario.

The favored scenario explains the nature of the observed gamma-ray emission on the basis of the remnant’s interaction with the S249 HII region to the north. In particular, the significantly increased gamma-ray emission from the region of the remnant overlapping the S249 HII region, compared to the rest of its area, as illustrated in Fig. 3, which also renders a single spatial template insufficient, initiated a deeper spectral investigation. Our results support a scenario where hadronically induced gamma-ray emission dominates the northern part of the remnant, whereas accelerated leptons are responsible for the gamma-ray emission detected from the southern parts of the remnant that are free of molecular gas. More explicitly, the GeV spectral shape measured by *Fermi*-LAT in the northern region—characterized by spectral curvature, a flux decrease at a few hundred MeV, and the absence of a characteristic inverse-Compton (IC) peak at teraelectronvolt (TeV) energies (Fig. 4, left)—supports a hadronic interpretation. In contrast, the southern component exhibits a pronounced IC peak (Fig. 4, right). These indications based on spectral shape were further corroborated by detailed multiwavelength spectral energy distribution (SED) modeling, confirming the nature of the gamma-ray emission from G189.6+3.3 (see Methods, subsection ‘Multiwavelength SED modeling’, and Figs. 5 and 6). Deep observations with the upcoming Cherenkov Telescope Array Observatory (CTAO) would give clear insights into the maximum particle energy which remain unconstrained for both components. Nevertheless, to our knowledge, no other SNR has been reported to exhibit such a clear spatial separation of distinct, dominant gamma-ray-producing particle populations across its extent.

Consequently, G189.6+3.3 presents an unusual opportunity for neutrino studies within this source class, as it allows the hadronic and leptonic contributions to be disentangled. To date, neutrino studies from SNRs have yielded null results (e.g.,^19,20^). It is important to acknowledge that leptonic contributions can dilute the signal, providing strong gamma-ray signatures but lacking neutrino contributions. Identifying relatively nearby SNRs ( ≲ 2 kpc) with dominant hadronic gamma-ray components, such as G189.6+3.3, is therefore important for neutrino astronomy. Although G189.6+3.3 is relatively faint and brighter SNRs are likely to be prioritized in neutrino searches, sources of this kind define the population capable of producing neutrinos via pion decays and are essential for population studies and for constraining expected neutrino fluxes with future neutrino telescopes. This connection follows from the common origin of hadronic gamma rays and neutrinos in pion production.

Moreover, the spatial coincidence of the gamma-ray emission from the northern part of the remnant with a conspicuous H*α* filament (refer to Fig. 3)—characterized by optical forbidden-line emission^16^ and also traced in the ultraviolet (UV; refer to Supplementary Fig. 2)—provides strong morphological support for a hadronic-dominated component and for proton re-acceleration through the presence of radiative shocks. Taken together with the crushed-cloud modeling of the northern hadronic component presented in Methods, subsection ‘Proton re-acceleration’, and illustrated in the right panel of Fig. 6, this allows a confident interpretation of the gamma-ray emission from this region as arising from proton re-acceleration induced by adiabatic contraction within the filaments. This provides direct evidence of the interaction between the remnant and the S249 HII region. Such a discovery places G189.6+3.3 at the same distance with the IC 443 SNR.

**Fig. 7:**
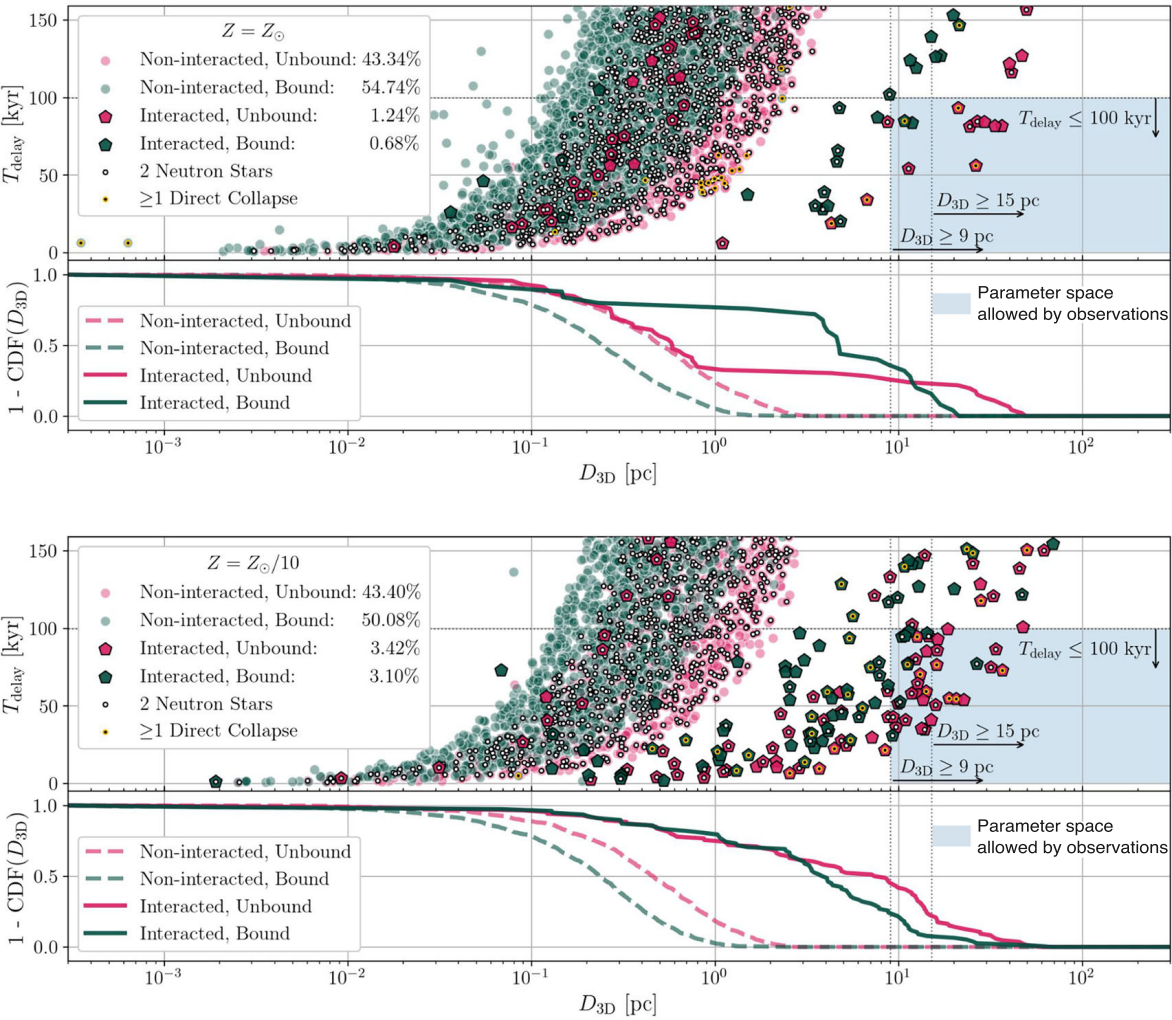
Binary-supernova simulation predictions. The time delay vs separation of SNRs predicted by the rapid population synthesis of one million massive binaries, at two different metallicities. If a binary is disrupted during the first supernova (Unbound, magenta systems), the post-explosion velocity of the companion is used to calculate the travel distance, otherwise (Bound, green systems) the post-explosion center-of-mass velocity is used. Binaries that were born too wide to interact (Non-interacted, half-shaded circles and dashed lines) have low orbital velocities and are unable to travel significant distances whether bound or unbound. By contrast, closer systems which experienced mass transfer prior to the first supernova (Interacted, solid pentagons and solid lines) have higher orbital velocities and travel further for the same delay time. We further identify binaries that formed two neutron stars (black circle with white dot) and those which experienced at least one direct collapse (yellow circle and black dot). The light-blue box highlights the region of parameter space consistent with the observational constraints derived for the IC 443 and G189.6+3.3 system, including the projected separation between the remnants and the difference in their inferred ages.

This intriguing discovery necessitated a comprehensive analysis of the intrinsic properties of both remnants to substantiate this finding. Consequently, distance constraints for the G189.6+3.3 SNR were established utilizing X-ray absorption column density values derived from the newly recorded eROSITA data, alongside the latest optical extinction datasets based on the Global Astrometric Interferometer for Astrophysics (GAIA) and the Two Micron All Sky Survey (2MASS), as provided by ref. ^21^ (see Methods, subsection ‘Distance estimates’). A common distance of approximately 1.8 kpc was determined for both remnants. Furthermore, the uncertainty range concerning the age difference between the two remnants was meticulously examined by considering reports employing various methodologies in the literature, as well as estimates conducted in this study (see Methods, subsection ‘Age estimates’). A delay of 20-100 kyr in the explosion of the progenitors of the two remnants was identified. The simulation of one million binaries reported in this study shows that interacting systems can readily produce SNR pairs with separations of  ≲ 40 pc and delay times of  ≲ 100 kyr (see Fig. 7). Integrating this result with the distance and age constraints derived for our system provides strong support for the hypothesis of a binary system undergoing supernova explosions at different times, potentially providing further insight into supernova explosion mechanisms and the evolution of massive binary systems.

Our results advance our understanding of the IC 443 complex region and provide compelling evidence for a binary-supernova system in which both components experienced supernova events; to our knowledge, no comparable example has previously been reported. Consequently, IC 443, recognized as one of the most luminous sources in the GeV gamma-ray sky, should no longer be regarded as an isolated object (as done so far) but rather as a system in which contributions from SNR G189.6+3.3 are undoubtedly present across its area. This system could offer a valuable avenue for investigating the energetics and evolution of massive binary systems. To this end, more comprehensive multiwavelength observations of the IC 443 complex region are strongly motivated to further elucidate the metallicity of the environment and the potential association of compact objects with both remnants. Future progress will benefit from deeper X-ray and optical spectroscopy of the interaction region between both SNRs and S249 to constrain shock conditions and abundances, improved molecular gas mapping to refine target densities, and enhanced MeV-GeV gamma-ray sensitivity to better resolve the low-energy hadronic spectrum. Finally, the clear separation of the hadronic and leptonic components from this SNR highlights the discovery of a prime target for future high-energy neutrino studies from this source class.

## Discussion

### Nature of the gamma-ray emission

Typical young age SNRs that are characterized by leptonically induced gamma-ray emission exhibit a characteristic IC peak at TeV energies (e.g., RX J1713.7-3946^22,23^ and RX J0852.0-4622^24^). In contrast, middle-aged SNRs interacting with molecular gas and producing gamma-ray emission through *π*^0^ decay typically exhibit spectral curvatures at the energy range of hundreds of MeV’s to several GeV’s ^25^ (e.g., IC 443, W44^26^, W51C^27^, HB 21^28^, G39.2-0.3^29^, and several remnants inspected in ref. ^30^). As such, the gamma-ray spectrum can be used to draw useful conclusions about the origin of gamma-ray emission.

To this end, we inspected the gamma-ray emission from two distinct prominent regions of G189.6+3.3 (G189N and G189S) that exhibit strong variations in gamma-ray intensity and TS values (refer to Methods, subsection ‘LAT data’, for the definition). For the G189N component, the spectral analysis yielded significant spectral curvature which is best described by a LogParabola model (see left panel in Fig. 4). In addition, there is an apparent decrease in the flux levels at lower energies, which could be indicative of a strong hadronic component. The clear spectral curvature at GeV energies obtained, coupled with the flux decrease at a few hundreds of MeV’s and the spatial coincidence of the gamma-ray emission with enhanced molecular gas regions and with the conspicuous H*α* filament, supports a hadronic gamma-ray scenario. It is worth noting that a powerlaw of *Γ* > 2.2 would be necessary to explain the signal decrease  > 1 GeV. Contrary to this, the obtained gamma-ray spectrum for the southeastern region of the remnant is steeply rising (*Γ* = 1.6) to several hundreds of GeV energies, which is consistent with an IC-dominated spectral component peaking at TeV energies (Fig. 4; right panel). Such a spectral shape agrees with typical leptonically induced gamma-ray emitting SNRs, as mentioned above. Overall, by comparing the spectral shapes of the two components studied in this paper with classic examples in the literature (mentioned above) but also with the gamma-ray spectrum obtained from the neighboring IC 443 (see Fig. 4), we argue that gamma-ray emission is dominated by a hadronic component in the northern part of the remnant whereas accelerated leptons emitting gamma-rays via IC dominate in the remnant’s southern regions.

Puppis A is often cited as an exemplar of a remnant exhibiting predominantly hadronic gamma-ray emission, largely attributed to its strong spatial overlap with dense interstellar material located at the same distance. However, there is no compelling evidence for any region within Puppis A where leptonic gamma-ray emission dominates the observed signal^31,32^. Although rare, a few well-known TeV SNRs (e.g., RX J1713.7-3946 and Vela Jr.) have been reported to exhibit mixed leptonic and hadronic gamma-ray emission (e.g.,^33,34^), in which both processes contribute to the observed signal, often within the same spatial regions. In most such systems, however, regions associated with dense material still retain a substantial co-spatial leptonic component, preventing a clean spatial separation of the two emission mechanisms. SN 1006 provides another relevant comparison (and perhaps the closest analog to G189.6+3.3 in terms of the gamma-ray emission mechanisms operating in its distinct limbs), as it clearly demonstrates leptonic gamma-ray emission from its northeastern limb. Reference ^35^ suggests that, owing to an enhanced ambient medium, hadronic gamma-ray emission may contribute more significantly in the southwestern region than in the northeastern limb. Nevertheless, the gamma-ray emission from this region still appears to involve significant contributions from both leptonic and hadronic processes, and the limited statistical significance prevents a firm determination of the dominant emission mechanism there. In this respect, G189.6+3.3 represents an SNR exhibiting a clearly spatially segregated gamma-ray emission morphology, modeled by two distinct spatial components dominated by different particle acceleration mechanisms, a configuration not previously reported in an SNR. In this system, hadronically induced gamma-ray emission is confined to regions interacting with molecular gas, where the dense material provides an efficient target for relativistic protons and the leptonic component appears comparatively faint or suppressed. In contrast, regions elsewhere in the remnant that are not spatially coincident with dense material show no evidence for a hadronic component and are instead dominated by leptonic gamma-ray emission. This interpretation is further supported by the results of the multiwavelength SED modeling, as discussed in detail in Methods, subsection ‘Multiwavelength SED modeling’. Thus, G189.6+3.3 is identified as a prime target to set constraints on the expected neutrino flux from SNRs and for future SNR neutrino searches.

### Evidence for proton re-acceleration: a prominent optical and UV filament

Reference ^16^ reported a high resolution optical H*α* detection of filamentary structures from IC 443 and G189.6+3.3 which are also apparent in the digitized optical sky survey (DSS) sky map data^36^. In the latter scenario, that is G189.6+3.3, they find a prominent filament in the northern half of the remnant (regions 7 and 8 in the related study). This feature is also apparent in the second digitized optical sky survey (DSS2). Notably, this filament has been recognized and investigated for several decades. Reference ^37^ demonstrated that its line intensities differ significantly from those typically observed in HII regions and are consistent with shock-heated interstellar gas observed in SNRs. These results have been confirmed in the study by ref. ^16^. Reference ^37^ further speculated that the filaments might belong to an older, unrecognized SNR distinct from IC 443. Other than this filament of an arc structure, there are no other significant H*α* structures found from the remnant, with the exception of the presence of some smaller and fainter filaments extending beyond the IC 443 southeastern shell, which are indicative of a potential interaction between the two remnants^38^. As a result, the lack of a complete H*α* image prevents testing an H*α* template as a spatial model for the gamma-ray emission.

**Fig. 8:**
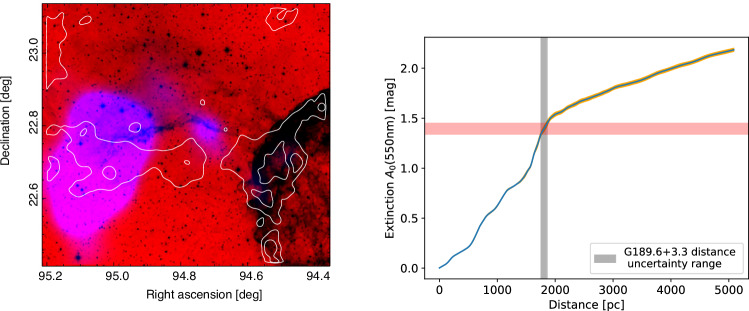
Optical morphology and distance constraints. Left panel: Composite image depicting the northern region of the G189.6+3.3 SNR. Red: DSS2 Red sky map^36^. Enhanced emission regions appear in black. Blue: *Fermi*-LAT TS map  > 10 GeV. White contours indicate the regions of higher density within the S249 HII region, as inferred from WISE 12 micron all-sky survey data^98^. Right panel: One-dimensional cumulative extinction graph which depicts the increasing extinction along the line of sight toward the supernova remnant G189.6+3.3, up to a distance of approximately 5 kpc. This graph was generated using the EXPLORE G-Tomo tool, which computes one-dimensional extinction based on the updated data sets from^21^. The uncertainty in the extinction estimate at larger distances is displayed in orange. The red shaded region represents the uncertainty in the optical extinction estimate, derived from the uncertainty in the X-ray absorption column density. The gray-shaded area corresponds to the distance uncertainty of the G189.6+3.3 supernova remnant.

Even so, the spatial coincidence between the G189N gamma-ray component and the H*α* filament (Figs. 3 and 8) suggests that this part of gamma-ray emission (which appears significantly enhanced compared to the G189S) could be the result of thin filaments presence. Proton re-acceleration and subsequent diffusion in the surrounding medium could be enabled by adiabatic compression within the filaments. This scenario is strongly supported by the morphological analysis results, which demonstrate that the bright gamma-ray blob at the northern part of the remnant is best described by a 2D-Gaussian template centered at the filament’s location (Fig. 3; right panel). In this context, the spatial coincidence of the prominent H*α* filament with gamma-ray emission detected  > 10 GeV from the G189N component was put into perspective. Restricting to such energies allows a significantly improved point-spread function (PSF), reaching angular resolutions of order  ≲ 0. 1^∘^ (68% containment), which allows well-localized gamma-ray structures to be resolved. As shown on the left panel of Fig. 8, there is an apparent spatial coincidence of the gamma-ray blob  > 10 GeV detected to the northern part of the remnant with the H*α* filament detection reported in ref. ^16^. Such is evident of the interaction of the remnant with S249 HII region, especially when there is absence of prominent filamentary structures from the southern parts of the remnant.

We note that a standard diffusive shock acceleration (DSA;^39,40^) scenario, in which shock-accelerated protons escape upstream and subsequently interact with nearby dense gas, cannot be ruled out based on the gamma-ray spectrum alone. Indeed, the spectral shape by itself does not uniquely distinguish between different hadronic mechanisms (Fig. 6). However, the observed morphology and the close spatial correspondence described above provides an important additional diagnostic. In particular, it lends support to a radiative-shock scenario and disfavors an interpretation dominated by runaway cosmic rays escaping ahead of the shock front, which would generally predict gamma-ray emission offset from the remnant boundary and correlated with more distant cloud structures. Instead, the gamma-ray emission appears spatially confined to the northern interaction region, supporting local hadronic emission associated with the shock-cloud interface.

Additional observational support for this interpretation comes from optical and UV observations of the northern filament. Independent multiwavelength evidence supports the presence of radiative shocks in this region. Optical spectroscopy of the filament reveals strong forbidden-line emission, including N II, S II, O I, and O III, with elevated S II to H*α* ratios in the range of approximately 0.5–1.4^16^. These values are characteristic of radiative shock conditions, where post-shock gas undergoes efficient cooling and strong compression, and they rule out a purely Balmer-dominated, non-radiative shock interpretation. In addition, the filament is detected in UV emission with the Ultra-Violet/Optical Telescope (UVOT) aboard the *Neil Gehrels* Swift Observatory. UV-emitting filaments are commonly observed in evolved SNRs in which shocks have slowed and entered the radiative regime (e.g.,^41^). Taken together, the combination of optical forbidden-line diagnostics, UV emission, and the spatial correspondence with the northern hadronic gamma-ray component—which is also coincident with dense material—provides a coherent picture in which the shock has transitioned into a radiative phase. Additional support for an interaction scenario comes from the enhancement in X-ray plasma temperature reported from the designated region B in ref. ^12^, a region partially overlapping the S249 HII cloud. In such conditions, diffusive shock acceleration is expected to be inefficient, whereas compression and re-acceleration of pre-existing Galactic cosmic rays within the cooled post-shock gas become dominant. This multiwavelength evidence therefore supports a crushed-cloud origin for the northern hadronic gamma-ray emission, rather than a scenario involving efficient particle acceleration at a fast, non-radiative shock (see Methods, subsection ‘Proton re-acceleration’).

To further substantiate this interpretation, we explored a radiative-compression (“crushed cloud”) scenario for the northern region (refer to Methods, subsection ‘Proton re-acceleration’). In this framework, proton re-acceleration within radiatively cooled, compressed post-shock gas provides a natural explanation for the observed GeV gamma-ray emission. The crushed-cloud modeling demonstrates that such proton re-acceleration is physically plausible and can simultaneously account for the gamma-ray spectrum and the associated radio synchrotron emission, without requiring efficient fresh particle acceleration at the shock front.

Nevertheless, some additional physical and observational considerations remain relevant to this interpretation, as discussed below. It is noteworthy that particle acceleration and transport in dense, partially ionized environments are subject to additional physical effects that can influence non-thermal emission. In particular, ion-neutral damping suppresses the growth of Alfvénic turbulence in radiative shocks, thereby reducing the efficiency of prolonged diffusive shock acceleration and limiting long-term particle confinement. Deviations from Bohm diffusion are also expected in dense clouds, leading to faster particle transport and shorter residence times. These effects primarily impact scenarios that rely on sustained confinement of freshly accelerated particles near the shock. In the present case, however, the dominant process is not classical multi-cycle acceleration, but rapid reprocessing and adiabatic compression of pre-existing cosmic rays in a radiative shock environment. In this scenario, as the shocked gas cools and compresses on timescales short compared to the system age, cosmic-ray momenta and energy density are promptly enhanced without requiring long-lived confinement or efficient fresh acceleration. Gamma-ray production therefore occurs locally at the shock-cloud interface during the compression phase, governed mainly by the high target density, and remains viable even in the presence of strong wave damping and non-Bohmian diffusion. This explains why the hadronic interpretation of the northern emission through reprocessing and re-acceleration is robust against these transport effects.

Additional direct observational tracers of the shock-cloud interaction would nevertheless be valuable. Unambiguous confirmation of a physical association between dense gas and the hadronically induced gamma-ray emission would require targeted molecular observations sensitive to shock-processed material. In particular, the detection of OH(hydroxyl; 1720 MHz) maser emission—a well-established signpost of SNR-molecular cloud interactions—or of other shock tracers such as SiO (silicon monoxide) emission, enhanced high-density tracers (e.g., formyl cation, HCO^+^; hydrogen cyanide, HCN; carbon monosulfide, CS), and/or spatially localized CO (carbon monoxide) line broadening coincident with the gamma-ray emission would provide direct proof of an interaction between the SNR shock and molecular material. We searched the literature and existing maser catalogs for reports of OH(1720 MHz) emission toward the IC 443/G189.6+3.3 region, but no such detections have been reported to date for G189.6+3.3. This is not unexpected, as G189.6+3.3 has long been classified only as an SNR candidate, and its presence—let alone a potential interaction with surrounding molecular material—had not been firmly established or systematically investigated. Therefore, we emphasize that the absence of reported OH maser detections does not weaken the proposed interaction scenario. A detailed characterization of the molecular shock properties would require dedicated follow-up observations and therefore falls beyond the scope of the present work. Instead, the existing multiwavelength evidence—including the spatial coincidence of the hadronic gamma-ray component with dense material traced by the Wide-field Infrared Survey Explorer (WISE) and CO (from the CO Galactic Plane Survey^42^ and the Milky Way Imaging Scroll Painting [MWISP] survey^43^), the correspondence with the S249 HII region, and the presence of an ultraviolet shock signature and optical forbidden lines at the northern boundary of the remnant—collectively provides strong and internally consistent evidence for a physical interaction.

### A binary supernova scenario

Overall, the evidence discussed above provides compelling support, if not a near-certain case, that the G189.6+3.3 SNR interacts with the adjacent S249 HII region to the north. Independent evidence already indicates that IC 443 itself is interacting with S249, based on morphological correspondence, enhanced emission along its northeastern rim, and signatures of particle acceleration in dense material associated with the HII region^37,44–46^. Collectively, these observations, together with the HI analysis reported in ref. ^16^, which identified HI structures potentially associated with G189.6+3.3 exhibiting broadly consistent systemic velocities with those of IC 443, as well as high-velocity wings within the overlap region of the two remnants that may hint at a physical interaction between the SNRs, place G189.6+3.3 and IC 443 at a common distance and prompt further investigation of their physical relationship. To strengthen the argument for compatible distance measurement of the two remnants, additional data sets were employed to provide further distance estimates. The various methodologies employed for distance measurement are comprehensively outlined in Methods, subsection ‘Distance estimates’. The results obtained strongly support a common distance of 1.8 kpc for the two SNRs. As such, the IC 443/G189.6+3.3 system occupies a currently unrepresented region of the known SNR population, comprising two overlapping remnants situated at a common distance. Consequently, they emerge as a leading candidate for a binary system that has experienced two distinct supernova events.

### Binary-system plausibility

However, it is further necessary to estimate the ages of the two remnants in order to assess the plausibility of a binary-system origin in which both components underwent supernova explosions. The time delay of the two explosions when combined with the linear separation of the explosion centers, estimated to be 9–15 pc (as explained below), should not prohibit a binary system progenitor origin. To this end, we employed three distinct methodologies to provide an age estimate of the G189.6+3.3 SNR (see Methods, subsection ‘Age estimates’). A 20 − 110 kyr age was obtained. A 30 − 100 kyr age has been proposed in the literature^9,10^. Based on literature reports, a 8 − 9 kyr age emerges as the most likely estimate for IC 443^47^.

To this end, we simulate one million binary systems using the rapid binary population synthesis code Compact Object Mergers: Population Astrophysics and Statistics (COMPAS), adopting the default settings described in ref. ^48^. COMPAS evolves binary systems through stellar evolution, mass transfer, and supernova explosions, tracking the orbital properties, systemic velocities, and disruption outcomes following each supernova. From this population, we select all systems that experience two supernovae. For each such system, we extract the time delay between the two explosions and compute the distance traveled using either the post-explosion velocity of the companion (if the system is unbound after the supernova) or otherwise the system center-of-mass. The resulting distributions are shown in Fig. 7. The figure on the top shows the results for solar metallicity (*Z*_⊙_ = 0.0142,^49^) while the figure on the bottom shows the same at *Z* = *Z*_⊙_/10, considered to be extremal values for a recently formed binary in the MW. We distinguish binaries which did or did not disrupt during the first supernova (maroon and green colors, respectively), as well as those which did or did not interact prior to the first supernova (half-shaded circles and dashed lines, and solid pentagons and lines, respectively).

We highlight in gray the region given by time delays *T*_delay_≤100 kyr and projected separations 9≤*D*_⊥_/pc≤15, corresponding to the range of values allowed for the IC 443 and G189.6+3.3 system. The upper limit on *T*_delay_ is set by the estimated ages of the two remnants. The projected separation range is obtained by converting the angular separations between the explosion centers reported in the literature (e.g.,^12,47^) into physical distances assuming a common distance of 1.5–1.8 kpc. We note that the 3D distance *D*_3D_ reported here is larger than the projected separation by a factor $\sin (i)$ (where *i* stands for inclination). At both metallicities, this region is populated only by SNR pairs from binaries that had previously interacted. The higher number of points in this region at *Z*_⊙_/10 suggests that low metallicity environments are more efficient at producing IC 443 + G189.6+3.3-like SNR pairs.

The 1-CDF curves below each plot in Fig. 7 indicate that the fraction of post-interaction binaries with separation greater than 9 pc is between 25% and 50% for bound and unbound systems, at both metallicities. These results demonstrate that the observed properties of the IC 443/G189.6+3.3 system, including the inferred separation and explosion-delay constraints, are naturally reproduced within binary stellar evolution models. The likelihood that IC 443 and G189.6+3.3 formed from a binary is nevertheless metallicity dependent and characterization of the environment composition would help to further argue for or against the binary formation scenario. Intriguingly, there is a substantial population of SNRs from non-interacted binaries that are separated by less than a few pc. These make up the vast majority of the predicted SNR pairs, which suggests that there should be a large population of SNR pairs at much closer separations, which may be interacting and/or appear single.

Additional support for a binary-system origin comes from independent indications that both remnants originated from massive progenitor stars. Low metallicity environments are conducive to the formation of massive stars due to diminished cooling rates and elevated gas temperatures compared to metal-rich regions (e.g.,^50^ and references therein). This observation aligns with the hypothesis of a massive progenitor origin for the two SNRs, as suggested by the presence of jet-like structures. For G189.6+3.3, this is illustrated by the elongated X-ray feature, oriented from the southeast to the northwest, in the left panel of Fig. 2 and further detailed in ref. ^12^. Additionally, a second jet-like structure, likely associated with IC 443, is discussed in ref. ^51^. Jet-like features are notably anticipated from progenitors with masses exceeding 30M_⊙_, such as luminous blue variable stars (LBVs)^52^. These features may also arise from strong stellar winds of the progenitor, which reshape the surrounding medium (e.g.,^53^). In both scenarios, a particularly massive progenitor, potentially surpassing 30M_⊙_, is required.

In addition, the X-ray ejecta properties of G189.6+3.3, as revealed by the eROSITA spectral analysis^12^, indicate metal-enriched plasma whose elemental abundances closely align with those proposed for LBV progenitors in ref. ^53^. While such abundance patterns, including supersolar O/Ne/Mg/Si, are consistent with a stripped-envelope core-collapse explosion naturally expected for very massive stars in close binaries, we note that they do not uniquely identify the stripping mechanism. Nevertheless, they provide further supporting evidence for this interpretation. Several studies have reported a progenitor mass exceeding 20 − 25M_⊙_ or even approximately 30M_⊙_ for IC 443^54–56^. These features suggest that both SNRs originate from massive progenitor stars, allowing a larger separation of the explosion centers, hence further supporting the binary system scenario. Furthermore, the proportion of stars expected to exist within binary (or even multiple) systems significantly increases with stellar mass, reaching 100% for the most massive stars (e.g.,^57^ and references therein). In this context, the combined constraints from progenitor mass estimates, jet-like features, X-ray ejecta properties, and population statistics collectively support a binary system origin for G189.6+3.3 and IC 443.

### Chance-alignment probability

To place the proposed association in context, we consider two complementary estimates: (i) the probability that two unrelated SNR explosions occurred unusually close together on the sky, and (ii) the probability that two unrelated extended SNR shells overlap in projection by chance. In both cases, we further require distance compatibility. The first is the physically relevant metric when assessing scenarios involving a common origin or binary progenitors. Using the surface density of cataloged Galactic SNRs, the 2024 October version of Green’s catalog contains 310 Galactic remnants^2^. Assuming conservatively that these are distributed over ∣*b*∣≤ 5^∘^ (area approximately 3600 deg^2^), the implied surface density is *Σ*_SNR_ ≲ 0.086 deg^−2^. For a characteristic angular separation *θ* = 0. 3^∘^ − − 0. 5^∘^ between G189.6+3.3 and IC 443, the expected number of unrelated remnants within an area *A* = *πθ*^2^ is *λ* ≲ *Σ*_SNR_*A* = 0.025 − 0.07, corresponding to a chance angular coincidence probability of approximately 2.5% − − 6.5%. Here, *θ* denotes the center-to-center angular separation uncertainty between the two remnants and a Poisson distribution for the positions of Galactic SNRs on the sky is assumed, such that the probability of at least one chance remnant within an area *A* is given by $P(\ge 1)=1-\exp (-{\Sigma }_{{{{\rm{SNR}}}}}A)$. Requiring distance compatibility further suppresses this probability. Our conservative distance constraints, based on absorption column density and optical extinction, place G189.6+3.3 in the range 1.7–2.3 kpc (see Methods, subsection ‘Distance estimates’). IC 443 is placed in the range 1.5–2.0 kpc based on literature estimates (see Methods, subsection ‘Distance estimates’), implying an overlap interval of 1.7–2.0 kpc (width Δ*d* ≃ 0.3 kpc). We emphasize that the distance ranges for both SNRs are not excessively broad and overlap substantially, rather than only at a narrow boundary, such that the compatibility criterion is not driven by an incidental intersection of large distance uncertainties. Treating this overlap as the distance-compatibility window and adopting a conservative effective Galactic distance span of order 10 kpc yields a fractional overlap *f*_*d*_ = Δ*d*/10 kpc = 0.03, and thus an order-of-magnitude joint probability

$${P}_{{{{\rm{joint}}}}}=P(\ge \,1)\,{f}_{d}=(0.6-2)\times 1{0}^{-3}.$$

Here, the adopted effective Galactic distance span of 10 kpc reflects the typical range from the Sun over which the majority of cataloged Galactic SNRs are observed.

We next consider the complementary question of how likely it is that two unrelated extended SNR shells overlap in projection by chance. In this case, overlap occurs when the centroid separation is smaller than the sum of the angular radii of the two remnants, *R*_IC443_ + *R*_G189_. Adopting representative angular diameter of $45{\prime}$ for IC 443 and $90{\prime}$ for G189.6+3.3 from^2^, this defines an effective overlap area

$${A}_{{{{\rm{ov}}}}}=\pi {({R}_{{{{\rm{IC443}}}}}+{R}_{{{{\rm{G189}}}}})}^{2}\simeq 3.98\,{{{\rm{deg}}}}^{2}.$$

Using the same surface density of Galactic SNRs, the expected number of unrelated overlapping remnants is *λ*_ov_ = *Σ*_SNR_*A*_ov_, corresponding to a purely angular overlap probability of order a few tens of percent. Applying the same distance-compatibility requirement as above suppresses this to a joint probability at the sub-percent level,

$${P}_{{{{\rm{joint}}}}}^{{{{\rm{ov}}}}}=9\times 1{0}^{-3},$$

which is consistent with, and more conservative than, the centroid-based estimate.

However, we note that the overlap-based estimate above is intentionally conservative, as it only requires that the two projected SNR shells intersect at any level. In contrast, the observed morphology suggests a substantially stronger geometrical association. The two remnants overlap over a large projected extent (Figs. 1, 2), and the eROSITA spectral analysis further suggests that the X-ray emitting structures may even completely overlap rather than merely intersect in projection^12^. A stricter geometric criterion can therefore be considered by requiring the smaller remnant (IC 443) to lie largely within the projected extent of G189.6+3.3. In this case, the centroid separation must satisfy *d* < *R*_G189_ − *R*_IC443_ rather than the more permissive overlap condition *d* < *R*_G189_ + *R*_IC443_. Adopting *R*_IC443_ = 0.375^∘^ and *R*_G189_ = 0.75^∘^ yields an effective containment area

$${A}_{{{{\rm{cont}}}}}=\pi {({R}_{{{{\rm{G189}}}}}-{R}_{{{{\rm{IC443}}}}})}^{2}\simeq 0.44\,{{{\rm{deg}}}}^{2},$$

which is approximately an order of magnitude smaller than the previously adopted overlap area. Using the same surface density of Galactic SNRs and applying the same distance-compatibility requirement gives a corresponding joint probability

$${P}_{{{{\rm{joint}}}}}^{{{{\rm{cont}}}}}=1{0}^{-3}.$$

Thus, the overlap-based estimate should be regarded as a conservative upper limit for any projected shell overlap, while the stricter containment-like configuration yields probabilities closer to the centroid-based estimate, at the level of 0.1%.

While necessarily approximate and not formal posterior probabilities, these estimates provide quantitative context for the likelihood of a chance spatial and distance coincidence: the centroid-centroid estimate yields a joint probability of 0.06%-0.2%, while the conservative size-aware overlap-based estimate results in a joint probability of 0.9% once distance compatibility is required (reduced to 0.1% under the stricter, and likely more appropriate, containment-like configuration for this system discussed above).

We note, however, that these probabilities may be inflated by two effects not explicitly incorporated into the estimates above. First, the Green SNR catalog is likely incomplete, so the true number of Galactic SNRs is likely larger than the 310 cataloged remnants used here. Second, IC 443 and G189.6+3.3 lie in the Gem OB1/S249 environment, where the local density of massive-star remnants may exceed the mean Galactic-plane value. These effects would increase the chance-alignment probability relative to the values quoted above.

Nevertheless, two considerations mitigate the impact of these effects. First, it is true that the expected Galactic SNR population inferred from supernova rates and typical SNR visibility times is often estimated to be of order 1000-1200 remnants (e.g.^58–60^). However, the relevant comparison population is not necessarily this full expected Galactic SNR population. The age at which an SNR dissipates into the surrounding medium depends strongly on the environment into which it expands, and many of the missing remnants are expected to be old, faint, highly evolved, morphologically disrupted, or difficult to identify as coherent shell-type structures with well-defined centroids, angular extents, and distance constraints. Second, adopting ∣*b*∣≤5^∘^ already accounts, at first order, for the concentration of Galactic SNRs toward the Galactic plane and therefore yields a more conservative surface density than an all-sky average, particularly given that the IC 443/G189.6+3.3 system lies toward the Galactic anticenter, where the surface density of known Galactic SNRs is substantially lower than in the inner Galaxy.

To nevertheless assess the impact of these uncertainties conservatively, we performed an illustrative sensitivity test. Allowing for an effective incompleteness correction of a factor of 2 − 4 for identifiable SNR shells and an additional local surface-density enhancement of a factor of 2 − 3 in the Gem OB1 region would inflate the stricter 10^−3^ probability by roughly one order of magnitude, to the 4 × 10^−3^− 1.2 × 10^−2^ range (0.4% − 1.2%). Thus, while catalog incompleteness and local clustering can increase the chance-alignment probability, the resulting probability remains significantly low ( ≲ 1%) even under assumptions that may overestimate the relevant correction factors.

It is important to note that the adopted correction factors are themselves intentionally conservative. Considering the broader Gem OB1 molecular-cloud complex, which extends over approximately 9^∘^ × 6. 5^∘^^61^ (*A*_Gem_ ≃ 58.5 deg^2^), and adopting the two currently identified remnants associated with the region (IC 443 and G189.6+3.3), the implied local surface density is *Σ*_Gem_ ≃ 2/58.5 ≃ 0.034deg^−2^. Even if the only other confirmed SNR within the broader projected Gem OB1 region, G190.9 − 2.2^62^, is included in the surface-density estimate, the implied local surface density becomes *Σ*_Gem_ ≃ 0.051deg^−2^, which remains lower than the Galactic-plane value adopted in our baseline estimate, derived assuming a Galactic-plane distribution within ∣*b*∣≤5^∘^.

To this end, if one considers only the effect of a larger underlying Galactic SNR population, adopting 1000 − 1200 expected remnants instead of the 310 cataloged objects increases the stricter 10^−3^ probability by a factor of 3.2 − 3.9, yielding 3.2 × 10^−3^ − 3.9 × 10^−3^ (0.3% − 0.4%). We also note that if one directly adopts the broader Gem OB1 molecular-cloud complex itself to estimate the local SNR surface density, this yields a value approximately a factor of 1.7 − 2.5 (based on the above estimates) lower than the Galactic-plane surface density adopted in our baseline estimate. In this case, even adopting an underlying Galactic SNR population of 1000 − 1200 remnants increases the stricter 10^−3^ probability only to the 1.3 × 10^−3^ − 2.3 × 10^−3^ range (0.13% − 0.23%).

In this context, the interpretation of a common binary origin is physically well motivated, given the low likelihood of a random alignment, the compatibility with binary population-synthesis results, and the high intrinsic multiplicity of massive stars (e.g.^57^ and references therein). Taken together, our results provide strong evidence for what may represent a system in which both components of a binary have undergone supernova explosions, extending the observational landscape of binary stellar evolution into a regime not yet represented by known remnants. While no single observational signature can unambiguously establish a binary progenitor origin in evolved SNRs, such systems must instead be assessed through the convergence of multiple, independent lines of evidence. In this case, the wealth of evidence form a mutually consistent set of observational and theoretical constraints that is difficult to reconcile with alternative scenarios.

Such systems are rare and provide a unique observational handle on binary evolution in the regime where both stars reach collapse. Their identification offers an opportunity to probe the late stages of stellar evolution in interacting massive binaries and to refine constraints on models of massive-star evolution, binary mass transfer, and the feedback of supernova explosions into their surrounding environments. In particular, such information can be employed to gain further insight into how mass transfers between the progenitor stars in the pair through the accretion process, as well as determine the energy budget (and the channels through which energy is distributed) of supernova explosions based on the angular separation between the centers of the two SNRs. The relative geometry of the two remnants and their shared environment can inform scenarios in which pre-supernova mass loss and strong stellar winds excavate low-density cavities in the ISM. Efficient hadronic gamma-ray emission may then be triggered when the expanding shock encounters dense material at the cavity boundary or at the edge of an HII region. The detection of distinct emission components within the same complex therefore provides a valuable testbed for addressing the longstanding leptonic–hadronic ambiguity in SNRs.

More broadly, by constraining the evolutionary pathways of massive binary systems, observations of this kind offer an important empirical framework for improving our understanding of stellar life cycles and the physical processes governing the formation, evolution, and explosive endpoints of massive stars. By constraining the evolutionary pathways of massive binary systems, such observations may also inform broader theoretical frameworks describing the end stages of stellar evolution. Further investigation into the potentially associated pulsars could yield additional information regarding the natal kick mechanism of compact objects produced by supernova explosions. In a broader context, this study demonstrates the value of integrating multiwavelength data to draw robust conclusions on the nature of SNRs and their progenitor origins, particle acceleration mechanisms, and energy injected into cosmic rays.

### Implications at very high energies

In this context, it is also important to consider how the presence of two partially overlapping remnants within the IC 443 complex affects the interpretation of very-high-energy (TeV) gamma-ray observations. TeV gamma-ray emission has been firmly detected from the IC 443 complex by multiple instruments. Imaging atmospheric Cherenkov telescopes, including the Major Atmospheric Gamma Imaging Cherenkov (MAGIC) telescopes, the Very Energetic Radiation Imaging Telescope Array System (VERITAS), and the High Energy Stereoscopic System (H.E.S.S.), report an extended TeV source spatially coincident with IC 443^63–66^. Observations with the wide-field High-Altitude Water Cherenkov (HAWC) Observatory detect a TeV source consistent with the remnant and, in deeper analyses, reveal evidence for additional extended emission in the surrounding region^67^. Subsequent observations with the Large High Altitude Air Shower Observatory (LHAASO) reported two gamma-ray sources within the angular extent of IC 443^68^. One of these, component C1, is at least partially spatially coincident with G189.6+3.3. However, because IC 443 and G189.6+3.3 partially overlap on the sky, and given the present limitations in angular resolution and source decomposition at multi-TeV energies, these detections cannot presently be uniquely associated with either remnant. While the TeV emission is conventionally attributed to IC 443, a contribution from G189.6+3.3 cannot be excluded. In addition, the HAWC upper limits^67^ are in tension with the LHAASO C1 signal^68^, likely reflecting differences in the assumed source morphologies, which also differ from the spatial templates used in this work to describe the *Fermi*-LAT source. For these reasons, we do not use the TeV measurements to constrain the spectral modeling presented in this work, but instead include them only in a qualitative comparison. Disentangling the relative contributions of the two remnants will require future TeV observations with improved angular resolution and dedicated spatial modeling. This represents another promising avenue for investigating this complex system.

## Methods

### LAT observations and data reduction

The LAT instrument aboard the *Fermi* spacecraft that collects photons in the energy range of 20 MeV to 1 TeV was employed for this study. We used approximately 16 years of *Fermi*-LAT Pass 8 Release 3 (P8R3) data^69,70^, analyzed with the latest instrument response functions (P8R3_SOURCE_V3), including both front- and back-converting events. The data span mission elapsed times from 239557417 to 750035897, corresponding to coordinated universal time from 4 August 2008 (15:43:36.0) to 7 October 2024 (23:18:12.0). All standard cleaning processes aimed at avoiding data contamination by transient events were followed. Bad time intervals were identified and excluded from the analysis. Earth limb contamination was effectively treated, by excluding data sets that exceed a zenith angle of 90^∘^ and 105^∘^, respectively, above 100 MeV and 1 GeV.

Data processing, including event selection and exposure calculations, was performed using the fermipy analysis package (v1.3.1)^71^, a Python interface to the fermitools software suite (v2.2.0; https://fermi.gsfc.nasa.gov/ssc/data/analysis/software/) designed primarily for binned analyses of *Fermi*-LAT data. The analysis employed up-to-date background models, including the Galactic diffuse emission (gll_iem_v07.fits) and the isotropic component accounting for residual instrumental and extragalactic backgrounds (iso_P8R3_SOURCE_V3_v1.txt).

The models were retrieved from the Fermi Science Support Center (FSSC; https://fermi.gsfc.nasa.gov/ssc/data/access/lat/BackgroundModels.html). All 4FGL-DR4 sources embedded within a region of 15^∘^ radius from the center of the region of interest (ROI) were included in the model. Following the above conventions, we performed a binned analysis over a 10^∘^ × 10^∘^ region centered at the G189.6+3.3 SNR (ROI). The spatial analysis was performed  > 1 GeV, and up to 800 GeV, while the spectral analysis was conducted in the broader 100 MeV-800 GeV energy range.

### Morphological analysis

#### eROSITA data

Due to the lack of a complete radio shell, the motivation behind this project arose from the newly acquired eROSITA data^72,73^. eROSITA is the main instrument aboard the Russian-German Spektrum Roentgen Gamma (SRG) observatory^74^. It collects photons in the 0.2-10 keV energy range and it consists of seven parallel aligned mirrors of 1^∘^ field of view (FOV) each (TM1-7). Reference ^12^ reported the first complete view of the G189.6+3.3 SNR with eROSITA. In this section, we describe the eROSITA imaging analysis used to construct spatial templates for the subsequent *Fermi*-LAT morphological analysis.

Aiming at a direct spatial comparison of the remnant in X-rays and gamma-rays and subsequently at examining the implications of such a spatial correlation in the two distinct energy bands, we employed publicly available data from the first eROSITA all-sky survey (eRASS1) in the c020 processing version. We performed standard data reduction, cleaning, and analysis processes employing the corresponding tools of eSASSusers_201009 version of eSASS (eROSITA Standard Analysis Software)^75^. We report on the eROSITA mosaic intensity map displayed on the left panel of Fig. 2 in the 0.2-5.0 keV energy band at and around the remnant’s position. The latter energy range was optimized based on spectral analysis results; these results, however, are beyond the scope of this paper. All point sources have been properly masked to improve visibility of diffuse X-ray emission from G189.6+3.3 and IC 443 SNRs. Extended diffuse X-ray emission from G189.6+3.3, which is completely thermal and has a distinct shell-type morphology that overlaps with the IC 443 to the west, is detected in eRASS1.

#### LAT data

We performed a binned spatial analysis, of 0.02^∘^ bin size,  > 1 GeV (since at lower energies the instrument PSF size exceeds 1^∘^), handling events types PSF0, PSF1, PSF2, and PSF3 separately in the SummedLikelihood framework. We simultaneously fitted the spectral parameters of all sources within 3^∘^ from the ROI center, along with the normalizations of the Galactic and isotropic background components, as well as the index of the Galactic diffuse component.

Three 4FGL-DR4 sources fall within the well-defined X-ray emitting shell extension detected with eROSITA, namely J0618.9+2240c, J0620.1+2246, and J0620.9+2201, as shown on the left panel of Fig. 2. An additional point source, namely 4FGL J0622.7+2248, lies just outside of the remnant’s X-ray emitting shell. Within a few degrees of the remnant, there are no other nearby Fermi sources. All four sources do not have known counterparts that would indicate potential association with other astrophysical objects. In the fitting process, GeV gamma-ray emission from all four 4FGL-DR4 sources was included to reproduce the emission from the G189.6+3.3 SNR.

To spatially inspect the GeV emission from the G189.6+3.3 SNR and to identify any additional sources within the ROI, we computed a test statistic (TS) map excluding from the model the four aforementioned *Fermi* point sources. As such, the significance of a source is examined with a standard E^−2^ spectrum at the position of each pixel against the null hypothesis (TS = 2(ln${{{{\mathcal{L}}}}}_{1}$-ln${{{{\mathcal{L}}}}}_{0}$), with ${{{{\mathcal{L}}}}}_{1}$ tested and ${{{{\mathcal{L}}}}}_{0}$ null hypothesis likelihoods). The obtained TS map displays gamma-ray emission from the site of the IC 443 remnant, which is most likely due to mismodeling of this prominent gamma-ray emitting SNR. At present, the gamma-ray emission from IC 443 is described using two catalog components: the extended source 4FGL J0617.2+2234e (associated with IC 443) and 4FGL J0616.5+2235 (a second gamma-ray source located within the angular extent of the remnant and also likely associated with SNR). An improved spatial template would be necessary to account for the apparent residuals, but such a study falls beyond the scope of this work.

Instead, to account for the apparent residuals and prevent mismodeling of IC 443 from affecting the analysis of G189.6+3.3 two additional point sources (RA_*J*2000_: 94.46^∘^, Dec_*J*2000_: 22.37^∘^ and RA_*J*2000_: 94.11^∘^, Dec_*J*2000_: 22.44^∘^) were added to the model. PS maps were generated and examined before and after the addition of the two point sources to ensure that no significant negative residuals emerged from their locations. A PS map aims to assess data with model agreement and is sensitive to both positive and negative deviations, allowing us to confirm the validity of the model by computing the p-value, which is more reliable than residual count maps^76^. After validating the background model using the PS maps, we recomputed the TS map above 1 GeV. The obtained TS map  > 1 GeV, shown on the left panel of Fig. 3, reveals a gamma-ray source to the east of IC 443.

However, the PSF size at the lower energy cut exceeds the source size. To confirm the extended nature of the detected GeV source and report on a clear statement of a separate gamma-ray source, which is not associated with IC 443, we restricted the spatial analysis  > 5 GeV (despite the lower statistics) to ensure an improved PSF size. The TS map  > 5 GeV, shown on the middle panel of Fig. 3, shows extended GeV emission, detected  > 10*σ* significance level to the northeast and at 10*σ* significance level to the southeast using the spatial template 13 - best fit spatial template as obtained from the morphological analysis performed in this work (refer to Table 1). This is perfectly coincident with the eastern part of the remnant’s well-defined X-ray emitting shell, as depicted in the composite image shown in Fig. 1. Furthermore, the clear separation of the GeV emission to the southeastern region of G189.6+3.3, which coincides with the enhanced X-ray emission at the eastern boundary of the remnant’s shell (Fig 1), strongly suggests the presence of a separate gamma-ray source rather than gamma-ray emission from IC 443 protons diffusing in the surrounding areas. This finding is further corroborated by the nature of the emission from the southeastern region, which appears to be leptonically induced, as discussed in subsections ‘Spectral analysis of the LAT data’ and ‘Multiwavelength SED modeling’.

**Table 1:** Gamma-ray spatial model comparison

	Spatial template	TS	LogLikelihood	d.o.f.	*Δ*AIC	Ra(^∘^), Dec(^∘^)	(R^∘^, *σ*)
(1)	No sources	–	93188.2	–	–	–	–
(2)	4 point sources	192.8+124.3+30.1+36.9	93387.0	16	0	–	–
(3)	2D Gaussian	249.2	93299.4	3	–149.1	94.98,22.15	0.47 ± 0.05
	2D Gaussian(free)	(506.7)	(93402.9)	(5)	(53.9)	(95.03 ± 0.03, 22.64 ± 0.02)	(0.26 ± 0.03)
(4)	Disk	255.5	93303.5	3	-140.9	94.98,22.15	0.81 ± 0.03
	Disk(free)	(460.8)	(93389.2)	(5)	(26.4)	(95.05 ± 0.03, 22.67 ± 0.03)	(0.4 ± 0.03)
(5)	eROSITA polygon	257.4	93309.6	2	–126.7	–	–
(6)	eROSITA image	262.8	93311.5	2	–123.0	–	–
(7)	eROSITA image + H*α* filament	136.4 + 219.8	93394.3	4	38.6	–	–
(8)	eROSITA polygon + H*α* filament	149.2 + 216.0	93395.2	4	40.4	–	–
(9)	Disk + H*α* filament	179.3 + 197.2	93404.9	7	53.8	95.21 ± 0.05, 22.32 ± 0.04	0.86 ± 0.03
(10)	eROSITA image + 2D-Gaussian	63.6 + 422.2	93428.5	7	101.0	94.91 ± 0.02, 22.67 ± 0.02	0.19 ± 0.02
(11)	Disk + Disk	78.5 + 490.4	93432.4	10	102.9	95.16 ± 0.04, 21.90 ± 0.04+94.90 ± 0.02, 22.67 ± 0.02	0.54 ± 0.03+0.32 ± 0.02
(12)	Disk + 2D-Gaussian	70.6 + 501.1	93437.6	10	113.2	95.16 ± 0.04, 21.89 ± 0.04+94.92 ± 0.02, 22.67 ± 0.02	0.54 ± 0.03+0.20 ± 0.02
(13)	eROSITA polygon + 2D-Gaussian	85.3 + 418.6	93437.2	7	118.4	94.90 ± 0.02, 22.66 ± 0.02	0.19 ± 0.03

A comparison of the goodness of fit of different spatial templates tested  > 1 GeV. The first column denotes the model number. The second column outlines the model tested. The third column presents the TS values obtained for each model component. The fourth column displays the likelihood values obtained for each model tested. The fifth column indicates the degrees of freedom of each model tested. The sixth column reports the computed *Δ*AIC. The seventh and eighth columns provide position and extension, respectively, when applicable. In parentheses, results obtained by allowing the source position to vary (position fitting/localization) are reported for the single-component Models 3 and 4 and are denoted as free. Where applicable, all two-component models were allowed to vary in position and extension.

Since the gamma-ray emission conventionally associated with IC 443 is predominantly interpreted as hadronically induced^26^, the presence of a distinct leptonic component further supports the interpretation that the detected emission originates from a separate source associated with G189.6+3.3 rather than from particles escaping IC 443. A similar conclusion is also suggested by the morphology of the northern GeV component. The enhanced GeV emission is not broadly distributed around IC 443 or simply correlated with external gas illuminated by runaway particles, but is instead spatially confined to the northern boundary of G189.6+3.3, where it coincides with the H*α* filament, UV emission, optical forbidden-line diagnostics of radiative shocks, and dense material associated with S249. This close correspondence with local shock tracers favors gamma-ray production at the G189.6+3.3/S249 shock-cloud interface rather than interaction of unrelated surrounding material with particles escaping from IC 443.

To further strengthen our argument regarding a separate gamma-ray source and provide a direct comparison with^13^, we extended the spatial analysis to even higher energies and present the remnant’s TS map above 10 GeV – where the substantially improved *Fermi*-LAT PSF ( ≲ 0. 1^∘^ containment radius) allows a much clearer spatial separation from IC 443, as illustrated on the right panel of Fig. 3. In addition, we construct a substantially larger 5. 7^∘^ × 5. 7^∘^*Fermi*-LAT PS map  > 10 GeV, as shown on the right panel of Supplementary Fig. 3, without modeling the gamma-ray emission from the IC 443 region. This map provides compelling evidence for the presence of an extended source to the east and southeast of IC 443, thereby excluding the possibility of IC 443 mismodeling that could result in residual emission in its vicinity, which might be erroneously interpreted as gamma-ray emission from G189.6+3.3.

Overall, Fig. 3 and Supplementary Fig. 3 (right panel) provide a firm detection of gamma-ray emission from the G189.6+3.3 SNR that appears to be in spatial coincidence with the eastern half of the well-defined X-ray shell detected with eROSITA. A particularly notable feature of this gamma-ray emission morphology is the pronounced enhancement coincident with the H*α* filament (shown as a cyan contour in Fig. 3), which appears clearly separated from IC 443 and more prominent than the gamma-ray emission from the remainder of G189.6+3.3. This feature will be discussed further in a subsequent section.

Having established the presence of extended gamma-ray emission spatially associated with G189.6+3.3, we next investigated its morphology in greater detail by first removing the 4FGL sources within the region and iteratively introducing alternative spatial source models. We explored several single-component spatial templates, including a 2D Gaussian, a radial disk, the eROSITA X-ray image (right panel of Fig. 2), and a flat (on-source region: pixel values set to 1, off-source region: pixel values set to zero) polygonal region that encompasses the X-ray emission detected with eROSITA (highlighted as a green contour on the right panel of Fig. 3 and in Supplementary Fig. 3). For the 2D-Gaussian and the radial disk templates (single source geometrical models), we initially set the added source center coordinates to (RA_*J*2000_: 94.98^∘^, *Dec*_*J*2000_: 22.15^∘^; moderately shifted to the east compared to the center of the X-ray shell since we only focused on the analysis of gamma-ray emission from the eastern half of the remnant, where there is no contamination from IC 443) and performed both a position and extension fitting and an extension-only fitting by computing a likelihood ratio test with respect to the no-extension (point-source) hypothesis. We note that fitting both the position and extension of the 2D-Gaussian and radial disk templates results in the center being shifted towards the northern brighter blob and the extension, sigma or radius, being significantly reduced (localized to the region of the northern bright blob). Consequently, a portion of the gamma-ray emission from the southern region of the remnant remains inadequately modeled. The resulting positions and extensions for the 2D-Gaussian and radial-disk models are summarized in Table 1. The Akaike Information Criterion (AIC)^77^ is used to compare the goodness of the fit between the different tested models as follows, ΔAIC=${{{{\rm{AIC}}}}}_{4{{{\rm{point}}}}{{{\rm{sources}}}}}-AI{C}_{i}=2(\Delta d.o.f.-\Delta ln{{{\mathcal{L}}}})$. According to both the fit quality (refer to Table 1) and the residual count maps, no single-component model can simultaneously reproduce the southern extension of the gamma-ray emission and the strong concentration of emission observed at the northern boundary of the remnant.

Consequently, since single-component models fail to adequately reproduce the observed gamma-ray morphology, and motivated by the apparent flux variation between the northern and southern regions below 10 GeV as well as by the fact that only the northern part overlaps with the S249 HII region, we tested several two-component, hereafter named G189N and G189S, source models (models 7–13 in Tabl. 1) to determine the morphology that best reproduces the observed emission. The implications of such a treatment in the characterization of the particle population(s) responsible for the gamma-ray radiation (hadronic and/or leptonic) are discussed in subsection ‘Spectral analysis of the LAT data’. The two spatial templates are motivated by distinct physical and geometric considerations. The southern component is defined by the large-scale geometry of the SNR shell as traced by the eROSITA X-ray emission, whereas the northern component is motivated by the presence of a localized H*α* filament tracing the interaction with dense gas associated with the S249 HII region. For this reason, the H*α* filament itself was the first spatial template tested for the northern gamma-ray emission. Notably, the H*α* template used in some of those two-component templates was constructed similarly to the flat eROSITA polygonal template by setting pixel values to 1 on filament regions and zero elsewhere.

In this framework, the terms “northern” and “southern” are used as descriptive labels indicating the relative spatial location of the dominant emission regions rather than to imply strictly separate extraction areas. The two components partially overlap (see Fig. 3), and this overlap is explicitly accounted for in the likelihood analysis. Specifically, the three *Fermi*-LAT point sources located toward the northeastern part of the remnant are replaced by a single extended source represented by a conspicuous optical filament detected at the northern part of the remnant. A second extended component, modeled using either the eROSITA X-ray emission map and/or a radial disk template, is assigned to the southeastern part of the remnant, replacing the fourth *Fermi* point source, which is molecular gas-free. It is noteworthy that the observed emission from the southern part of the remnant is detected at approximately 10*σ*,   > 1 GeV. The variations in the TS values obtained for the gamma-ray emission detected at the southern region of the remnant, as reported in the second column of Table 1, are contingent upon the model applied to the northern region with the addition of the second component (the H*α* filament, a second disk, and a 2D-Gaussian).

From the morphological analysis, we conclude that despite the apparent spatial correlation of the X-ray shell with the diffuse GeV emission, the X-ray eROSITA template alone, shown on the right panel of Fig. 2, after properly masking IC443’s X-ray emission, was not obtained as the best fit spatial model to the GeV gamma-ray data. This is primarily because the gamma-ray emission appears to extend further to the north of the X-ray shell, the X-ray emission is purely thermal (i.e., the regions of enhanced emission for X-rays and gamma-rays differ), and the gamma-ray emission appears strongly enhanced at a specific position at the north edge of the remnant. Among the above tested models, the best ΔAIC value (significantly improved compared to four point sources or single source models that account for emission from the entire remnant) is obtained while adopting a spatial template that consists of two distinct but overlying components: the polygonal region obtained from the eROSITA X-ray image of the remnant (primary contributions from its southern part) and a 2D-Gaussian at its northern boundary, spatially coincident with the H*α* filament. A 5. 7^∘^ × 5. 7^∘^ PS map,  > 1 GeV, was generated and examined after running the analysis with our best fit spatial model to ensure that no significant positive or negative residuals emerged from the nearby locations (refer to the left panel of Supplementary Fig. 3). The latter model demonstrates that the gamma-ray emission is spatially aligned with the eROSITA X-ray shell and peaks at the location of the filament (center of the north bright blob). This result is evident of particle acceleration within the filament and subsequent diffusion in the surrounding medium, as further discussed in subsection ‘Spectral analysis of the LAT data’ and subsection ‘Multiwavelength SED modeling’.

To further visually demonstrate that the gamma-ray emission from G189.6+3.3 is more accurately described by extended source components rather than by the currently adopted four-point-source representation, we investigated the residual emission obtained under different source-modeling configurations. Specifically, we constructed TS maps  > 1 GeV by removing one, two, three, and then all four *Fermi* sources within the region of interest: 4FGL J0618.9+2240c detected with TS=192.8, 4FGL J0620.1+2246 detected with TS=124.3, 4FGL J0620.9+2201 detected with TS=30.1, and 4FGL J0622.7+2248 detected with TS=36.9. This procedure reveals significant residual emission from the southeastern part of the shell, as shown in the left panel of Supplementary Fig. 1. Even when both 4FGL J0618.9+2240c and 4FGL J0620.1+2246 sources are modeled (the two Fermi sources with the highest significance among the four), diffuse gamma-ray emission is detected from the remnant’s eastern half  > 10*σ* level. In fact, even after modeling all four *Fermi* sources, strong residuals in the form of diffuse emission from the eastern and southeastern half of the shell persist ( > 10*σ*), as shown on the left panel of Supplementary Fig. 1. In contrast, no comparably significant residual structures remain when adopting the best-fit extended spatial model discussed above (right panel of Supplementary Fig. 1). This direct comparison further validates the significant improvement in goodness of fit (given in Table 1) when utilizing extended models compared to the currently adopted four-point-source model.

### Spectral analysis of the LAT data

A two-component source model needs to be employed to account for the entire gamma-ray emission from the remnant’s location. To this end, the G189N (originating from the long H*α* filament but best represented by a 2D-Gaussian) and the G189S (represented by the polygonal region obtained from the eROSITA X-ray image - illustrated as a green contour on the right panel of Fig. 3 and in Supplementary Fig. 3) spatial templates were employed to investigate the nature of the gamma-ray emission from different parts of the remnant (spatial model 13), as discussed in subsection ‘LAT data’. The spectral analysis is performed in the broader 100 MeV to 800 GeV energy range.

Two additional years of data compared to 4FGL-DR4 have been employed for the purposes of this study. To account for potential additional faint sources not included in the 4FGL-DR4 catalog and a potential change of the morphology of the gamma-ray emission from the remnant, we construct and inspect the TS map from the remnant’s location  > 100 MeV. The shape of the gamma-ray emission from the remnant’s location remains intact - nearly identical to the one obtained  > 1 GeV. In addition, no new sources within 2^∘^ from the analysis ROI were detected that could directly contaminate our spectral data, thus no additional entries were included in our model prior to running the spectral analysis process.

As a next step, we tested both a simple power-law and a LogParabola spectral model for each component, using the curvature test statistic (TS_curv_) implemented in fermipy to quantitatively discriminate between them. The TS_curv_ is defined as twice the difference in the log-likelihood between a curved spectral model (e.g. LogParabola) and a simple power-law model. We find that a powerlaw emerges as the best-fit spectral model for G189S. Conversely, a LogParabola describes best the energy distribution of the gamma-ray photons from G189N. The corresponding spectral parameters of the north and south components are summarized in Table 2.

**Table 2:** Gamma-ray spectral parameters

Spatial template	Spectral model	Index	Energy Flux
			MeV cm^−2^ s^−1^
G189N (2D-Gaussian)	LogParabola	1.45 ± 0.13 ± 0.15, 0.12 ± 0.03 ± 0.04	2.1 ± 0.13 ± 0.15 × 10^−5^
G189S (eROSITA polygon)	Powerlaw	1.60 ± 0.08 ± 0.08	1.24 ± 0.21 ± 0.26 × 10^−5^
G189S (Disk)	Powerlaw	1.55 ± 0.1 ± 0.1	8.86 ± 1.75 ± 1.62 × 10^−6^

Best fit spectral parameters of the G189N and G189S remnant’s components in the 100 MeV to 800 GeV energy range. The spectral parameters of the LogParabola fitting were obtained at E_b_ = 100 MeV. The first error represents statistical error whereas the second is systematic uncertainty.

We then divided the 100 MeV to 800 GeV energy range into eight logarithmically spaced bins, with the highest-energy bin covering a narrower interval of 320–800 GeV. A likelihood fitting analysis was performed to derive the gamma-ray SED by computing the photon flux in each bin separately for the G189N and G189S components, with the aid of a model that contains both the G189N and G189S components (model 13) instead of the entire source. In the above process the spectral index, and *α*,  *β* spectral parameters of the source components (where applicable) were kept fixed to the best fit values. The normalizations of all 4FGL-DR4 sources within 3^∘^, as well as the normalizations of the background components, were let to vary. We set a TS threshold of 4 and provide 95% upper limits for intervals that exhibit lower values. The derived spectral points for both source components are illustrated in Fig. 4.

Regarding the systematics, we considered three distinct types of systematic uncertainties and added them in quadrature. These encompass uncertainties in the Galactic diffuse background, which primarily affect the lower energy regime; uncertainties in the effective area; and uncertainties in the optimal spatial model. To determine the former two types of uncertainties, we adhere to the methodology delineated in ref. ^78^. The latter source of systematic uncertainties is typically evaluated by extracting the spectrum for the second-best fit model, which in this case comprises a radial disk to the south and a 2D-Gaussian to the north (model 12). However, to avoid underestimating the systematic error, given that the 2D Gaussian template used to model G189N in model 12 is nearly identical, we opted for model 11 instead, which is not significantly inferior to model 13. The resultant SED, incorporating both statistical and systematic uncertainties, is presented in red in Fig. 4.

### Estimates on the remnants’ intrinsic properties

#### Distance estimates

*G189.6+3.3:* Typical luminosities of SNRs at 1.4 GHz have values in the range of 5 × 10^14^ < *L*_1.4GHz_ < 10^17^ W/Hz^79^. To measure a lower limit for the distance, it is assumed that G189.6+3.3 has *L*_1.4GHz_ = 5 × 10^14^ W/Hz, yielding a ${d}_{\min }=1.4$ kpc. A maximum distance estimate can also be provided considering that SNRs typically do not exceed a diameter of 100 pc^80^. Thus, given the angular size of G189.6+3.3 (approximately 1. 6^∘^ in diameter based on its visible eROSITA X-ray shell), we estimate a maximum distance of ${d}_{\max }=3.5$ kpc. We note that a slightly larger angular extent of about 1. 8^∘^ is suggested by eROSITA spectral evidence indicating that G189.6+3.3 and IC 443 completely overlap^12^.

Additionally, the absorption column density values derived from the remnant’s eROSITA X-ray analysis (X-ray spectral fit)^12^ and the up-to-date 3D extinction maps obtained from the combination of the 2MASS and GAIA data^21^ were utilized to provide an additional independent distance consistency check. Reference ^81^ employed a large sample of Chandra observations of Galactic SNRs to derive a statistical relation between the X-ray absorption column density (*N*_H_) and the mean color excess (*E*_B-V_)/extinction (*A*_ν_), as follows:

1 $${N}_{{{{\rm{H}}}}}/{E}_{{{{\rm{B-V}}}}}=8.9\times 1{0}^{21}\,{{{{\rm{cm}}}}}^{-2}\cdot {{{{\rm{mag}}}}}^{-1}\\ {N}_{{{{\rm{H}}}}}/{A}_{{{{\rm{\nu }}}}}=2.87(\pm 0.12)\times 1{0}^{21}{{{{\rm{cm}}}}}^{-2}\cdot {{{{\rm{mag}}}}}^{-1}$$

Considering the X-ray absorption column density measured for G189.6+3.3, *N*_H_ = 4 × 10^21^ cm^−2^, which is found to be rather uniform across the remnant^12^, we derive an optical extinction of ${A}_{\nu }=1.3{9}_{-0.05}^{+0.06}$. The quoted uncertainty accounts for the range of column densities, *N*_H_ = (3 − 6) × 10^21^cm^−2^, obtained from the spatially resolved X-ray spectral analysis of different sub-regions and spectral models across the remnant. Inputting such a result in the 3D extinction maps of^21^, implemented via the EXPLORE G-Tomo tool, yields a distance uncertainty of ${{{\rm{d}}}}=1.{8}_{-0.04}^{+0.06}$ kpc (gray shaded region on the right panel of Fig. 8). This measurement was derived by considering the cumulative extinction estimate towards the center of the remnant.

However, because G189.6+3.3 is an extended source, it is important to assess how sensitive this estimate is to the specific line of sight adopted. The EXPLORE G-Tomo tool is a visualization and query interface that extracts line-of-sight extinction profiles and two-dimensional slices from up-to-date three-dimensional dust extinction maps. Because G-Tomo is intrinsically a pencil-beam estimator (i.e., it applies to a single set of sky coordinates), its application to an extended source requires some care. We therefore queried the extinction-distance relation at multiple positions across the angular extent of G189.6+3.3 in order to assess how strongly the inferred distance varies across the remnant and to quantify the associated systematic uncertainty. Specifically, conducting the distance estimation by considering the extinction observed towards the eastern boundary of the SNR results in a distance estimate closer to 1.9 kpc. The large majority of positions sampled across the remnant yield distance estimates clustered between 1.8 and 1.9 kpc, indicating that this interval can be considered a confident distance estimate for G189.6+3.3. By incorporating the uncertainty in the cumulative extinction and the full range of positional variation across the SNR area, a broader distance uncertainty range of 1.7 to 2.3 kpc can be obtained.

*IC 443:*

The distance to IC 443 based on kinematic measurements of optical H*α* emission is estimated to be approximately 1.9 kpc^82^, while independent extinction-based measurements yield a consistent distance of 1.8 ± 0.05 kpc^83^. A general 1.5–2.0 kpc distance for IC 443 is broadly used in the literature (e.g.,^84^, and references therein).

Claims that the S249 HII region lies several hundred parsecs in front of IC 443 are based on kinematic distance estimates inferred from small differences in radial velocity^82^. However, near the Galactic anti-center the velocity-distance relation is shallow, and kinematic distances become intrinsically uncertain and highly sensitive to modest non-circular motions and projection effects. As such, in this direction small differences in radial velocity—such as those reported for S249 and IC 443 in ref. ^82^—do not map cleanly to distance. In this regime, assigning a unique systemic velocity to S249 is model-dependent, particularly along a line of sight that contains multiple kinematic components.

In contrast, multiple observational tracers point to a physical association between IC 443 and S249, including the morphological correspondence between the northeastern rim/optical filaments of IC 443 and the S249 HII region^37^, and enhanced radio/continuum brightness and morphological distortion of IC 443 toward the northern (S249-facing) side^46^. In addition, gamma-ray emission from the IC 443 environment is widely interpreted as arising from particle acceleration followed by interactions with dense gas in its surroundings^44^, consistent with the presence of a dense, structured medium in the IC 443-S249 complex^45^. When combined with the detected gamma-ray emission from G189.6+3.3 reported here, these observations strongly support a common distance for IC 443, S249, and G189.6+3.3.

#### Age estimates

*G189.6+3.3:* There is no single precise, widely-accepted age for G189.6+3.3 in the literature; however, the available estimates consistently classify it as a middle-aged or evolved SNR, older than IC 443. The initial age of G189.6+3.3 was estimated to be around 10^5^ years based on its X-ray appearance and the very soft emission detected in ROSAT data^9^. This estimate was, however, derived from empirical considerations and is subject to large uncertainties. Assuming instead that the remnant is in the Sedov–Taylor phase, the same work reports a younger age of 3 × 10^4^ yr. Subsequent X-ray observations with Suzaku revealed recombining plasma along the eastern edge of the remnant, providing clear evidence that G189.6+3.3 is in a relatively advanced evolutionary stage^11^. Such recombining plasma signatures are commonly observed in middle-aged SNRs, including IC 443^85^. Taken together, these observational constraints favor an age of order a few  × 10^4^ yr for G189.6+3.3. However, it is important to note that, depending on the ambient density and thermal history, recombining plasma features may also persist in older remnants; and therefore an age of order 10^5^ yr remains a reasonable estimate for this SNR.

In this work, we employed different methodologies aiming at providing updated age estimates. From radio data, one can estimate an upper limit for the age of the remnant when knowing its linear radio extension (R=22.6 pc, assuming a distance of 1.8 kpc as computed above) and applying^86^ model, which provides an age estimate of: $t={({R}_{pc}/21.9)}^{3.23}\times 1{0}^{5}=1.1\times 1{0}^{5}$ yr. We additionally applied the python calculator for SNR evolution as provided in ref. ^87^ to perform an updated age estimation. For the obtained absorption column density of the entire remnant, *N*_H_ = 4 × 10^21^cm^−2^ and a distance of 1.8 kpc, we derive an average ISM number density of *n*_0_ = 0.72 cm^−3^. This value represents the local density at the location of the SNR, under the assumption that the line-of-sight average density is representative of the ambient medium. Considering as inputs the derived local ISM density as calculated above, a typical explosion energy on the order of 10^51^ erg and maintaining the remaining parameters at default input values, we derived an age of 4.5 × 10^4^ yrs. This value is an approximation since the density at the region of G189.6+3.3 can be significantly impacted by the local medium at the remnant’s distance. As such, we also adopt 0.5 cm^−3^ and 0.15 cm^−3^ as the ISM ambient density, as computed in ref. ^11^ at the distance of the remnant, which yields ages of 3.2 × 10^4^ yr and 1.8 × 10^4^ yr, respectively.

Finally, we confirmed the aforementioned results by performing a series of computations based on the Sedov-Taylor self-similar solution as outlined in ref. ^88^:

2 $$R=0.314\cdot {\left(\frac{{E}_{51}}{{n}_{0}}\right)}^{\frac{1}{5}}\cdot {t}_{{{{\rm{yr}}}}}^{\frac{2}{5}}\,{{{\rm{pc}}}},$$

where R is the remnant’s linear radius (shock radius), *E*_51_ the kinetic energy of the explosion in units of 10^51^ erg, *n*_0_ the ambient density, and *t*_yr_ the remnant’s age. Assuming that the SNR is still in the Sedov phase, we obtained an age estimate of 3.1 × 10^4^ yr for *n*_0_ = 0.5 cm^−3^ and 1.7 × 10^4^ yr for *n*_0_ = 0.15 cm^−3^.

We emphasize that both the Sedov-Taylor relations and the SNR evolution calculator^87^ provide global, average estimates of the time since explosion. These approaches are not intended to describe the detailed dynamical state of individual regions within the remnant. In an inhomogeneous environment, shocks propagating into dense material can locally decelerate and enter the radiative regime significantly earlier than shocks expanding into lower-density regions, while still originating from the same explosion at the same epoch. Consequently, the presence of a locally radiative shock at the interaction region at the northern boundary of the SNR does not contradict the use of a Sedov-like framework to estimate the global explosion age of the remnant.

For the present analysis, the quantity of primary relevance is the explosion epoch, as this constrains the possible time delay between the supernova events associated with G189.6+3.3 and IC 443. We therefore adopt a 20–110 kyr age estimate for G189.6+3.3. Using this age range, one can compute the shock speed as follows:

3 $${\upsilon }_{s}=\frac{2}{5}\times \frac{R}{t},$$

obtaining *υ*_s_ = 285 km/s for *n*_0_ = 0.5 cm^−3^ and *υ*_s_ = 520 km/s for *n*_0_ = 0.15 cm^−3^. Both sets of (*n*_0_, *v*_*s*_) estimates, together with the assumed distance, were employed to perform the multiwavelength SED modeling, as outlined in subsection ‘Multiwavelength SED modeling’.

*IC 443:*

The age of IC 443 has been calculated to range between 3 − 30 kyr^38,47,54,89–91^. On the lower limit, ref. ^89^ proposed an age of 3 kyr based on the shock velocity suggested by X-ray measurements of the remnant. However, the measured plasma temperature does not probe the current shock velocity. In fact, shock velocities derived from optical filaments imply a significantly older age of order 2 × 10^4^ yr age. A comparably young age of 4 kyr was also derived by ref. ^54^ under the assumption that the SNR is expanding within a pre-existing cavity. On the upper limit, ref. ^90^ proposed an age of 3 × 10^4^ yr under the assumption that the SNR is expanding in a dense medium by using a model for shock propagation in dense molecular material. Additionally,^91^ obtained a reliable age estimate of 3 × 10^4^ yr for what is believed to be the associated neutron star to IC 443. Finally, ref. ^38^ reported an age estimate of 2 × 10^4^ yr assuming a break out SNR. The most recent age estimation of IC 443 at the time of this study is derived from the dynamical modeling analysis presented in ref. ^47^, wherein the authors developed a 3D Hydrodynamical model to elucidate the interaction between IC 443 and its environment. This study concludes that the remnant’s age is approximately 8–9 kyr.

Comparing the aforementioned methods utilized in the various studies for age estimation, it is evident that an age as young as 3–4 kyr can be justified only if the SNR explodes in a large cavity. In the absence of a pre-explosion cavity formation, the 3–4 kyr estimate serves merely as a lower limit on the age of IC 443. Indeed, the morphology of the remnant supports an explosion in a dense medium where ejected material got trapped. Specifically, IC 443 presents as a two-shell asymmetric structure, with the two shells exhibiting different sizes. The larger radio shell expanding towards the southwest demonstrates a smooth decline in its radio continuum intensity radial profile. This indicates that the shock towards the southwest is propagating in a relatively uniform medium after breaking out, whereas the northeastern shell continues to expand in dense medium. The northeastern shell exhibits an inverse radio continuum intensity radial profile that peaks at its northeastern boundary as it continues to interact with dense medium, resulting in a smaller size. This preferred scenario, supported by observational data and consistent with the 3D Hydrodynamical model established to describe the evolution of IC 443^47^, provides an age estimate of approximately 8–9 kyr, which aligns well with the likely associated neutron star.

In synthesizing literature reports and the estimates presented in this study, an age uncertainty range of 3–30 kyr and 30–100 kyr has been identified for IC 443 and G189.6+3.3, respectively. This indicates that IC 443 represents the more recent explosion within the system, with a discernible time delay of up to approximately 100 kyr between the two supernova explosions.

### Multiwavelength SED modeling

In this section, we investigate the physical origin of the gamma-ray emission from G189.6+3.3 through multiwavelength SED modeling. Given the distinct spectral and multiwavelength characteristics of the northern and southern regions, we model the G189N and G189S components separately. The sparseness of the available radio data which are only indicative of the non-thermal nature of the radio emission (radio synchrotron radiation), as reported in ref. ^10^, together with the absence of a detected non-thermal X-ray component, limits the degree to which a fully constrained multiwavelength SED modeling of the SNR can be achieved. We nevertheless carry out multiwavelength modeling to probe the origin and physical properties of the gamma-ray emission and to strengthen our conclusions.

Consequently, we utilized data from the DRAO synthesis telescope and obtained a flux density of 2.0 Jy at 1.4 GHz for the entire SNR. This value is treated as an upper limit; the radio flux-density extraction is discussed in subsection ‘Proton re-acceleration’. The data quality at 0.4 GHz does not permit a similar estimate. Furthermore, despite the purely thermal nature of the X-ray emission from the remnant’s location, we computed an upper limit on the non-thermal X-ray flux of 1.75 × 10^−12^ erg/cm^2^/s in the 2.3–5.0 keV energy range. This estimate assumes an absorbed power-law model with a typical photon index of *Γ* = 2 and a column density of *N*_H_ = 4 × 10^21^ cm^−2^, as inferred from the X-ray spectral fit to the thermal emission.

We note that the absence of a detected non-thermal X-ray synchrotron component is not unexpected, even in the presence of a relatively strong magnetic field. In an evolved SNR interacting with dense material, synchrotron-emitting electrons are expected to suffer strong radiative losses and rapid spectral steepening, shifting any non-thermal X-ray emission to energies above the range where eROSITA is most sensitive. Indeed, eROSITA is optimized for the soft X-ray band, with a rapidly declining sensitivity and effective area above 3 keV^73^. Moreover, the dominance of thermal X-ray emission in the eROSITA band implies that any synchrotron component, if present, must lie below the derived upper limits and therefore remains subdominant compared to the thermal X-ray flux.

Consistent with this expectation, and given the age of the remnant, an X-ray synchrotron component might be expected to decrease sharply with a *Γ* = 3 spectral index. We therefore adopted, for the multiwavelength SED modeling, an alternative spectral model consisting of an absorbed power law with *N*_H_ = 4 × 10^21^ cm^−2^ and *Γ* = 3, which yields an upper limit on the non-thermal X-ray flux of 1.66 × 10^−12^ erg/cm^2^/s in the 2.3–5.0 keV energy range, corresponding to a discrepancy of approximately 5% relative to the flatter spectral assumption. To derive the upper limits for our preferred spectral model, we used the methodology outlined in ref. ^92^, and specifically in Appendix A.

Contextualizing these measured fluxes in combination with the obtained Fermi-LAT SEDs, we performed a multiwavelength SED modeling using the *naima* package for non-thermal radiative modeling^93^, which yielded results consistent with a hadronic-dominated gamma-ray component for the northern part and a leptonic-dominated gamma-ray component for the southern part. We explain below our SED modeling which uses the distance of 1.8 kpc derived in subsection ‘Estimates on the remnants’ intrinsic properties’. For both components, G189S and G189N, we adopted the simplest assumption that GeV and TeV gamma rays are emitted by a population of accelerated protons and electrons distributed in the same region characterized by constant density and magnetic field strength. We described the proton spectrum by a power law with an exponential cut-off (defining the maximum energy reached by the particles) and the electron spectrum by a broken power law with an exponential cut-off. The break in the electron spectrum is due to synchrotron cooling, and occurs at the energy *E*_*b*_ above which the particle spectral index is *s*_e,2_ = *s*_e,1_ + 1, with *s*_e,1_ being the spectral index below the energy break. We emphasize that this break should be regarded as a phenomenological, volume-averaged representation of radiative losses, rather than a sharp physical discontinuity, as spatial and temporal variations in the shock and magnetic field are expected to smear out such features in more realistic multi-zone or hydrodynamical descriptions.

To make the derivation of the principal model parameters explicit, we briefly summarize the key relations used to estimate the characteristic particle energies. Following the procedure described in detail in ref. ^88^, the electron cooling break and the maximum particle energies are obtained by comparing the relevant acceleration and radiative loss timescales with the characteristic age of the remnant, as commonly adopted in standard one-zone broadband SED modeling of SNRs. In the framework of DSA, the acceleration time can be written as

4 $${t}_{{{{\rm{acc}}}}}=30.6\,{k}_{0}\left(\frac{E}{1\,{{{\rm{TeV}}}}}\right){\left(\frac{B}{100\mu {{{\rm{G}}}}}\right)}^{-1}{\left(\frac{{v}_{s}}{1000{{{\rm{km}}}}{{{{\rm{s}}}}}^{-1}}\right)}^{-2}{{{\rm{yr}}}},$$

where *E* is the particle energy, *B* is the magnetic-field strength, *v*_*s*_ is the shock velocity, and *k*_0_ represents the ratio between the particle mean free path and the gyroradius. The synchrotron cooling time for relativistic electrons is given by

5 $${\tau }_{{{{\rm{sync}}}}}=1.25\times 1{0}^{3}{\left(\frac{E}{1{{{\rm{TeV}}}}}\right)}^{-1}{\left(\frac{B}{100\mu {{{\rm{G}}}}}\right)}^{-2}{{{\rm{yr}}}}.$$

The cooling break energy of the electron spectrum is obtained by equating the synchrotron cooling time with the age of the remnant,

6 $${\tau }_{{{{\rm{sync}}}}}({E}_{b})={t}_{{{{\rm{age}}}}}.$$

The maximum particle energy is determined by equating the acceleration time to either the remnant age or the dominant loss timescale,

7 $${t}_{{{{\rm{acc}}}}}({E}_{\max })=\min \left({t}_{{{{\rm{age}}}}},\,{\tau }_{{{{\rm{sync}}}}}\right).$$

For protons we consider the age-limited case, while for electrons the maximum energy is obtained by equating the acceleration and synchrotron loss timescales, *t*_acc_(*E*_max,e_) = *τ*_sync_(*E*_max,e_). These estimates depend on the unknown ratio between the mean free path and the gyroradius (*k*_0_). For young SNRs, we expect *k*_0_  ≃ 1, while for evolved systems, we expect *k*_0_ > 1. Below, we fixed this value at 1, providing therefore an upper limit on the derived maximum energy. In subsection ‘Age estimates’, we derived the age / velocity estimates of 3.1 × 10^4^ yr / *υ*_s_ = 285 km/s for *n*_0_ = 0.5 cm^−3^, and 1.7 × 10^4^ yr / *υ*_s_ = 520 km/s for *n*_0_ = 0.15 cm^−3^. These values are used below to constrain the characteristic particle energies in the SED modeling.

For the G189S component, the constraining radio upper limit implies a low magnetic field of *B* = 5μG and a hard injection index of 2.0 consistent with DSA. The shock speeds derived above for the remnant imply *E*_max,p_ = 4.2 TeV and *E*_max,p_ = 7.5 TeV, for densities of 0.5 cm^−3^ and 0.15 cm^−3^, respectively. When adopting these two distinct density/shock speed values, the only resulting difference is in the maximum energy (*E*_max,p/e_), which has a negligible impact on the resulting SED. For this reason, we adopt an intermediate value of 5 TeV. The physical parameters used to reproduce the multiwavelength data for the G189S component are summarized in Supplementary Table1 as representative solutions, while the modeled SED is presented in Fig. 5, showing that the dominant radiation at gamma-ray energies is IC scattering of accelerated electrons on ambient photon fields due to the low density of the medium in this region which suppresses proton-proton interaction. Very-high-energy (TeV) measurements from HAWC and LHAASO are shown for qualitative comparison only and are not included in the spectral modeling, owing to current uncertainties in source morphology and association within the IC 443 complex. We note that while such a low magnetic field implies long synchrotron cooling times and could in principle allow for electron escape, efficient escape would be expected to produce a broader or spatially displaced gamma-ray morphology, which is not observed. Within the angular resolution of the *Fermi*-LAT data, the southern gamma-ray emission remains spatially correlated with the eROSITA X-ray shell, indicating that electron escape does not significantly affect the adopted spatial modeling.

Due to the lack of multiwavelength data on the G189N component, we focus on the constraints imposed by the gamma-ray spectrum derived in this work. The density of the medium within the S249 HII region amounts to *n*_0_ = 50 cm^−3^ to 2000 cm^−3^ as reported by ref. ^16^. We used the most conservative value of 50 cm^−3^, fixed the particle injection index at 2.0 and the electron-proton ratio at 0.01, as done above for the G189S component to reproduce the gamma-ray data. The steepness of the gamma-ray spectrum above a few GeV implies a low energy cut-off value of 100 GeV as clearly demonstrated in Fig. 6 (left panel). Alternatively, the gamma-ray data can be reproduced using a steeper injection spectrum of 2.3 and a higher energy cutoff at 0.6 TeV (middle panel). This low energy cutoff with respect to the southern component can be due to the recent interaction of the supernova remnant with the dense material of the S249 HII region, which also strongly influences the energetics and radiative properties of the system.

Within the framework of these standard DSA scenarios, both spectral solutions require a comparable energy injected into relativistic protons and, given the broad uncertainty in the ambient gas density (*n*_0_) discussed above, the latter can be expressed in a scalable form as

8 $${W}_{p}\simeq 1.9\times 1{0}^{48}\left(\frac{50\,{{{{\rm{cm}}}}}^{-3}}{{n}_{0}}\right){{{\rm{erg}}}}\quad \left({{{\rm{equivalently}}}}\,{W}_{p}\simeq 9.5\times 1{0}^{49}\left(\frac{1\,{{{{\rm{cm}}}}}^{-3}}{{n}_{0}}\right){{{\rm{erg}}}}\right).$$

Although the gamma-ray spectrum in the northern region can be reproduced using proton spectra compatible with standard diffusive shock acceleration, the dense environment and inferred interaction with the S249 HII region imply strongly decelerated and radiative shock conditions. Under such conditions, fresh particle acceleration to high energies is expected to be inefficient, and the observed low cutoff energy is naturally explained by compression and re-acceleration of pre-existing cosmic rays in the dense post-shock gas. As such, alternative acceleration mechanisms associated with dense gas, and capable of reproducing the observed emission in radiative regions, are explored in subsection ‘Proton re-acceleration’.

The dense environment also strongly constrains alternative leptonic interpretations of the northern gamma-ray emission. Reproducing the observed gamma-ray flux through a Bremsstrahlung-dominated scenario would require an electron-to-proton ratio approaching unity, significantly larger than typically inferred for Galactic cosmic rays and SNRs. Likewise, reproducing the observed gamma-ray emission through an IC-dominated scenario would require unrealistically low ambient densities and unusually high electron-to-proton ratios in order to suppress both the proton–proton interaction and Bremsstrahlung emission. Such conditions are difficult to reconcile with the dense environment of the S249 interaction region and with the strong spatial correlation between the gamma-ray emission, molecular material, and the prominent H*α* filament.

### Proton re-acceleration

The intensity variation observed between the northern (hadronic) gamma-ray blob, spatially coincident with the H*α* filament, and the leptonically induced gamma-ray emission from the remainder of the remnant’s area suggests a scenario of proton re-acceleration followed by contraction within the thin H*α* filament. To further investigate this scenario, we performed crushed-cloud modeling of the northern hadronic gamma-ray component, following the framework introduced by ref. ^94^ and subsequently developed in dedicated numerical studies (e.g.^95–97^), with the aim of assessing whether a re-acceleration scenario can reproduce the observed emission.

In particular, we suggest that the gamma-ray emission from the G189N component is the result of the shock reacceleration of the energetic nuclei that constitute the ubiquitous Galactic cosmic ray background, followed by their adiabatic compression in the radiative layer downstream of the shock. We assumed that the SNR shock is still evolving in the Sedov phase and first encountered the S249 cloud when its radius (age) was about 16 pc (13 kyr) and its velocity approximately 500 km/s. When the SNR shock hits the cloud, a strong and much slower (50 km/s) shock is driven into the dense gas. If the cloud has a density equal to 50 cm^−3^, the current value for the total mass of the gas shocked so far is  ≳ 300*M*_⊙_. After being reaccelerated at the shock and compressed downstream of it, cosmic rays interact with the very dense radiative layer and produce gamma-rays via hadronic interactions with the ambient gas. A comparison between the prediction of the model and data is shown on the right panel of Fig. 6, where we assumed that the maximum energy of particles accelerated at the shock is 150 GeV and that all reaccelerated particles remain confined downstream of the shock. This indicates that a simple crushed cloud model as the one developed in ref. ^94^ can provide a quite natural explanation of the gamma-ray emission from G189N.

To further support our claim, we searched for UV signatures from the area where the remnant interacts with the molecular material. In principle, UV emission serves as an effective indicator of the radiative shocks that might be present in interstellar clouds with high densities. Unlike non-radiative shocks, radiative shocks are slower and do not efficiently accelerate particles via the DSA mechanism. As such, the detection of prominent UV filaments from the region where the remnant interacts with dense material would further substantiate the model involving the re-acceleration of preexisting ambient cosmic rays.

No Galaxy Evolution Explorer (GALEX) observations of the remnant have been performed; however, we found three Swift UVOT observations of small portions of the northern part of the remnant (ObsID: 00038720001, 00084926003, 00084926004), one of which (00084926003) partially covers the prominent H*α* filament. In Supplementary Fig. 2, we present the Swift UVOT level 2 processing data (reduced and corrected as provided by the Barbara A. Mikulski Archive for Space Telescopes [MAST]) recorded with the U filter (central wavelength: 346.5 nm), which corresponds to the near-UV (UVA) energy range. The UV emission closely traces the morphology of the optical filament. When considered together with the strong optical forbidden-line emission and elevated S II to H*α* ratios measured in this region^16^, the UV detection provides independent and complementary evidence that the filament traces radiative, cooling post-shock gas. This multiwavelength consistency strengthens the interpretation that the northern boundary of the remnant, where it interacts with the S249 HII region, is dominated by radiative shocks, thereby supporting a scenario in which the observed gamma-ray emission arises from the re-acceleration of pre-existing cosmic rays rather than an interpretation linked to freshly accelerated particles.

If the gamma-ray emission from G189N is indeed associated with compression and re-acceleration of pre-existing cosmic rays within the radiative shock interacting with the S249 region, then enhanced radio synchrotron emission is also expected from the same interaction site, since the processes responsible for re-accelerating relativistic protons should likewise act on relativistic electrons, albeit potentially with different efficiencies and energy-dependent acceleration rates. In this context, our objective was also to report on the predicted radio synchrotron flux density from the radiative shock and to compare it with observational DRAO data at 1.4 GHz from the filament location. Our ultimate goal is to establish a reference radio synchrotron flux density value from G189N to facilitate future research endeavors. To this end, we have performed an aperture-based radio flux-density estimate for the filament region (G189N) using the CGPS/DRAO 1.42 GHz data. As expected in the re-acceleration scenario discussed above, the filamentary radio structure, confirmed to be non-thermal in nature by ref. ^10^, is clearly enhanced relative to both its immediate surroundings and the broader SNR emission (Supplementary Fig. 4), despite the inherent background uncertainty and potential confusion between thermal and non-thermal radio contributions from the S249 region, which may however, affect the precise determination of the discrepancy between the predicted and observed radio synchrotron flux density.

Given this localized radio enhancement, it becomes important to estimate the synchrotron flux density specifically associated with the filament region rather than with the remnant as a whole. In instances where an SNR exhibits a highly non-uniform distribution of radio emission, the total flux density may not accurately represent the flux density of particularly brighter regions. To quantify this, we utilize the DRAO-1.42 GHz map (as applied in the computation of radio flux density from the entire SNR) and focus our analysis on the northern bright radio boundary of the SNR (G189N). We select as on-source flux density extraction region the entire wide arc feature (comparable to the size of the G189N gamma-ray component) seen in the DRAO maps encompassing the H*α* filament, which is evidently even brighter. We employ two distinct background control regions in the vicinity of the SNR but well outside its extension. One is positioned in a region devoid of thermal emission from S249, while the second is placed within S249 to account for potential thermal emission contributions from the on-source region. Applying the Rayleigh-Jeans law we obtained a radio synchrotron flux density of 2.0 Jy (accounting for thermal contributions from S249 and/or apparent radio contributions that might be unrelated to S249 to the north of G189.6+3.3, as if G189N is embedded at the most enhanced radio emission regions detected across S249 area) and 6.3 Jy (considering no contributions from the S249 region).

We emphasize that extracting the radio synchrotron flux density from the entire remnant, based on the available observational data, presents significant challenges, primarily due to the background uncertainty of a large-area SNR, the overlap with IC 443 to the west, and the presence of a thermal radio component (S249) potentially overlapping with the northern regions of the SNR. It is also evident from the radio surveys (refer to Supplementary Fig. 4) that the radio emission from the full shell is significantly dimmer compared to the northern boundary of the remnant coincident with G189N and is close to, if not at, the background level. Thus, we conservatively adopt the lower end of the estimated flux-density range (2 Jy) when discussing the radio synchrotron emission associated with the remnant, while future radio observations with improved sensitivity, angular resolution, and multi-frequency coverage are expected to enable improved background characterization and a more robust disentanglement of thermal and non-thermal emission, even if the full extent of the remnant is not completely recovered.

Although these flux-density estimates provide a useful reference for comparison with the re-acceleration model, the radio spectral index could in principle serve as an additional diagnostic of whether the filament emission is consistent with a particle re-acceleration scenario. However, the currently available radio data do not permit a sufficiently robust determination. While radio spectral indices can often be robustly derived for bright, isolated filaments where background uncertainties largely cancel, this assumption does not hold for the faint filamentary emission discussed here. In this case, the structure is detected with varying significance across the available radio surveys, each characterized by different angular resolution, spatial filtering, and background properties. As a result, uncertainties in background subtraction do not cancel in spectral-index measurements and instead dominate the inferred slope. In addition, while a radio spectral index can formally be derived from two flux-density measurements, although additional measurements at multiple frequencies are strongly preferable, a robust determination requires sufficiently detected emission in matched-resolution datasets with well-controlled systematics.

In the present case, although the filament is clearly identifiable in the available radio surveys, the emission remains relatively faint and heterogeneous across sufficiently separated frequencies, making the inferred slope highly sensitive to systematic effects. In addition to the factor-of-three uncertainty associated with the CGPS background treatment, any corresponding estimate from the GB6 survey is expected to carry even larger uncertainties because of differences in angular resolution, spatial filtering, sensitivity to diffuse emission, and local background structure. Under these conditions, any derived spectral-index estimate would be dominated by systematic rather than statistical uncertainties and therefore would not provide a sufficiently robust physical diagnostic. We therefore refrain from overinterpreting any spectral-index estimate as a diagnostic for the re-acceleration scenario and instead focus on the consistency between the observed radio flux density (estimated above) and that predicted by the re-acceleration model, which provides a more robust assessment given the limitations of the current radio data.

To estimate the level of synchrotron emission expected under the radiative shock re-acceleration scenario, we adopted the crushed-cloud framework previously applied to evolved interacting SNRs. We applied pressure equilibrium to derive the radiative compression factor, *s* = (compressed cloud density/cloud density)/r_*sh*_, with the shock compression ratio *r*_*sh*_ = 4. For an upstream magnetic field value of 10 *μ*G in the cloud, a cloud density of 50 cm^−3^, and a 50 km/s shock propagating within the cloud, we adopted the methodology outlined in refs. ^41,94^ to compute the relevant physical parameters. This approach yielded a compressed density of 1662 cm^−3^ and a compressed magnetic field of 271*μ*G (refer to eq. 3, 4, and 5 in refs. ^41^). Consequently, the value of *s* is determined to be 8.31.

Using these compressed physical conditions, we estimated the expected synchrotron-to-*π*^0^-decay ratio by comparison with the Cygnus Loop, which represents a similarly evolved interacting SNR. Synchrotron emission scales with the magnetic field strength raised to the power of (1+*α*), where *α* represents the electron energy index (here *α*=0.5). In contrast, the *π*^0^ decay scales with ambient density. Given that the Cygnus Loop is a SNR with conditions similar to our case, we compute the ratios of the compressed density and magnetic field of the two remnants. Those are estimated to be 5.67 and 1.11, respectively. Therefore, at this first-order approximation, the ratio of synchrotron to *π*^0^ for G189.6+3.3 should be approximately 0.206 of that in the Cygnus Loop. Considering the predicted radio flux density (at 1.4 GHz) for the re-acceleration scenario of the Cygnus Loop^41^ and the gamma-ray flux density estimates in the 0.1-100 GeV energy range for both the Cygnus Loop^41^ and G189N, 1.89 ± 0.14 × 10^−5^ MeV cm^−2^ *s*^−1^ (or 3.03 ± 0.22 × 10^−11^ erg cm^−2^s^−1^) as derived in this work, we predict a synchrotron flux density at 1.4 GHz of 4.5 ± 0.6 Jy.

Consequently, the apparent radio synchrotron enhancement, as depicted in all radio maps of different frequencies in Supplementary Fig. 4, which is evidently spatially coincident with the thin filament, and the broadly consistent results between the observational radio flux-density estimate from the filament region and the predicted radio synchrotron flux density from the crushed-cloud model, further support the re-acceleration scenario.

## Supplementary information

Supplementary Information

Transparent Peer Review file

## Source data

Source Data

## Data Availability

*Fermi*-LAT Pass 8 data and associated analysis tools are publicly available through the Fermi Science Support Center (FSSC; https://fermi.gsfc.nasa.gov/ssc/). eROSITA eRASS1 data and analysis resources are publicly accessible through the Data Release 1 (DR1) site (https://erosita.mpe.mpg.de/dr1/) and the HEASARC eROSITA archive (https://heasarc.gsfc.nasa.gov/docs/srg/erosita/). Additional datasets used in the analysis, including WISE infrared survey data (https://irsa.ipac.caltech.edu/Missions/wise.html), CO Galactic Plane Survey data, MWISP DR1 data (MWISP DR1 data archive), GLEAM radio survey data (https://www.mwatelescope.org/science/galactic-science/gleam/), DSS2 optical images (https://archive.stsci.edu/dss/), Swift UVOT observations (https://heasarc.gsfc.nasa.gov/docs/swift/archive/), DRAO and GB6 radio survey data (https://skyview.gsfc.nasa.gov/), and EXPLORE G-Tomo extinction products (https://explore-platform.eu/sdas/about/gtomo), are publicly available through their respective survey archives and data centers.  Source data underlying figures containing plotted data are provided with this paper.

## Code availability

This research made use of the Python package fermipy for the *Fermi*-LAT analysis^71^; the eSASS software^75^, described in the appendices of that work, for the analysis of eRASS1 data. The software and documentation are publicly available at https://erosita.mpe.mpg.de/edr/DataAnalysis/ and https://heasarc.gsfc.nasa.gov/docs/srg/erosita/analysis/. We also used the *naima* package^93^ for multiwavelength spectral modeling, and the open source software suite COMPAS^48^, available at https://compas.science, for the population synthesis. The three-dimensional dust distributions were explored using the EXPLORE G-Tomo tool, which is publicly available at https://explore-platform.eu/sdas/about/gtomo. No proprietary or restricted software was used in this study. The analysis procedures are described in the Methods section, and no custom software package is required to reproduce the results.

## References

[1] Green, D. A. A revised catalogue of 294 Galactic supernova remnants. *J. Astrophys. Astron.* **40**, 36 (2019).

[2] Green, D. A. An updated catalogue of 310 Galactic supernova remnants and their statistical properties. *J. Astrophys. Astron.* **46**, 14 (2025).

[3] Anderson, L. D. et al. Supernova Remnant Candidates Discovered by the SARAO MeerKAT Galactic Plane Survey. arXiv e-prints arXiv:2409.16607 (2024).

[4] Michailidis, M., Pühlhofer, G., Santangelo, A., Becker, W. & Sasaki, M. X-ray counterpart detection and *γ*-ray analysis of the supernova remnant G279.0+01.1 with eROSITA and Fermi-LAT. *AA* **685**, A23 (2024).

[5] Michailidis, M., Pühlhofer, G., Santangelo, A., Sasaki, M. & Becker, W. A look at the high energy aspects of the supernova remnant G309.8+00.0 with eROSITA and Fermi-LAT. *AA* **689**, A281 (2024).

[6] Michailidis, M. et al. Study of X-ray emission from the S147 nebula with SRG/eROSITA: X-ray imaging, spectral characterization, and a multiwavelength picture. *AA* **689**, A277 (2024).

[7] Schaudel, D. et al. Slane, P. O. & Gaensler, B. M. (eds) Galactic SNR Candidates in the ROSAT All-Sky Survey. (edsSlane, P. O. &Gaensler, B. M.) *Neutron Stars in Supernova Remnants*, Vol. 271 of *Astronomical Society of the Pacific Conference Series*, 391 (2002).

[8] S. Collaboration, H. E. S. et al. A search for new supernova remnant shells in the Galactic plane with H.E.S.S. *AA* **612**, A8 (2018).

[9] Asaoka, I. & Aschenbach, B. An X-ray study of IC443 and the discovery of a new supernova remnant by ROSAT. *AA* **284**, 573–582 (1994).

[10] Leahy, D. A. 1420 and 408 MHz Continuum Observations of the IC 443/G189.6+3.3 Region. *AJ* **127**, 2277–2283 (2004).

[11] Yamauchi, S., Oya, M., Nobukawa, K. K. & Pannuti, T. G. Discovery of recombining plasma associated with the candidate supernova remnant G189.6+3.3 with Suzaku. *PASJ* **72**, 81 (2020).

[12] Camilloni, F. & Becker, W. G189.6+03.3: The first complete X-ray view provided by SRG/eROSITA. *AA* **680**, A83 (2023).

[13] Ackermann, M. et al. Search for Extended Sources in the Galactic Plane Using Six Years of Fermi-Large Area Telescope Pass 8 Data above 10 GeV. *ApJ* **843**, 139 (2017).

[14] Atwood, W. B. et al. The Large Area Telescope on the Fermi Gamma-Ray Space Telescope Mission. *ApJ* **697**, 1071–1102 (2009).

[15] Abdollahi, S. et al. Search for Extended GeV Sources in the Inner Galactic Plane. arXiv e-prints arXiv:2411.07162 (2024).

[16] Bakış, H., Paylı, G., Aktekin, E., Sano, H. & Sezer, A. Optical and H I observations of IC 443 and G189.6 + 3.3 in a complex environment. *MNRAS* **532**, 2570–2583 (2024).

[17] Ballet, J., Bruel, P., Burnett, T. H. Lott, B. The Fermi-LAT collaboration. Fermi large area telescope fourth source catalog data release 4 (4FGL-DR4). arXiv e-prints arXiv:2307.12546 (2023).

[18] Abdollahi, S. et al. Incremental fermi large area telescope fourth source catalog. *ApJS* **260**, 53 (2022).

[19] Aartsen, M. G. et al. Searches for extended and point-like neutrino sources with four years of IceCube data. *ApJ* **796**, 109 (2014).

[20] Aartsen, M. G. et al. Constraints on galactic neutrino emission with seven years of IceCube data. *ApJ* **849**, 67 (2017).

[21] Lallement, R., Vergely, J. L., Babusiaux, C. & Cox, N. L. J. Updated Gaia-2MASS 3D maps of Galactic interstellar dust. *AA* **661**, A147 (2022).

[22] Aharonian, F. et al. A detailed spectral and morphological study of the gamma-ray supernova remnant <ASTROBJ>RX J1713.7-3946</ASTROBJ> with HESS. *AA* **449**, 223–242 (2006).

[23] S. Collaboration, H. E. S. et al. H.E.S.S. observations of RX J1713.7-3946 with improved angular and spectral resolution: Evidence for gamma-ray emission extending beyond the X-ray emitting shell. *AA* **612**, A6 (2018).

[24] S. Collaboration, H. E. S. et al. Deeper H.E.S.S. observations of Vela Junior (RX J0852.0-4622): Morphology studies and resolved spectroscopy. *AA* **612**, A7 (2018).

[25] Stecker, F. W. Neutral-Pion Gamma Rays from the Galaxy and the Interstellar Gas Content. *ApJ* **185**, 499–504 (1973).

[26] Ackermann, M. et al. Detection of the characteristic pion-decay signature in supernova remnants. *Science* **339**, 807–811 (2013). 23413352 10.1126/science.1231160

[27] Jogler, T. & Funk, S. Revealing W51C as a cosmic ray source using fermi-LAT data. *ApJ* **816**, 100 (2016).

[28] Ambrogi, L. et al. Spectral and morphological study of the gamma radiation of the middle-aged supernova remnant HB 21. *AA* **623**, A86 (2019).

[29] de Oña Wilhelmi, E. et al. SNR G39.2-0.3, an hadronic cosmic rays accelerator. *MNRAS* **497**, 3581–3590 (2020).

[30] Abdollahi, S. et al. Search for New Cosmic-Ray Acceleration Sites within the 4FGL Catalog Galactic Plane Sources. *ApJ* **933**, 204 (2022).

[31] Xin, Y.-L. et al. Revisiting SNR Puppis A with Seven Years of Fermi Large Area Telescope Observations. *ApJ* **843**, 90 (2017).

[32] Giuffrida, R. et al. Evidence for proton acceleration and escape from the Puppis A SNR using Fermi-LAT observations. arXiv e-prints arXiv:2308.14848 (2023).

[33] Fukui, Y. et al. Pursuing the origin of the gamma rays in RX J1713.7-3946 quantifying the hadronic and leptonic components. *ApJ* **915**, 84 (2021).

[34] Sharma, P., Ou, Z., Henry-Cadrot, C., Dubos, C. & Suomijärvi, T. Multiwavelength analysis of galactic supernova remnants. *JCAP* **2023**, 027 (2023).

[35] Lemoine-Goumard, M., Acero, F., Ballet, J. & Miceli, M. Hadronic particle acceleration in the supernova remnant SN 1006 as traced by Fermi-LAT observations. arXiv e-prints arXiv:2412.08190 (2024).

[36] McLean, B. J., Greene, G. R., Lattanzi, M. G. & Pirenne, B., Manset, N., Veillet, C. & Crabtree, D. (eds) *The Status of the Second Generation Digitized Sky Survey and Guide Star Catalog*. (eds Manset, N.,Veillet, C. & Crabtree, D.) *Astronomical Data Analysis Software and Systems IX*, Vol.216 of *Astronomical Society of the Pacific Conference Series*, 145 (2000).

[37] Fesen, R. A. The nature of the filaments northeast of the supernova remnant IC 443. *ApJ* **281**, 658–664 (1984).

[38] Lee, J.-J. et al. A 21 cm spectral and continuum study of IC 443 using the very large array and the arecibo telescope. *AJ* **135**, 796–808 (2008).

[39] Bell, A. R. The acceleration of cosmic rays in shock fronts - I. *MNRAS* **182**, 147–156 (1978).

[40] Blandford, R. & Eichler, D. Particle acceleration at astrophysical shocks: A theory of cosmic ray origin. *Phys. Rep.* **154**, 1–75 (1987).

[41] Tutone, A., Ballet, J., Acero, F., D’Aì, A. & Cusumano, G. Multiple accelerated particle populations in the Cygnus Loop with Fermi-LAT. *AA* **656**, A139 (2021).

[42] Dame, T. M., Hartmann, D. & Thaddeus, P. The milky way in molecular clouds: A new complete CO survey. *ApJ* **547**, 792–813 (2001).

[43] Yang, J. et al. The milky way imaging scroll painting survey: Data release 1. arXiv e-prints arXiv:2512.08260 (2025).

[44] Burton, M. IC 443 : the interaction of a supernova remnant with a molecular cloud. *Q. J. R. Astron. Soc.* **28**, 269–276 (1987).

[45] Zhang, Z., Gao, Y. & Wang, J. CO observation of SNR IC 443. *Sci. China Phys., Mech., Astron.* **53**, 1357–1369 (2010).

[46] Egron, E. et al. Imaging of SNR IC443 and W44 with the Sardinia Radio Telescope at 1.5 and 7 GHz. *MNRAS* **470**, 1329–1341 (2017).

[47] Ustamujic, S. et al. Modeling the mixed-morphology supernova remnant IC 443. Origins of its complex morphology and X-ray emission. *AA* **649**, A14 (2021).

[48] Riley, J. et al. Rapid stellar and binary population synthesis with COMPAS. *ApJS* **258**, 34 (2022).

[49] Asplund, M., Grevesse, N., Sauval, A. J. & Scott, P. The chemical composition of the sun. *ARAA* **47**, 481–522 (2009).

[50] Szécsi, D. et al. Low-metallicity massive single stars with rotation. Evolutionary models applicable to I Zwicky 18. *AA* **581**, A15 (2015).

[51] Greco, E. et al. Discovery of a jet-like structure with overionized plasma in the SNR IC 443. *AA* **615**, A157 (2018).

[52] Smartt, S. J. Progenitors of Core-Collapse Supernovae. *ARAA* **47**, 63–106 (2009).

[53] Ustamujic, S. et al. Modeling the remnants of core-collapse supernovae from luminous blue variable stars. *AA* **654**, A167 (2021).

[54] Troja, E., Bocchino, F., Miceli, M. & Reale, F. XMM-Newton observations of the supernova remnant IC 443. II. Evidence of stellar ejecta in the inner regions. *AA* **485**, 777–785 (2008).

[55] Katsuda, S., Takiwaki, T., Tominaga, N., Moriya, T. J. & Nakamura, K. Progenitor Mass Distribution of Core-collapse Supernova Remnants in Our Galaxy and Magellanic Clouds Based on Elemental Abundances. *ApJ* **863**, 127 (2018).

[56] Woosley, S. E. & Weaver, T. A. The Evolution and Explosion of Massive Stars. II. Explosive Hydrodynamics and Nucleosynthesis. *ApJS* **101**, 181 (1995).

[57] Boffin, H. M. J. & Jones, D. The importance of binary stars. *Contributions Astronomical Observatory Skalnate Pleso* **55**, 21–36 (2025).

[58] Li, Z., Wheeler, J. C., Bash, F. N. & Jefferys, W. H. A statistical study of the correlation of galactic supernova remnants and spiral arms. *ApJ* **378**, 93 (1991).

[59] Tammann, G. A., Loeffler, W. & Schroeder, A. The galactic supernova rate. *ApJS* **92**, 487 (1994).

[60] Anderson, L. D. et al. Galactic supernova remnant candidates discovered by THOR. *AA* **605**, A58 (2017).

[61] Wang, C. et al. Molecular gas toward the gemini OB1 molecular cloud complex. I. Observation data. *ApJS* **230**, 5 (2017).

[62] Foster, T. J., Cooper, B., Reich, W., Kothes, R. & West, J. Two radio supernova remnants discovered in the outer Galaxy. *AA* **549**, A107 (2013).

[63] Albert, J. et al. Discovery of very high energy gamma radiation from IC 443 with the MAGIC telescope. *ApJL* **664**, L87–L90 (2007).

[64] Torres, D. F., Rodriguez Marrero, A. Y. & de Cea Del Pozo, E. MAGIC J0616+225 as delayed TeV emission of cosmic rays diffusing from the supernova remnant IC 443. *MNRAS* **387**, L59–L63 (2008).

[65] Acciari, V. A. et al. Observation of Extended Very High Energy Emission from the Supernova Remnant IC 443 with VERITAS. *ApJL* **698**, L133–L137 (2009).

[66] Mitchell, A. M. W., Grosspietsch, L. & Wach, T. Analysis of the supernova remnant IC 443 using H.E.S.S. Data. arXiv e-prints arXiv:2510.02843 (2025).

[67] Alfaro, R. et al. Study of the IC 443 region with the HAWC observatory. *ApJ* **992**, 22 (2025).

[68] Cao, Z. et al. Evidence of cosmic-ray acceleration up to sub-PeV energies in the supernova remnant IC 443. arXiv e-prints arXiv:2510.26112 (2025). 10.1103/pxn6-qzhz 42113166

[69] Atwood, W. et al. Pass 8: Toward the full realization of the fermi-LAT scientific potential. arXiv e-prints arXiv:1303.3514 (2013).

[70] Bruel, P. et al. Fermi-LAT improved Pass~8 event selection. arXiv e-prints arXiv:1810.11394 (2018).

[71] Wood, M. et al. Fermipy: An open-source Python package for analysis of Fermi-LAT Data. *PoS* **ICRC2017**, 824 (2017).

[72] Merloni, A. et al. eROSITA Science Book: Mapping the structure of the energetic universe. arXiv e-prints arXiv:1209.3114 (2012).

[73] Predehl, P. et al. The eROSITA X-ray telescope on SRG. *AA* **647**, A1 (2021).

[74] Sunyaev, R. et al. SRG X-ray orbital observatory. Its telescopes and first scientific results. *AA* **656**, A132 (2021).

[75] Brunner, H. et al. The eROSITA final equatorial depth survey (eFEDS). X-ray catalogue. *AA* **661**, A1 (2022).

[76] Bruel, P. A new method to perform data-model comparison in Fermi-LAT analysis. *AA* **656**, A81 (2021).

[77] Akaike, H. A new look at the statistical model identification. *IEEE Trans. Autom. Control* **19**, 716–723 (1974).

[78] Acero, F. et al. The first fermi LAT supernova remnant catalog. *ApJS* **224**, 8 (2016).

[79] Case, G. L. & Bhattacharya, D. A new Σ-D relation and its application to the galactic supernova remnant distribution. *ApJ* **504**, 761–772 (1998).

[80] Badenes, C., Maoz, D. & Draine, B. T. On the size distribution of supernova remnants in the magellanic clouds. *MNRAS* **407**, 1301–1313 (2010).

[81] Foight, D. R., Güver, T., Özel, F. & Slane, P. O. Probing X-ray absorption and optical extinction in the interstellar medium using chandra observations of supernova remnants. *ApJ* **826**, 66 (2016).

[82] Ambrocio-Cruz, P., Rosado, M., de la Fuente, E., Silva, R. & Blanco-Piñon, A. Kinematic study at the H *α* line in the north-eastern region of the Galactic supernova remnant IC 443. *MNRAS* **472**, 51–54 (2017).

[83] Zhao, H. et al. A systematic study of the dust of galactic supernova remnants. I. The distance and the extinction. *ApJ* **891**, 137 (2020).

[84] Dell’Ova, P. et al. Interstellar anatomy of the TeV gamma-ray peak in the IC443 supernova remnant. *AA* **644**, A64 (2020).

[85] Yamaguchi, H. et al. Discovery of strong radiative recombination continua from the supernova remnant IC 443 with Suzaku. *ApJL* **705**, L6–L9 (2009).

[86] Chevalier, R. A. The evolution of supernova remnants. Spherically symmetric models. *ApJ* **188**, 501–516 (1974).

[87] Leahy, D. A. & Williams, J. E. A python calculator for supernova remnant evolution. *AJ* **153**, 239 (2017).

[88] Devin, J. et al. High-energy gamma-ray study of the dynamically young SNR G150.3+4.5. *AA* **643**, A28 (2020).

[89] Petre, R., Szymkowiak, A. E., Seward, F. D. & Willingale, R. A comprehensive study of the X-ray structure and spectrum of IC 443. *ApJ* **335**, 215 (1988).

[90] Chevalier, R. A. Supernova remnants in molecular clouds. *ApJ* **511**, 798–811 (1999).

[91] Olbert, C. M., Clearfield, C. R., Williams, N. E., Keohane, J. W. & Frail, D. A. A bow shock nebula around a compact X-ray source in the supernova remnant IC 443. *ApJL* **554**, L205–L208 (2001).

[92] Tubín-Arenas, D. et al. The eROSITA upper limits. Description and access to the data. *AA* **682**, A35 (2024).

[93] Zabalza, V. Naima: a Python package for inference of particle distribution properties from nonthermal spectra. *PoS* **ICRC2015**, 922 (2016).

[94] Uchiyama, Y., Blandford, R. D., Funk, S., Tajima, H. & Tanaka, T. Gamma-ray emission from crushed clouds in supernova remnants. *ApJL* **723**, L122–L126 (2010).

[95] Tang, X. & Chevalier, R. A. Gamma-ray emission from supernova remnant interactions with molecular clumps. *ApJL* **784**, L35 (2014).

[96] Lee, S.-H. et al. Modeling bright *γ*-ray and radio emission at fast cloud shocks. *ApJ* **806**, 71 (2015).

[97] Cardillo, M., Amato, E. & Blasi, P. Supernova remnant W44: A case of cosmic-ray reacceleration. *AA* **595**, A58 (2016).

[98] Wright, E. L. et al. The wide-field infrared survey explorer (WISE): Mission description and initial on-orbit performance. *AJ* **140**, 1868–1881 (2010).

