# Supplementary material for: Shared cloud interactions unveil a candidate binary-system supernova pair with no known analogue
Source: Nat Commun. 2026 Jul 21;17:6190. doi: 10.1038/s41467-026-74978-x (PMC13389397; doi:10.1038/s41467-026-74978-x)
Supplement: Supplementary file 1 — Supplementary Information [file 41467_2026_74978_MOESM1_ESM.pdf]

Shared Cloud Interactions Unveil a Binary-System Supernova Pair with No Known Analogue

Supplementary Material

Miltiadis Michailidis[1,2,3], Marianne Lemoine-Goumard[4], Reinhold Willcox[5], Stefano Gabici[6], Niccolo Di Lalla[1,2,3], Nicola Omodei[1,2,3]

[1]W. W. Hansen Experimental Physics Laboratory, Stanford, CA 94305, USA
[2]Kavli Institute for Particle Astrophysics and Cosmology, Stanford, CA 94305, USA
[3]Department of Physics and SLAC National Accelerator Laboratory, Stanford University, Stanford, CA 94025, USA
[4]Univ. Bordeaux, CNRS, LP2i Bordeaux, UMR 5797, F-33170 Gradignan, France
[5]Institute of Astronomy, KU Leuven, Celestijnenlaan 200D, 3001 Leuven, Belgium
[6]Université Paris Cité, CNRS, Astroparticule et Cosmologie, F-75013 Paris, France

Corresponding author: milmicha@stanford.edu

Supplementary Table 1: SED modeling parameters.

Component	B	Γ	n_0	$E_{\mathrm{max,e}}$	$E_{\mathrm{max,p}}$	W_p	K_ep
	μG		cm^{-3}	TeV	TeV	erg	
G189S	5	2	0.5	5	5	10^{49}	0.01
G189N	271	2.0	50	0.1	0.1	1.9×10^{48}	0.01
		2.3	50	0.6	0.6	1.9×10^{48}	0.01

Energy budget parameters obtained from the multiwavelength SED modeling of G189S and G189N. Column 2 gives the magnetic field strength. The particle injection index is fixed at 2; for G189N we also adopt a value of 2.3 (Column 3). Column 4 lists the ambient ISM density. Columns 5 and 6 report the maximum energies of electrons and protons, respectively. Column 7 gives the total energy injected into protons, and Column 8 the electron-to-proton ratio. All parameters are either inputs to, or constrained by, the SED modeling and represent representative values selected for physical consistency and model stability. An exception is the magnetic field strength for the G189N hadronic component, which is derived separately in methods, subsection 'Proton re-acceleration', in the context of the proton re-acceleration scenario.

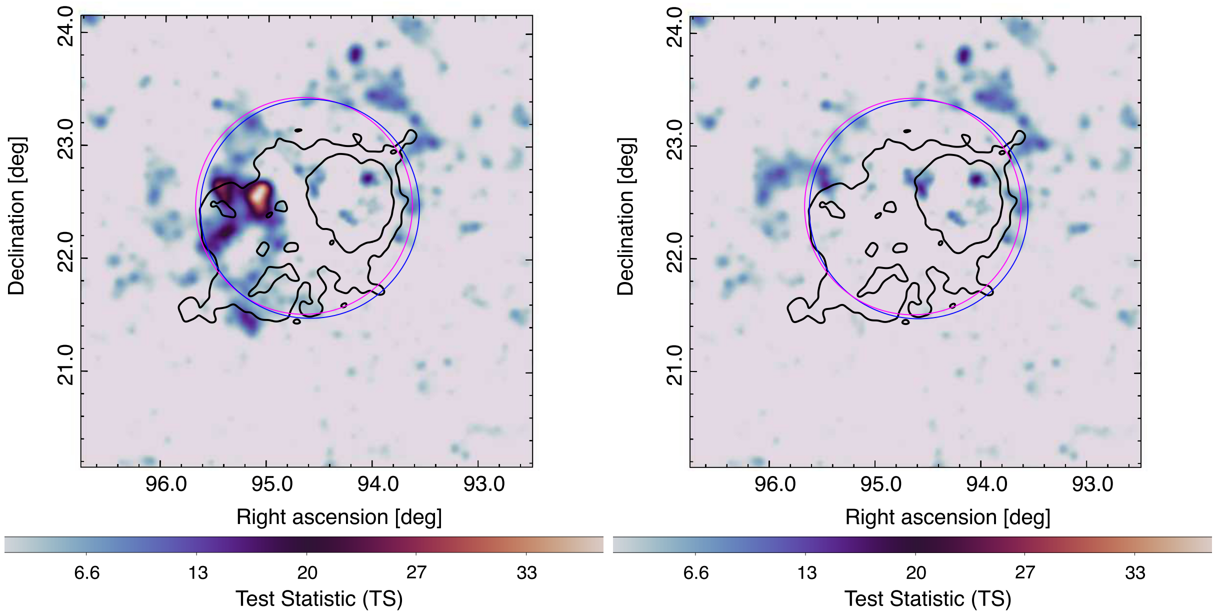

Supplementary Fig. 1: *Fermi*-LAT residual TS maps. Left panel: *Fermi*-LAT TS map > 1 GeV after adding to our model all four Fermi point sources from the best spatial model prior to this work. Right panel: *Fermi*-LAT TS map > 1 GeV after adding to our model the best fit spatial template (spatial template 13 in the main text Tab. 1) as obtained from the morphological analysis performed in this work. In both panels, the magenta and blue circles represent position and extension of FGES J0619.6+2229 [1] and 2FGES J0618.3+2227 [2], respectively. Black contours represent X-ray extension of both G189.6+3.3 and IC 443 as seen with eROSITA (contours are extracted from the main text Fig. 2, left panel). The latter is embedded within the extension of G189.6+3.3.

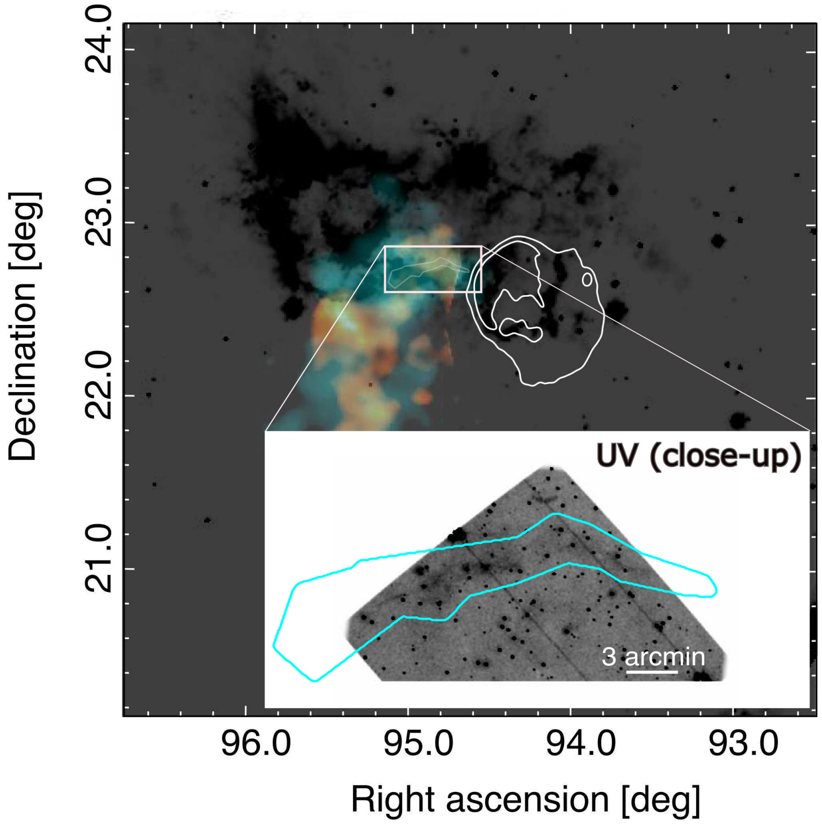

Supplementary Fig. 2: UV emission from the interaction region. Swift UVOT observation (ID: 00084926003) shown as an inset on the main text Fig. 1, highlighting a small region towards the northern boundary of the SNR G189.6+3.3 interacting with the S249 HII region. The U filter of Swift UVOT was used for the observation. The cyan contour highlights the Hα filament location as extracted from the left panel of the main text Fig. 8. The inset provides a zoomed-in view, while the underlying image supplies the larger-scale context and indicates the location of the UV-emitting region within the IC 443 complex.

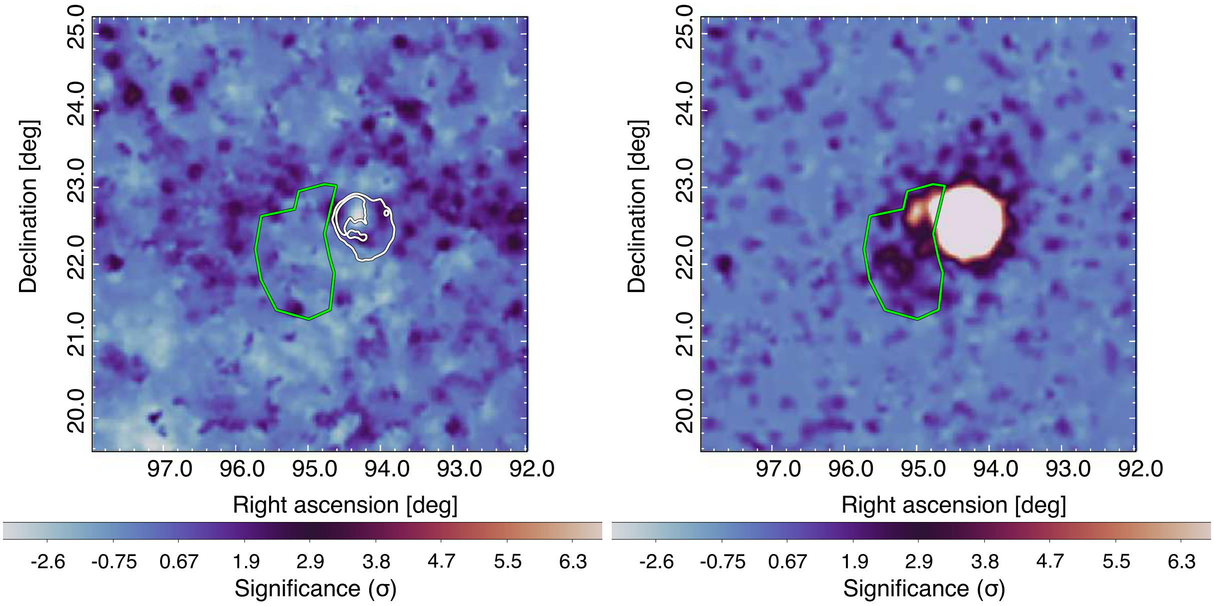

Supplementary Fig. 3: $5.7^\circ \times 5.7^\circ$ *Fermi*-LAT PS maps in the IC 443 complex. Left panel: *Fermi*-LAT PS map, in sigma, > 1 GeV, produced adopting the best fit spatial template (spatial template 13 in the main text Tab. 1) as obtained from the morphological analysis performed in this work. The white contours illustrate the radio continuum extent of IC 443 SNR as detected in GLEAM radio survey data. Right panel: *Fermi*-LAT PS map, in sigma, > 10 GeV. The *Fermi*-LAT map was produced after removing the four 4FGL-DR4 point sources used to account for the gamma-ray emission from G189.6+3.3 and the two existing 4FGL-DR4 point sources used to describe emission from the IC 443 region, namely 4FGL J0617.2+2234e and 4FGL J0616.5+2235. The IC 443 gamma-ray source appears saturated in the map. The green contour, in both panels, shows the polygonal region within which X-ray emission from G189.6+3.3 has been traced with eROSITA. The latter contour was extracted from the right panel of the main text Fig. 2 and used as spatial template for the gamma-ray morphological analysis in methods, subsection 'Morphological analysis' (corresponding to the first component of best-fit model 13 in the main text Tab. 1).

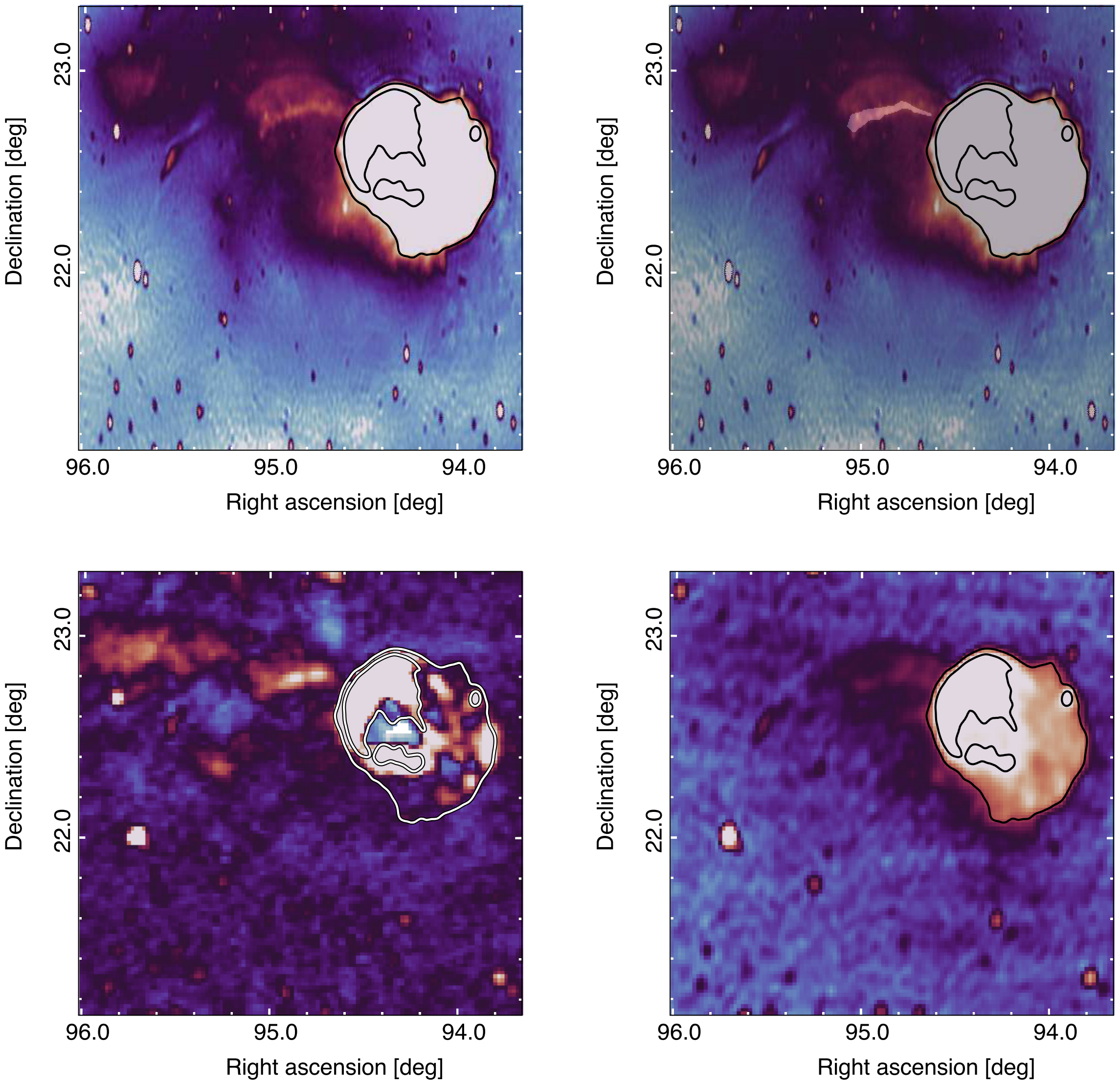

Supplementary Fig. 4: Radio view of the IC 443 complex. Upper panels: DRAO-1.42 GHz surface brightness radio map (Kelvin). In the right panel, the position of the Hα filament (re-acceleration site) is overlaid in white to highlight its spatial correspondence with a localized increase in radio synchrotron emission. Lower left panel: Green Bank 6-cm Survey (GB6)-4.85 GHz intensity radio map (Jy/beam). Lower right panel: GLEAM-0.155 GHz intensity radio map (Jy/beam). The black contours (white on the lower left panel) illustrate the radio continuum extent of IC 443 SNR as detected in GLEAM-0.155 GHz radio survey data.

References

[1] Ackermann, M. *et al.* Search for Extended Sources in the Galactic Plane Using Six Years of Fermi-Large Area Telescope Pass 8 Data above 10 GeV. *ApJ* **843**, 139 (2017). 1702.00476.

[2] Abdollahi, S. *et al.* Search for Extended GeV Sources in the Inner Galactic Plane. *arXiv e-prints* arXiv:2411.07162 (2024). 2411.07162.
