# Supplementary material for: Shared cloud interactions unveil a candidate binary-system supernova pair with no known analogue
Source: Nat Commun. 2026 Jul 21;17:6190. doi: 10.1038/s41467-026-74978-x (PMC13389397; doi:10.1038/s41467-026-74978-x)
Supplement: Supplementary file 2 — Transparent Peer Review file [file 41467_2026_74978_MOESM2_ESM.pdf]

nature portfolio

Peer Review File

Shared Cloud Interactions Unveil a candidate Binary-System Supernova Pair with No Known Analogue

Corresponding Author: Dr Miltiadis Michailidis

This file contains all reviewer reports in order by version, followed by all author rebuttals in order by version.

Version 0:

Reviewer comments:

Reviewer #1

(Remarks to the Author)
I followed the prompts listed in the instructions to reviewers page.

==
Key results

This work presents three key claims:

SNR G189.6+3.3 shows gamma-ray emission of leptonic and hadronic origin in two distinct regions.
The progenitors of SNR G189.6+3.3 and IC443 formed a binary system (most likely of massive stars, possibly including an LBV) with explosions happening "shortly" (<100 kyr) after each other.
The emission from a particular region of the source (referred to as “the H\alpha filament”) is compatible with a proton re-acceleration scenario.

The evidence presented is (a) an original analysis of 16 years of Fermi-LAT data (b) a compilation of archival values and a MW SED modeling and (c) some crushed cloud modeling of the hadronic component. Claim 2 is speculative and only supported circumstantially by the data.

==
Validity:

I did not find any flaws that should prohibit the manuscript’s publication.

==
Originality and significance:

The conclusions are original, and I think would be interesting to people in the fields of CR physics, Galactic astronomy, SNR evolution and feedback, and stellar populations.

==
Data & methodology:

The presentation of the data in the text and images is sometimes confusing. I would have appreciated a better display and labeling of the features discussed in the text in a large three-color image (e.g. DRAO, WISE, eROSITTA, or radio, mm, IR) to have a better sense, by eye, of the thermal/non-thermal components, the areas of overlap, and the general structure of the regions and how they interact with each other.

By “confusing" I mean things like: in the caption to Fig. 1 it says: “spatial comparison of X-ray, gamma-ray, and CO data,” but then there is no display of the CO data, just a mention that the authors have looked at it. Some of the datasets referenced are outdated (e.g. for CO they reference the Dame survey, whereas the higher resolution MWISP is available for this area). Fig. 4 has no coordinates. In this sense, the presentation is sloppy; these are some examples.

A lot of attention is paid to “the H\alpha filament.” In line 989 the authors state that the radio emission in the H\alpha filament is enhanced. I downloaded the GLEAM and CGPS 1.4 GHz maps and I can’t see any enhancement (although this is tricky since Fig. 4 does not have axis labels). If I am wrong about the location of the filament and the radio emission is indeed enhanced, and visible in the different radio maps, it would be nice to comment on its spectral index (estimating this for a thin filamentary structure does not present the challenges outlined in p. 22, which are more to do with measuring the flux **density** (anything measured in Jy is a flux density —this is another recurrent mistake in the manuscript) of an extended source. Is the radio spectral index consistent with a reacceleration scenario?

==
Appropriate use of statistics and treatment of uncertainties:
I think the statistics are handled correctly.

==
Conclusions:
The results are interesting; finding a source that shows both leptonic and hadronic emission will certainly provoke a lot of analysis in the field, and as the authors note will be a prime target for neutrino observations. It is a bit difficult for me to comment on the soundness of claim #1, since I am not an expert on Fermi data analysis, which underpins the result. As per claims #2 and #3, I think that they are both plausible. I find that the most solid piece of evidence that IC443 and SNR G198.6+3.3 are related (much more so than the distance/age estimates, which have large uncertainties) comes from the fact that they are both interacting with the same HII region, as the authors show here. Going from there to a claim that they used to be part of a binary system seems a bit of a stretch, and some of the claims that the authors make in the discussion (e.g. "Such results can, for the first time, revolutionize our understanding of stellar life cycles. They are also crucial for constraining models of stellar evolution, star formation, and even testing gravitational theories”) see overblown, reading more like a grant application than a paper.

==
Suggested improvements:

Perhaps the authors have already done this and decided against showing the image because it might not be very informative, so I am happy to defer to their judgement, but I would have liked to see that indeed the CO emission is spatially coincident with the hadronic gamma-ray emission. Could the authors comment on what kind of molecular observations could unambiguously confirm the association between the CO and the gamma-rays? Although I agree with the authors that the spatial coincidence is very suggestive, a chance alignment cannot be ruled out —spectral evidence (e.g. from the line ratios of certain components, or the emission of certain tracers) is necessary, in my opinion, to confirm this proposed scenario. I would urge the authors to use MWISP, but even if they only use the Dame data —what is the velocity of the associated cloud components? Does it match (using a rotation curve) the distance of the SNRs?

Please, have a figure that labels the characters in this paper, and particularly their extent (IC443, G189, HII reg S249, the H\alpha filament). In the case of zooms in of particular areas (e.g. Fig 6) it would help to show the inlet in a larger figure (although since Fig. 6 does actually have coordinates this is not as crucial).

Please give a thorough read to all captions and make sure that what is discussed in the caption is actually (a) relevant to the figure and (b) not first explained in a caption (e.g. the caption of Fig. 6 is the first time that the EXPLORE G-Tomo tool is mentioned —I would have appreciated a discussion in the text of how this tool works, and whether it is indeed suitable for an extended source, since my eyebrows were raised by the tiny uncertainty reported in the distance estimate from the optical extinction).

==
References:

In light of all the attention that is paid to VHE instruments in recent months, I am missing a comment on the LHAASO upper limits for this source. The E_max values mentioned in line 889 are within the WDCA. Fig. 11 suggests already that perhaps the LHAASO UL are not super useful, but not mentioning other gamma-ray observations of the area seems like a missed opportunity.

==
Clarity and context:

The abstract and introduction are clear, there is no conclusions section. As I mentioned above, some of the claims in the discussion are far-fetched.

Reviewer #2

(Remarks to the Author)
The manuscript presents the detection of extended GeV emission from a region spatially coincident with SNR G189.6+3.3 (hereafter referred to as G189), utilizing 16 years of Fermi-LAT data. The manuscript claims that the morphological and positional analyses of this GeV emission are consistent with the X-ray shell of the SNR G189.6 as observed by eROSITA. The manuscript further uses eROSITA, Swift UVOT data, and multi-wavelength SED modeling to suggest that in the

northern part, gamma rays are produced from the hadronic interaction between protons reaccelerated within the H-alpha filament via adiabatic contraction, with the cold protons in the nearby S249 HII cloud, whereas in the southern region, gamma rays are produced via the leptonic inverse-Compton channel. The manuscript further proposes that IC 443 and G189 together are the first observationally supported binary supernova systems, as determined through various analyses for age and distance estimations, including binary population synthesis using the COMPAS code.

The topic is highly interesting, the analysis is ambitious, and the potential implications, binary supernova remnants and clear hadronic/leptonic spatial separation, would be significant in the field of high-energy astrophysical systems if robustly demonstrated. The manuscript contains extensive observational and theoretical analysis. However, several key claims lack sufficient quantitative demonstration, and methodological clarity needs improvement before the results can be considered for publication. Therefore, I recommend the manuscript for publication in Nature Communications only after the suggested revisions have been made. I outline my major concerns below.

General comments/suggestions:

1. The most significant claim that IC 443 and G189 form the first observed binary supernova system needs to be sufficiently supported.

The distance estimates for both remnants rely on extinction-based arguments with non-negligible uncertainties (worst case scenario being around 500 pc, which seems very large if it is really a binary system). Furthermore, the overlap of gamma ray data with the S249 HII region does not by itself guarantee identical distances between G189 and IC443. I would suggest that the authors provide additional evidence, if possible, to confirm that the distances of the two SNRs are compatible with each other.

The manuscript presents the age estimates for G189 (30–100 kyr) and IC443 (3-30 kyr). These age ranges seem too broad to meaningfully constrain a time delay between explosions. Within such large ranges, nearly any sequence of explosion times is possible. A robust binary interpretation should require an explicit quantitative demonstration of the likelihood of the given delay from binary evolution models.
To that end, the authors do actually perform the binary population synthesis using the COMPAS code as supporting evidence, from which the time delay vs separation of SNRs predicted by the population synthesis of one million binaries are shown for two metallicities. Although interesting, it lacks quantitative estimation or a statistical test (even if it is implied, it is not presently clear), for example, the probability of obtaining the posited separation or delay time under a binary scenario versus a chance possibility. Without a statistical comparison, it is hard to substantiate a binary claim, even for future observations to look for.

Also, it is not clear to me, at least from the text itself, how a separation of 9 to 15 pc was posited? Can the authors make it clearer? Also, to this point, see point 4 below.

To summarize, the manuscript treats compatible distances and ages as proof of binary ancestry, but a statistical estimate needs to be shown for confirmation that this is not a chance spatial coincidence along the line of sight in an already complex region.

2. The multiwavelength modeling is presented well; however, the manuscript would benefit from including the key formulas used to derive the principal model parameters, such as the electron spectral break, maximum electron/proton energies, and related quantities, rather than only stating them briefly and citing external references. Providing these expressions directly in the text would make the analysis more transparent, easier to follow, reproducible, and more self-consistent for the reader.

The authors should show the interaction timescales for different interactions (pp, bremsstrahlung, synchrotron, IC, etc.), along with necessary dynamical timescales (for example, the system age, diffusion timescale, if relevant) for both regions, and discuss how that supports the separate emission from two zones.

The manuscript does not address the possibility of very-high-energy (VHE) gamma-ray detection in the >TeV range. If relevant data exist, the authors should include TeV-band overlays (e.g., from HAWC, MAGIC, VERITAS, or LHAASO WCDA) on their morphological figures to illustrate whether the GeV and TeV structures are spatially consistent. If not, the authors should discuss what future or current TeV instruments, such as CTAO, SWGO, and the LHAASO WCDA, are expected to observe from this region, if any, and whether such observations would serve the interpretations put forward in the manuscript.

Neutrino detection would provide definitive evidence for a hadronic origin of the gamma-ray emission from the northern region, as the manuscript correctly notes. Could the authors estimate the corresponding neutrino flux predicted by their hadronic model and compare it with the sensitivities of current and upcoming neutrino observatories? Such a comparison would help assess whether neutrino detection from this region is feasible and would further strengthen or challenge the proposed interpretation.

3. The proposed explanation that the northern gamma-ray component arises from proton re-acceleration within the H-alpha filament is interesting, yet somewhat incomplete in its current form. The manuscript invokes adiabatic compression and cosmic-ray re-acceleration, yet no physical modeling of compression diagnostics, possible proton spectral modifications, or cosmic-ray enhancement and gamma-ray luminosities is clearly presented.

The authors should also discuss the potential impact of ion–neutral damping and non-Bohmian diffusion within the cloud. These effects can significantly influence the overall gamma-ray emissivity and should therefore be addressed in the analysis.

Moreover, A simpler and well-established alternative of enhanced hadronic emission from shock-accelerated protons, which escape the shock front and interact with dense gas, remains entirely plausible. In fact, the hadronic modeling provided (at least seen from Table 3 and Figure 11) is more akin to DSA, rather than that of proton reacceleration via adiabatic contraction. Given that proton re-acceleration is an important claim of the manuscript, the authors should provide some diagnostics as to why it is preferred over other acceleration mechanisms, accompanied by necessary quantitative modeling.

4. The manuscript is extremely long, verbose, and difficult to navigate. The manuscript repeatedly re-states similar arguments in multiple sections. The narrative would benefit from substantial condensation and clearer presentation of core findings and necessary quantitative estimations. It would be advisable to soften the core claims and avoid overstating the conclusions. Moreover, the authors should discuss the caveats of their approach in a dedicated section, and they should also comment on the specific observational and theoretical developments that could help confirm, refine, or challenge their interpretation in the future.

Minor comments/questions:

1. The eROSITA observations indicate that the X-ray shell is dominated by thermal emission. However, the modeling yields a magnetic field of ~270 microG in the northern region. Could the authors comment on whether any non-thermal X-ray synchrotron component is expected under such a strong field, and how does that compare with the thermal X-ray flux inferred from the eROSITA data?

2. The derived magnetic field in the southern region (~5 microG) is close to the Galactic average. Such a low field implies long synchrotron cooling times and may allow high-energy electrons to escape efficiently from the emission zone. Could the authors discuss if and how electron escape is expected to change their spatial modeling of the southern region?

3. For the southern region, what mechanism is assumed for proton acceleration? Is it standard diffusive shock acceleration at the SNR forward shock, and if so, why not use the same in the northern region? To reiterate, clarifying the physical distinction between the two acceleration mechanisms in two separate environments for the same species would strengthen the interpretation.

4. The northern gamma-ray emission is modeled using a 2D Gaussian template, while the southern region is described using the eROSITA-based polygon corresponding to the full SNR shell. Could the authors clarify the physical and geometric motivation for this transition? For example, is the Gaussian intended to represent the molecular cloud interacting with the shock, while the polygon traces the broader SNR structure? Explicitly defining where the two components separate spatially would improve the clarity of the morphological analysis.

Reviewer #3

(Remarks to the Author)
Summary:
This work reports on an observational study of a newly detected Galactic supernova remnant (SNR) in gamma-rays using Fermi-LAT, which claims new discoveries as follows: (1) the SNR shows a north-south segregation in the character of its gamma-ray spectra, for which the authors claim to be the first ever case found to display a hadronic vs leptonic nature of the emission inside a single SNR; (2) the authors claim that this SNR is originally a binary companion to the progenitor of the famous IC 443 in the same region based on sky proximity, distance estimates and binary population synthesis result; (3) they also use a spatial correlation between H-alpha emission with the newly found GeV gamma-rays in the northern part to support its hadronic origin, in particular through the "crush-cloud" mechanism where re-accelerated Galactic cosmic rays compressed inside a radiatively cooled region behind the SNR shock is responsible for the high-energy radiation.

Main comments:
1) On discovery #1 listed above - I am sure the authors are aware of other systems which are known to show (or at least have shown strong hints on) such a mixed contribution from both hadronic and leptonic gamma-rays. One might list Vela Junior, SN1006, RX J1713, CTB 109, etc. It would be an over-statement to say this work encompasses the first discovery of such a mixed emission mechanism. Along the same line, the approach of using spatial correlation of gas distributions with gamma-rays for the identification of gamma-ray emission mechanism is not new and has been used in other works by people like H. Sano, Y. Fukui and co-workers for years, arguably in a much more sophisticated and quantitative way than what the authors have done here. Further support for the novelty of this work to claim such a first discovery is in place, or else I would suggest the authors to tone down their claim and acknowledge similar previously found results.

2) On discovery #2 - while providing circumstantial supports such as distance estimates and a viability test through population synthesis is highly appreciated, but as indicated in the paper title which puts it as "the first observed binary system's aftermath" so that it is actually pointing toward a solid confirmation, it is again risking the danger of an over-statement. Claiming a companion origin of this SNR with IC 443 in particular needs a smoking gun evidence. Is there any observational characteristics of this SNR discovered which uniquely proves that it originated from a binary progenitor system? Any evidence of envelope stripping from the SNR dynamics and emission? etc. It is intriguing indeed that this SNR does look to be related to IC 443 in some way, but within the context of this work in its current state, I worry that it would be

premature to over-interpret the findings as a discovery.

3) On discovery #3 - the usual explanation of H-alpha filaments is from a fast SNR shock running into a partially neutral medium which triggers charge exchange interactions, so that the filamentary look in H-alpha is coming from the post-shock ionization length scale. This work claims otherwise that the shock has slowed down to enter the radiative regime so that post-shock compression inside the cooling gas (the filament) is responsible for the enhanced GeV emission in the context of the so-called "crushed cloud scenario". Especially in a situation without any radio spectrum or other signatures of radiative shocks such as optical forbidden lines or OH masers to back up, it is unclear to me how the authors can pin down the emission mechanism using one-zone models for gamma-ray-only spectra with a high confidence.

4) Age estimates - how can one estimates the age of the SNR using an assumption of Sedov-phase evolution while simultaneously employing the "crushed cloud" scenario for the gamma-ray emission which requires the SNR shock to have evolved into the radiative phase for a substantial period? Is the use of a Sedov-like discussion applied in an averaged sense for the SNR as a whole? If so, how can one justify this without a separated estimation of dynamical ages between N and S which are apparently very different e.g. from the spectral model findings? Please clarify.

Minor comments:

1) On the crushed cloud model - some major references are missing, especially the more dedicated numerical works following up Uchiyama et al. (2010).

2) On the energetics estimation for gamma-ray emitting CRs - since there are large uncertainties involved in the ambient environments in the spectral models, it is more informative to provide numbers for the energetics in a way scalable with target gas density etc.

3) There is a mentioning of the "cooling break" for the electrons, but it is not clear to me how its estimation influences the spectral modeling. The necessity of introducing such a break is also unclear. The notion of "cooling break" arises from the argument of a balance between cooling and dynamical (or particle injection) timescales. It's actual appearance in the spectra is usually exaggerated by one-zone models with a single particle population and a constant B-field ignoring the spatial variation of both and time evolution of the shock and post-shock gas. In other words, the cooling time scales and so on are usually both time and spatial dependent. The "break" in reality is hence much more smeared out when one volume-integrates the electron spectra over the whole remnant at any given age, such as in a hydrodynamic simulation.

4) Table 3 - given the very high uncertainties, it would be more helpful to also provide the "allowable" ranges of the key parameters, rather than only printing the fitting values from the few models being tried out.

Reviewer #4

(Remarks to the Author)
This paper details the observation and spectral analysis of the supernova remnant G189.6+3.3 in the region of the well known SNR IC 443 and the HII region S249. The authors analyse data from the Fermi-LAT telescope, combining this with multi-wavelength data to model particle acceleration in this SNR. From this data the authors come to two important conclusions, that the northern region of the SNR is dominated by hadronic emission of gamma-rays and that combined with the spatial correlation with the S249 this indicative of an interaction with the HII region. The authors then propose the the two SNRs in this region originate from high mass star binary system and derive a range of explosion times from these two progenitor objects.

Generally speaking this is a well written paper, with the data analysis and interpretation being of a high standard with the authors going to significant efforts to back up their suggestion that G189.6+3.3 and S249 lie at the same distance. There are a few areas where some more explanation and justification could be provide to better demonstrate that both IC 433 and G189.6+3.3 are located at the same distance and hence originate from binary progenitors.

I will split my comments into more major or general comments, followed by minor comments, typos etc.

General comments

When reading some areas of this paper some of the flow of the paper can be lost by the sentence structures used. For example:
Ln 75 “Follow up studies at higher energies, that is X-rays, have resulted…” or
Ln 86 “gamma-ray emission, given that relativistic hadrons only radiate…, above the energy threshold…”
I would ask the authors to take a read through the introduction and check to the flow of the text and perhaps use a more simple sentence structure e.g. “Follow up studies at X-ray energies have resulted ”.

The authors invest significant effort in showing that the the distances of the SNR G189.6+3.3 and the HII region s249 are at a compatible difference with one another. These arguments combined with the interaction increased brightness in the s249 region and hadronic emission is generally quite convincing. However, it is not clear from either the introduction or the work presented in the papers is how strong the correlation is between IC443 and the HII region. I think it is important to discuss the strength of this evidence as it is equally at important to support the conclusion of a binary system.

For example reference 58 in the paper suggests that s249 is located 600 pc in front of IC 443, this should be discussed.

I think it would be much clearer to understand the analysis if there was a plot included showing all the best fit spatial models on a TS map (or add this to an already existing map), as at the moment it is not clear exactly which region is where. This is particularly important as many different models are used. It is, for example, not clear to me exactly what regions are used for the extraction of the northern and southern regions, this needs to be made clearer. Also calling these north and south may be a little confusing, because if I understand correctly they are overlapping.

Minor Comments

Ln 82: And -> and
Ln 100: is the G189… -> is G189…
Ln 122: it may be worth mentioning why this source is no longer considered linked
Ln 167: is it possible to shorten gigaelectronvolt to GeV after the first usage? Or is this a journal requirement?
Ln 174: I think the eROSITA paper should be referenced here
Ln 202: Is it really an excellent candidate to be a significant neutrino emitter, although seemingly hadronic dominated it is also distant and not so bright. Was an expecting neutrino flux calculated?
Ln 256: “giving birth” -> producing
Ln 274: I’m not sure how this is mimicking an IC peak, is it not the interpretation that this is actually an IC peak?
Ln 294: I’m not sure triggers is the right word here, it acts a a target for hadrons
Ln 327: extremely well localised is a bit difficult to interpret without any numbers, how good is the positional localisation?
Ln 370: It is not very well explained what the COMPAS code actually does and what is simulated here, it is simply stated that binary systems were simulated, more explanation would be needed
Ln 460: remove “sophisticated”, it is just a python interface

Section 4.2.1.: I’m not sure why there is a split here to talk about eROSITA between LAT analysis sections, also this section is more a description of the data and not morphological analysis

Ln 514: employed -> included?
Ln 515: this sentence is a bit confusing please consider restructuring
Ln 526: PS map needs explanation here at first usage, it is not a map that many Fermi users are familiar with
Ln 667: Was the morphology of the fitted 2d gaussian compared to the Halpha filament?
Ln 684: Is there a statistical test of the preference for the LogParabola model over PL?
Ln 703: Once the spectrum is extracted with the second model, how is this used in calculating systematics? And what ware the values?
Ln 750: The Alpha measurement is mentioned here, but again this data suggests s249 is much closer
Ln 764: The value os 3x10^4 yrs is repeated several times, but is only derived in one location
Ln 779: “one derives a local” this is confusing, please consider restructuring the sentence
Ln 801: Why was one set of values chosen to use in the modelling over the other?

Ln 847: “”Nevertheless…” I’m not sure what is sentence is adding other than SED modelling was performed, condor simplifying
Ln 867: Naima should be \em{naima} I think
Ln 888: Were these values fit to the data? Or are hey just a representative set of parameters? Is it possible to provide error on these parameters? Or do the others think with the other assumptions being made this
Ln 892: Again is there a statistical preference of the hadronic vs leptonic model?
Ln 920: I guess this does not yet indicate this is viable, rather it is intended to show this
Ln 985: I got a bit lost in the text where the 2 Jy value comes from, is this quoted earlier?
Ln 987: It’s not clear to me why future radio observatories will do better here

Table 3: I think this is a bit confusing, as far as I can tel some of these values have come from the SED, whereas some (like the B field for the norther component) come from elsewhere.

Fig 2: I generally think that adding a flat cut-off to the map below a fixed TS value is quite misleading as it can lead to the interpretation of structures in the map as seeming clearer than they really are. This is especially misleading as the TS threshold is changing between the three energy bands

Fig 3: It seems a bit concerning that the PL fit is violating one of the upper limits, this should maybe be commented on. I would imagine this is not statistically significant, but it is not possible to say as no T/S preference is given for log parabola vs PL

Version 1:

Reviewer comments:

Reviewer #1

(Remarks to the Author)
I thank the authors for their detailed responses to my original report and for the revisions made to the manuscript. Several of

my original concerns have been addressed. I am also satisfied that the most egregious overstatements in the Discussion have been toned down. However, I have significant remaining concerns, in particular about the overall readability of the manuscript.

Spectral index of the Hα filament: The authors' response to my question about the radio spectral index of the filament conflates two distinct issues. My original point was that the challenges described in Section 4.6 regarding flux density measurements of extended sources do not straightforwardly apply to the measurement of a spectral index for a compact, narrow filamentary structure that might be well sampled. The authors argue instead that mismatched angular resolutions and diffuse background contamination make a spectral index measurement unreliable. This is a legitimate practical concern, and one that can be contained within an uncertainty measurement (that’s what error bars are for).

I also note that what the authors present as a radio flux density "estimate" for the filament region is in fact a theoretical prediction scaled from the Cygnus Loop using ratios of compressed density and magnetic field strength. The simplest and most convincing approach would be to measure the flux density within a defined aperture in the 1.4 GHz radio map, subtract the background using appropriate control regions, and quote the result with an uncertainty. As far as I remember, the CGPS map (which I think is what Fig. 1, top left is), has zero spacings.

The binary supernova claim. My more serious concern relates to the central claim of the paper, namely that G189.6+3.3 and IC 443 constitute the first observed binary supernova system. While I acknowledge that the authors have made genuine efforts to strengthen this argument I still do not find the overall case convincing. For the probability calculation, both estimates rest on a surface density of Galactic SNRs derived from the Green catalogue, which contains 310 entries, which we all understand to be a severe underestimate of the true SNR population. More importantly, SNRs are not uniformly distributed across the Galactic plane: they cluster in star-forming regions and along spiral arms, and the IC 443 complex lies within the Gem OB1 association, a region of recent massive star formation where the local surface density of SNRs is substantially higher than the all-sky average.

Beyond the probability calculation, the broader binary argument rests on a chain of evidence in which each link carries substantial uncertainty. The distance estimates for the two remnants are compatible within their error bars, but those error bars are large. The binary population synthesis demonstrates that the proposed scenario is physically possible, but given the breadth of the allowed parameter space, this is not a strong discriminating result. The jet-like features and ejecta abundances are suggestive but explicitly acknowledged by the authors themselves to be non-unique signatures of a binary origin. The most compelling evidence for a physical association between the two remnants remains, as I noted in my original report, the fact that both appear to interact with the same HII region, S249. However, the gap between "both remnants interact with the same dense structure" and "these remnants originate from a binary progenitor system" is a large one, and the paper does not bridge it convincingly.

However, that is my own interpretation of the evidence that the authors have provided (although I do note that the probability of chance alignment calculated is an underestimate, as it does not accurately take into account the specific Galactic location of the sources), and I understand that part of the point of a paper is that the authors have a point that they want to make and some evidence that supports it. Which brings me to my next comment.

General remarks on the manuscript: I am concerned about the overall readability and coherence of the revised manuscript. The authors have responded to each referee comment individually, but the paper does not read as a coherent whole. Some basic examples: there are frequent forward references that are not always necessitated by the structure of the paper (e.g., Section 4.5 references Section 4.6) and the opening of Section 4.5 does not even explicitly state which of the two SNRs that the paper discusses is being modeled. Also, although I appreciate that the authors have stepped up their figures game, for a paper whose central claims rest so heavily on morphological and spatial coincidences, it is surprising how little use is made of the figures to guide the reader through the argument.

I would also recommend some serious editing. If I had not refereed this paper, I would not have made it past the introduction simply because of the quality of the writing. The title itself is a harbinger of what’s to come. Between lines 1153 and 1160, four consecutive sentences start with: “Consequently, …,” “Importantly, …,” “Consequently, …,” “Subsequently, …” I would urge the authors to try to read the text as a new reader would, and ask themselves whether they can follow the argument.

The paper attempts to establish many results simultaneously, and the through-line of the argument is frequently lost. I would strongly encourage the authors to undertake a thorough structural revision of the manuscript, with particular attention to ensuring that each claim is clearly stated, the supporting evidence is presented in a logical sequence, and the distinction between what is established and what is speculative is made explicit throughout.

Reviewer #2

(Remarks to the Author)
I thank the authors for their substantial revision. The manuscript addresses several of the concerns raised in the previous version. These additions have improved the manuscript and made the interpretation more complete. In its revised form, the paper presents noteworthy results that are clearly relevant to the field, and the analysis is now more convincing and better contextualized within the broader literature. The study compares well with the established literature and, in my view, contains sufficient scientific value to make it a potentially publishable contribution.

That said, I remain unconvinced that the most far-reaching claims are demonstrated at the level currently implied by the title, abstract, and several statements in the main text. In particular, the binary-supernova interpretation is now better motivated, and the newly added chance-alignment estimates are useful additions that directly address an important concern from the previous report. However, I still find that this interpretation is presented somewhat too strongly relative to the available evidence. The probability estimates are informative, but they are approximate and do not constitute formal model comparisons. As such, the current results support plausibility, but not yet a unique inference.

I therefore recommend that the authors moderate the wording throughout, especially when expressions such as “first known example” or “strong evidence” are used. At present, the manuscript makes a compelling case for a very interesting candidate system, but not yet, in my view, for a secure first detection.

Reviewer #3

(Remarks to the Author)
This version of the manuscript has received major improvements and extensive addition of text for clarification. I can see most of the raised concerns have been addressed and I can now recommend it for publication without problems. The title is still sounding a little awkward. I would suggest dropping the parentheses and even better the colon as well. I also cannot fully understand the meaning of "duel" here as there is no competition involved as far as I understand. I guess the authors are intending to mean "co-existence" instead? Other than that I do not have any further comment.

Reviewer #4

(Remarks to the Author)
Report - 2nd round

The authors have made significant changes to the manuscript to address the comments from all referees and I would like to thank them for their very comprehensive response to referee comments. Generally speaking I think the authors have addressed well most of the comments I made in my report.

The most significant question I had was the relative distance of s249 and IC 443 and some measurements which suggested the two were not located at the same distance. The authors addressed these concerns in both their responses to comments and within the paper itself, describing the difficulties in interpreting velocity measurements as a distance in this region. They also provide explain the evidence for the co-location of these objects through signs of interaction between the two.

Most suggestions regarding wording were adopted or addressed in some way.

I still think the issue of having a hard cut in the TS maps could introduce some apparent structures in the maps which are not truly present, the choice of this cut-off value could change the apparent size of the sources to the reader. Especially seen as the source significance is quite high I don’t think the maps would look that different without it. However, as the authors explain this thresholding an it is not important for the results of this paper and simply to make the plot easier to read, I would simply ask that the authors consider changing this rather than insisting.

I accept the authors statement on the IC vs hadronic model comparison, as for example it may be possible to fit the northern spectrum with an IC model with totally implausible parameters which might also give a reasonable fit statistic, even though being completely unphysical. However, this is important to make note of. Currently (as far as I can see) the only mention of preference is that is does not show an IC peak, this perhaps would be worth a little more explanation as to why there is no plausible IC model that can fit this region.

Version 2:

Reviewer comments:

Reviewer #1

(Remarks to the Author)
I thank the authors for addressing my comments and re-structuring the paper to make for a more coherent read. I do not need to see this paper again

Reviewer #4

(Remarks to the Author)
Thanks again to the authors for addressing the reviewer comments in so much detail. I think my remaining comments were well addressed and I have no further comments.

Open Access This Peer Review File is licensed under a Creative Commons Attribution 4.0 International License, which permits use, sharing, adaptation, distribution and reproduction in any medium or format, as long as you give appropriate credit to the original author(s) and the source, provide a link to the Creative Commons license, and indicate if changes were made.
In cases where reviewers are anonymous, credit should be given to 'Anonymous Referee' and the source.
The images or other third party material in this Peer Review File are included in the article's Creative Commons license, unless indicated otherwise in a credit line to the material. If material is not included in the article's Creative Commons license and your intended use is not permitted by statutory regulation or exceeds the permitted use, you will need to obtain permission directly from the copyright holder.
To view a copy of this license, visit https://creativecommons.org/licenses/by/4.0/

We thank the reviewers for their careful assessment of our manuscript and for providing constructive suggestions for improvement. Below, we respond to the specific comments. Changes to the manuscript are indicated in bold.

Reviewer 1#:

I followed the prompts listed in the instructions to reviewers page.

===
Key results

This work presents three key claims:

SNR G189.6+3.3 shows gamma-ray emission of leptonic and hadronic origin in two distinct regions.
The progenitors of SNR G189.6+3.3 and IC443 formed a binary system (most likely of massive stars, possibly including an LBV) with explosions happening "shortly" (<100 kyr) after each other.
The emission from a particular region of the source (referred to as “the H\alpha filament”) is compatible with a proton re-acceleration scenario.

The evidence presented is (a) an original analysis of 16 years of Fermi-LAT data (b) a compilation of archival values and a MW SED modeling and (c) some crushed cloud modeling of the hadronic component. Claim 2 is speculative and only supported circumstantially by the data.

===
Validity:

I did not find any flaws that should prohibit the manuscript’s publication.

===
Originality and significance:

The conclusions are original, and I think would be interesting to people in the fields of CR physics, Galactic astronomy, SNR evolution and feedback, and stellar populations.

==
Data & methodology:

The presentation of the data in the text and images is sometimes confusing. I would have appreciated a better display and labeling of the features discussed in the text in a large three-color image (e.g. DRAO, WISE, eROSITTA, or radio, mm, IR) to have a better sense, by eye, of the thermal/non-thermal components, the areas of overlap, and the general structure of the regions and how they interact with each other.

Response:

We present a three-colour WISE–eROSITA–Fermi-LAT composite image (Fig. 1), together with a separate radio-continuum figure showing data from three distinct radio surveys (Fig. 12). The three-colour image in Fig. 1 is intended as a synthesis figure and is designed precisely to illustrate the location, spatial extent, and overlap (i.e. potential interaction regions) of the main components discussed in this work: the G189.6+3.3 SNR, the IC 443 SNR, and the S249 HII region. All of these structures are explicitly identified/labeled and colour-coded in the figure. As such, Fig. 1 directly addresses the referee's request for a labelled overview of the key regions and their relative geometry. For this reason, we placed this figure at the beginning of the manuscript and referenced it already in the Introduction, so that readers can immediately gain a clear, global view of the system, the positions of the sources relative to each other, and possible interaction zones. Following this request, we have updated Fig. 1 to include the spatial extent of G189.6+3.3 and the Hα filament, making these features clearer (see also our response to Suggested Improvements, point 2).
Regarding the suggestion to include an additional three-colour image incorporating radio data (e.g. radio–WISE–eROSITA), we considered this option carefully. However, the radio emission associated with G189.6+3.3 is incomplete and faint, consisting mainly of a weak arc along its northern boundary, and is much weaker than the radio emission from IC 443. A three-colour composite including radio emission from both remnants would therefore be visually dominated by IC 443 and would obscure the structures most relevant to G189.6+3.3. Now that the extent of G189.6+3.3 and the Hα filament have been added to Fig. 1, we believe that introducing an additional three-colour composite differing only by one wavelength band would be redundant.

By "confusing" I mean things like: in the caption to Fig. 1 it says: "spatial comparison of X-ray, gamma-ray, and CO data," but then there is no display of the CO data, just a mention that the authors have looked at it. Some of the datasets referenced are outdated

(e.g. for CO they reference the Dame survey, whereas the higher resolution MWISP is available for this area). Fig. 4 has no coordinates. In this sense, the presentation is sloppy; these are some examples.

Response:

First, the referee is correct that Fig. 1 presents a spatial comparison of X-ray, gamma-ray, and WISE data, rather than CO data. The reference to CO in the original caption was therefore misleading, and we have corrected the caption accordingly. At the time the original analysis was carried out, the MWISP data were not yet publicly available, and only lower-resolution CO surveys (e.g. Dame et al. 2001) could be consulted. We examined those data and found them to be in very good agreement with the morphology traced by the WISE mid-infrared emission. This is expected, as both tracers indirectly probe the same underlying dense material (gas+dust): CO traces cold molecular gas, while WISE mid-IR emission traces warm dust mixed with that gas. Given the significantly higher angular resolution of the WISE data, we therefore chose WISE as the primary tracer in Fig. 1. We have also examined the MWISP data; for additional details see our response to Suggested Improvements, point 1. Fig. 4 has been revised and is now included as an inset in Fig. 1 to provide its spatial context, while preserving the spatial scale through the arcmin scale bar (see also our response to Suggested Improvements, point 2).

A lot of attention is paid to "the H\alpha filament." In line 989 the authors state that the radio emission in the H\alpha filament is enhanced. I downloaded the GLEAM and CGPS 1.4 GHz maps and I can't see any enhancement (although this is tricky since Fig. 4 does not have axis labels). If I am wrong about the location of the filament and the radio emission is indeed enhanced, and visible in the different radio maps, it would be nice to comment on its spectral index (estimating this for a thin filamentary structure does not present the challenges outlined in p. 22, which are more to do with measuring the flux **density** (anything measured in Jy is a flux density —this is another recurrent mistake in the manuscript) of an extended source. Is the radio spectral index consistent with a reacceleration scenario?

Response:

Regarding the first point, Fig. 12 (upper panels) was specifically included to assess whether there is a radio enhancement at the location of the Hα filament. We present radio continuum data from three independent surveys (DRAO, GB6, and GLEAM). We agree that the significance of the radio enhancement is survey dependent. In

particular, it is not readily apparent in the GLEAM data, where the combination of limited angular resolution and sensitivity to large-scale emission naturally dilutes narrow filamentary features. In contrast, the enhancement is more clearly discernible in the higher-frequency DRAO and GB6 data, where it appears over a relatively small angular extent.

To clarify the spatial correspondence, the upper panels of Fig. 12 show the DRAO 1.4 GHz map both without overlays and with the position of the optical Hα filament superimposed (upper right panel of Fig. 12). In this representation, the filament position is spatially coincident with a localized increase in radio brightness.

Concerning the second point, namely whether a radio spectral index can be reliably measured for this filamentary structure, we discuss the issues in the context of the flux density estimate in the second half of section 4.6 (starting at line 954 - original manuscript or lines 1407-1419 in the revised version).

We understand that if the case was a bright isolated filament background issues would not be dominant. But for a faint filamentary emission embedded in structured backgrounds, uncertainties in background subtraction do not cancel in spectral-index measurements. In fact, such a measurement becomes even more difficult given that each instrument has its own background.

We have now included some discussion relevant to the spectral index estimate as well. While a spectral index measurement could, in principle, provide additional diagnostic power, in practice it is not robust with the currently available data. The filament is faint and narrow, and the radio surveys used here differ substantially in angular resolution, spatial filtering, and sensitivity to diffuse emission. As a result, any spectral index estimate would be dominated by systematic uncertainties associated with beam dilution, background subtraction, and mismatched spatial scales, rather than reflecting the intrinsic emission properties of the filament.

Although a spectral index can formally be derived from two frequencies, a meaningful measurement would require at least three frequencies with matched angular resolution, comparable sensitivity to the relevant spatial scales, and sufficiently high signal-to-noise, which are not currently available for this structure. For example, the filament is clearly detected only in two of the available radio surveys (DRAO and GB6), while it is not clearly visible at all frequencies. Any attempt to derive a spectral index would therefore require convolution to a common angular resolution between datasets. Even after such processing, the measurement would still rely on a very faint structure embedded in structured background emission, such that the resulting spectral index would be dominated by systematic rather than statistical uncertainties. For this reason, we refrain from quoting a spectral index. Instead, we compare the observed radio flux density at the filament location with the flux density predicted by the re-acceleration model. We find that the observed values are consistent with the model expectations, providing a more

robust and physically meaningful test of the re-acceleration scenario than a poorly constrained spectral index.
Finally, we acknowledge the referee's comment regarding terminology and have corrected "flux" where "flux density" is intended.
We hope that these clarifications, together with Fig. 12 and the expanded discussion in section 4.6, address the referee's concerns.

===
Appropriate use of statistics and treatment of uncertainties:
I think the statistics are handled correctly.

===
Conclusions:
The results are interesting; finding a source that shows both leptonic and hadronic emission will certainly provoke a lot of analysis in the field, and as the authors note will be a prime target for neutrino observations. It is a bit difficult for me to comment on the soundness of claim #1, since I am not an expert on Fermi data analysis, which underpins the result. As per claims #2 and #3, I think that they are both plausible. I find that the most solid piece of evidence that IC443 and SNR G198.6+3.3 are related (much more so than the distance/age estimates, which have large uncertainties) comes from the fact that they are both interacting with the same HII region, as the authors show here. Going from there to a claim that they used to be part of a binary system seems a bit of a stretch, and some of the claims that the authors make in the discussion (e.g. "Such results can, for the first time, revolutionize our understanding of stellar life cycles. They are also crucial for constraining models of stellar evolution, star formation, and even testing gravitational theories") see overblown, reading more like a grant application than a paper.

Response:

We have carefully reviewed the manuscript to identify and address any potential overstatements. We believe that the claims presented in the Results section are appropriately presented (re-structured in the revised version) and supported by the data, without over-interpretation. In response to the referee's comments, we have further refined a few arguments in the Discussion to ensure a more cautious and balanced presentation.

===
Suggested improvements:

Perhaps the authors have already done this and decided against showing the image because it might not be very informative, so I am happy to defer to their judgement, but I would have liked to see that indeed the CO emission is spatially coincident with the hadronic gamma-ray emission. Could the authors comment on what kind of molecular observations could unambiguously confirm the association between the CO and the gamma-rays? Although I agree with the authors that the spatial coincidence is very suggestive, a chance alignment cannot be ruled out —spectral evidence (e.g. from the line ratios of certain components, or the emission of certain tracers) is necessary, in my opinion, to confirm this proposed scenario. I would urge the authors to use MWISP, but even if they only use the Dame data —what is the velocity of the associated cloud components? Does it match (using a rotation curve) the distance of the SNRs?

Response:

In response to the referee's suggestion, and following the recent public release of MWISP DR1, we have downloaded and inspected the MWISP 12CO (and 13CO) data for this region. We produced velocity-integrated CO maps over the range −100 to +100 km/s. We find that the S249 H II region is primarily associated with CO emission in the −10 to +10 km/s interval, while the structures spatially coincident with the two SNRs can be further confined to −5 to +5 km/s. Given that IC 443 and G189.6+3.3 lie at distances of ~1.5–2.0 kpc, the expected kinematic velocity from smooth Galactic rotation along this line of sight is only a few km/s in magnitude; therefore, CO emission at a few km/s negative to a few km/s positive is compatible with material at the distance of the SNRs once local (non-circular) motions are taken into account, as expected toward the Galactic anticenter.
The MWISP data reveal the same large-scale structure seen in the WISE emission (white contours in the images attached below) and, importantly, show a clear spatial correspondence with the hadronic component of the G189.6+3.3 gamma-ray emission (green box in the image attached). Because the MWISP CO and WISE data trace essentially identical structures in this region, including both datasets in Fig. 1 would be largely redundant. Instead, we now explicitly describe in the text (lines 471-475 & 1964-1968 in the revised version) the results of our inspection of the MWISP data and the observed spatial correlation with both the WISE morphology and the gamma-ray emission. We provide here the MWISP 12CO image per the referee request at the end of this reply.
Regarding the referee's question about what molecular observations could unambiguously confirm an association between the dense gas and the gamma-ray emission, we have added relevant discussion in the paper (mainly lines 452-478 in the revised version). The evidence currently supporting an interaction scenario already includes: (i) the spatial overlap between the hadronic gamma-ray

component associated with G189.6+3.3 and the S249 HII region, (ii) the ultraviolet signature of the northern shock front, which provides direct evidence for shock deceleration due to interaction with dense material, and (iii) optical forbidden lines (now discussed in more detail - mainly in lines 410-418 and 1395-1399, with additional brief mentions elsewhere in the text, in the revised version).

As the referee is likely aware, this region lies close to the Galactic anti-center, where kinematic distances inferred from CO radial velocities are intrinsically poorly constrained. Galactic rotation produces velocities close to 0 km/s over a wide range of distances, and modest non-circular motions can easily shift velocities by several km/s. In practice, CO emission plausibly associated with material at the distance of IC 443, G189.6+3.3, and S249 is typically bracketed within -10 to +10 km/s, which is also the velocity range over which the S249 HII region becomes apparent in the MWISP data.

We hope that these clarifications, together with the corrected figure caption, the inclusion of the MWISP CO data in the text, and the expanded discussion of molecular diagnostics, address the referee's concerns.

In the figures provided below, the background shows the MWISP 12CO emission, the white contours correspond to the WISE data, the green square indicates approximately where G189.6+3.3 overlaps with S249 and the green circle the approximate position of IC 443, for context.

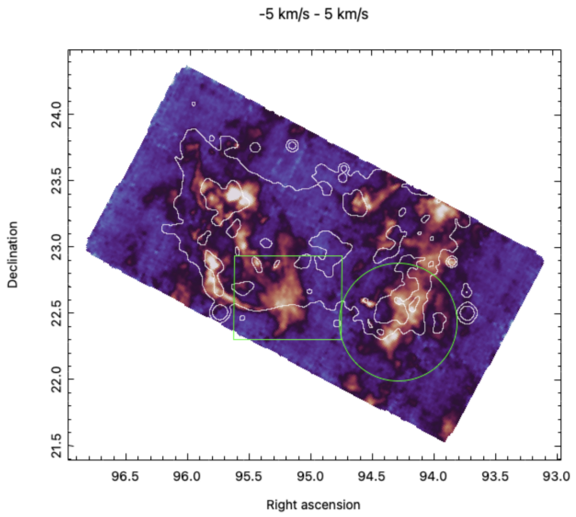

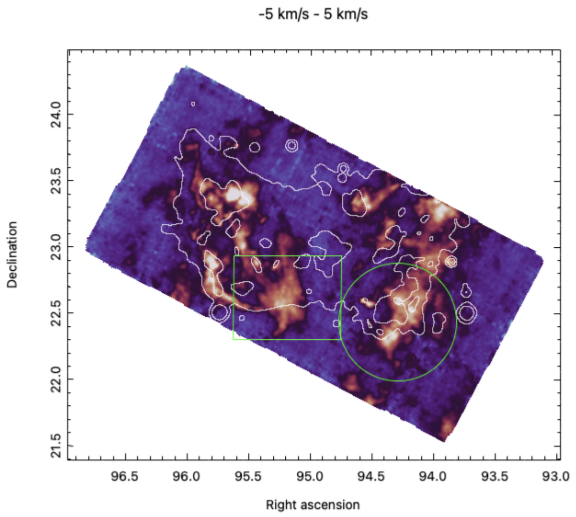

Please, have a figure that labels the characters in this paper, and particularly their extent (IC443, G189, HII reg S249, the H\alpha filament). In the case of zooms in of particular areas (e.g. Fig 6) it would help to show the inlet in a larger figure (although since Fig. 6 does actually have coordinates this is not as crucial).

Response:

As mentioned in the Data & Methodology comments (point 1), we present a three-colour WISE–eROSITA–Fermi-LAT composite image (Fig. 1), together with a separate radio-continuum figure showing data from three distinct radio surveys (Fig. 12). The three-colour image in Fig. 1 is intended as a synthesis figure and is designed precisely to illustrate the location, spatial extent, and overlap (i.e. potential interaction regions) of the main components discussed in this work: the G189.6+3.3 SNR, the IC 443 SNR, and the S249 HII region. All of these structures are explicitly identified/labeled and colour-coded in the figure. As such, Fig. 1 directly addresses the referee's request for a labelled overview of the key regions and their relative geometry. For this reason, we placed this figure at the beginning of the manuscript and referenced it already in the Introduction, so that readers can immediately gain a clear, global view of the system, the positions of the sources relative to each other, and possible interaction zones. Following this request, we have updated Fig. 1 to include the spatial extent of G189.6+3.3 and the Hα filament, making these features clearer (see next paragraph).
We acknowledge that, in its original form, Fig. 1 did not explicitly show the full spatial extent of G189.6+3.3 or the position of the Hα filament. We initially chose not to include these elements because doing so risked overcrowding the figure and obscuring the emission already displayed. However, in response to the referee's comment, we have now incorporated both the extent of G189.6+3.3 and the Hα filament in Fig. 1 by overlaying them with significantly reduced opacity. This addition provides useful spatial context for the extension of G189.6+3.3 and the interaction region of the latter with the S249 HII region while preserving the readability of the underlying multi-wavelength emission.
For these reasons, we opted to retain Fig. 1 as a clean, global synthesis image and to present the radio data separately, where their morphology can be discussed in greater detail without compromising clarity. Additional explanatory information has also been added to the figure caption to further guide the reader.

The manuscript contains only two zoomed-in images, shown in Figs. 4 and 6. Figure 6 includes explicit coordinate axes, and therefore we chose not to include an inset for this figure, as the location of the zoomed region can be unambiguously identified directly from the axes (as also acknowledged by the referee).

Figure 4, on the other hand, does not display coordinate axes. This was a deliberate choice, as the figure is intended to highlight only the angular extent of the structure (indicated by the 3″ scale bar), and its location was conveyed by using the same blue Hα filament morphology shown in Fig. 2. However, we recognize that this approach may not be sufficiently clear to all readers.
In response to the referee's suggestion, we have revised the presentation by showing Fig. 4 as a large inset on Fig. 1, which provides the larger-scale context and clearly indicates the location of the zoomed-in region. This modification improves the clarity of the presentation while preserving the original intent of Fig. 4.

We hope that these revisions and clarifications address the referee's concerns and improve the overall clarity of the presentation of the distinct components.

Please give a thorough read to all captions and make sure that what is discussed in the caption is actually (a) relevant to the figure and (b) not first explained in a caption (e.g. the caption of Fig. 6 is the first time that the EXPLORE G-Tomo tool is mentioned —I would have appreciated a discussion in the text of how this tool works, and whether it is indeed suitable for an extended source, since my eyebrows were raised by the tiny uncertainty reported in the distance estimate from the optical extinction).

Response:

We have now thoroughly re-read all figure captions and revised them where necessary to ensure that (a) the caption content is directly relevant to the figure and (b) important concepts are not introduced for the first time in a caption.
In particular, the EXPLORE G-Tomo tool is now introduced and described in the main text (lines 1048-1068 in the revised version). G-Tomo does not introduce any new data or methodology beyond what is already described in the text. It is simply a visualization and query interface that combines the latest three-dimensional extinction datasets to produce extinction–distance relations along a given line of sight. In brief, G-Tomo provides an estimate of the line-of-sight extinction as a function of distance for a given sky direction, i.e. for a single set of coordinates (effectively a "pencil-beam" sightline). For this reason, its direct application to an extended source requires some care. In our case, we therefore queried the tool at multiple positions across the angular extent of the SNR to assess how strongly the inferred extinction–distance relation varies over the remnant area, and to quantify the resulting systematic uncertainty when applying the method to an extended object. We note that the results of these tests, as well as a discussion of the broader distance uncertainties associated with this method, are presented toward the end of

Section 4.4.1, in the paragraph dedicated to G189.6+3.3. We have now made this connection clearer in the manuscript.

==

References:

In light of all the attention that is paid to VHE instruments in recent months, I am missing a comment on the LHAASO upper limits for this source. The E_max values mentioned in line 889 are within the WDCA. Fig. 11 suggests already that perhaps the LHAASO UL are not super useful, but not mentioning other gamma-ray observations of the area seems like a missed opportunity.

Response:

We agree with the referee that acknowledging other very-high-energy gamma-ray observations of the region improves the completeness of the manuscript. We have therefore incorporated a discussion of existing TeV results and commented on their currently limited constraining power for this source (lines 693-721 in the revised version). We now include a qualitative comparison with available HAWC upper limits and the LHAASO detection of component C1 (northeastern part of IC 443), which is at least partially spatially coincident with G189.6+3.3 (see updated Fig. 10). These TeV measurements provide potentially interesting constraints; however, they should be interpreted with caution, as the HAWC and LHAASO results are in tension, likely due to the use of different spatial models. Both of these spatial models also differ from the morphology inferred from eROSITA, making a formal fit to the TeV data premature.
For this reason, we do not use the TeV data to constrain our model, but we include them on the SED for completeness and to inform future observations, particularly with CTA. Importantly, the detection of gamma-ray emission from G189.6+3.3 reported here for the first time establishes this remnant as a potential contributor to the very-high-energy emission of the IC 443 complex. Disentangling such a contribution will require future TeV observations with improved angular resolution and sufficient sensitivity to faint, extended emission.

Finally, given the strong spectral curvature of the northern component, we do not expect a significant TeV contribution from that region and therefore did not modify the corresponding SED.

==
Clarity and context:

The abstract and introduction are clear, there is no conclusions section. As I mentioned above, some of the claims in the discussion are far-fetched.

Response:

As mentioned above, we have carefully reviewed the manuscript to identify and address any potential overstatements. We believe that the claims presented in the Results section are appropriately presented (re-structured in the revised version) and supported by the data, without over-interpretation. In response to the referee's comments, we have further refined a few arguments in the Discussion to ensure a more cautious and balanced presentation.

Reviewer 2#:

The manuscript presents the detection of extended GeV emission from a region spatially coincident with SNR G189.6+3.3 (hereafter referred to as G189), utilizing 16 years of Fermi-LAT data. The manuscript claims that the morphological and positional analyses of this GeV emission are consistent with the X-ray shell of the SNR G189.6 as observed by eROSITA. The manuscript further uses eROSITA, Swift UVOT data, and multi-wavelength SED modeling to suggest that in the northern part, gamma rays are produced from the hadronic interaction between protons reaccelerated within the H-alpha filament via adiabatic contraction, with the cold protons in the nearby S249 HII cloud, whereas in the southern region, gamma rays are produced via the leptonic inverse-Compton channel. The manuscript further proposes that IC 443 and G189 together are the first observationally supported binary supernova systems, as determined through various analyses for age and distance estimations, including binary population synthesis using the COMPAS code.

The topic is highly interesting, the analysis is ambitious, and the potential implications, binary supernova remnants and clear hadronic/leptonic spatial separation, would be significant in the field of high-energy astrophysical systems if robustly demonstrated. The manuscript contains extensive observational and theoretical analysis. However, several key claims lack sufficient quantitative demonstration, and methodological clarity needs improvement before the results can be considered for publication. Therefore, I recommend the manuscript for publication in Nature Communications only after the suggested revisions have been made. I outline my major concerns below.

General comments/suggestions:

1. The most significant claim that IC 443 and G189 form the first observed binary supernova system needs to be sufficiently supported.

The distance estimates for both remnants rely on extinction-based arguments with non-negligible uncertainties (worst case scenario being around 500 pc, which seems very large if it is really a binary system). Furthermore, the overlap of gamma ray data with the S249 HII region does not by itself guarantee identical distances between G189 and IC443. I would suggest that the authors provide additional evidence, if possible, to confirm that the distances of the two SNRs are compatible with each other.

The manuscript presents the age estimates for G189 (30–100 kyr) and IC443 (3-30 kyr). These age ranges seem too broad to meaningfully constrain a time delay between explosions. Within such large ranges, nearly any sequence of explosion times is possible. A robust binary interpretation should require an explicit quantitative demonstration of the likelihood of the given delay from binary evolution models.
To that end, the authors do actually perform the binary population synthesis using the COMPAS code as supporting evidence, from which the time delay vs separation of SNRs predicted by the population synthesis of one million binaries are shown for two metallicities. Although interesting, it lacks quantitative estimation or a statistical test (even if it is implied, it is not presently clear), for example, the probability of obtaining the posited separation or delay time under a binary scenario versus a chance possibility. Without a statistical comparison, it is hard to substantiate a binary claim, even for future observations to look for.

Also, it is not clear to me, at least from the text itself, how a separation of 9 to 15 pc was posited? Can the authors make it clearer? Also, to this point, see point 4 below.

To summarize, the manuscript treats compatible distances and ages as proof of binary ancestry, but a statistical estimate needs to be shown for confirmation that this is not a chance spatial coincidence along the line of sight in an already complex region.

Response:

We agree that, for evolved supernova remnants, no single observational signature can unambiguously establish a binary progenitor origin. Our intent is therefore not to claim definitive proof, but rather to demonstrate that the observed properties of G189.6+3.3 and IC 443 are consistent with a binary-supernova scenario and difficult to reconcile by invoking a chance coincidence. Only a few supernova remnants are known to overlap in projection on the sky, and in all previously identified cases the remnants are located at substantially different distances. This represents the first candidate system whose observed properties are simultaneously consistent with a binary-supernova origin (lines 498-501 in the revised version). Additional details on this interpretation are provided in our response to Major Comment 2 from Reviewer #3.
Our study integrates well-established results from the literature with new intriguing observational evidence that collectively strengthens the case for this scenario.

The distance estimates for both remnants indeed carry non-negligible uncertainties, as is common for Galactic SNRs. For this reason, throughout the manuscript we

emphasize distance compatibility within uncertainties rather than identical distances inferred solely from extinction or absorption measurements. The optical study by Bakis et al 2024 identify hints for potential interaction between the two remnants. In addition to these methods, there is independent evidence suggesting physical association presented in our work (which are perhaps the strongest): IC 443 is well established in the literature to interact with the S249 HII region, and in this work we present evidence that G189.6+3.3 also interacts with the same source. In particular, we identify a radiative, decelerated shock at the northern boundary of G189.6+3.3 coincident with dense gas, where the gamma-ray emission shows a hadronic origin, in contrast to the predominantly leptonic emission elsewhere in the remnant. This is not a simple spatial correlation. The optical filament is detected in the UV and exhibits strong optical forbidden-line emission, with characteristic line ratios such as [S II]/Hα > 0.4–0.5, which are diagnostic of radiative shocks. This provides firm evidence that the filament (which is also confirmed to be of SNR origin in the literature) traces radiatively cooled SNR shock material. This strengthens the case that both remnants are located at compatible distances as they interact with the same dense structure.

We acknowledge that age estimates for evolved SNRs are intrinsically uncertain. Nevertheless, the quoted ranges for IC 443 (3–30 kyr) and G189.6+3.3 (30–100 kyr), derived from shock properties and multi-wavelength diagnostics reported in the literature, are typical and reasonably constraining for remnants. Rather than attempting to infer a unique explosion delay, we propagate these age uncertainties into a corresponding allowed range of delay times and test whether such delays are compatible with binary evolution expectations.

To this end, we use binary population synthesis with the COMPAS code to assess whether the inferred separations and delay times fall naturally within the distribution expected from binary supernova systems. The purpose of this analysis is not to compute a formal posterior probability for this specific system, but to demonstrate that the observed parameter space does not violate binary evolution predictions and arises naturally over a wide range of initial conditions. A meaningful quantitative constraint from the COMPAS population synthesis would require substantially tighter age estimates for both remnants than are currently achievable. For evolved SNRs such as IC 443 and G189.6+3.3, age determinations are intrinsically uncertain at the level of factors of a few, and any formal likelihood analysis would therefore be dominated by these uncertainties rather than by the underlying binary evolution physics. We also note that the percentages of the simulated systems falling within our system constraints are given in the manuscript.

We have revised the text to explain in more detail how the projected separation range of 9-15 pc is derived (lines 529-535 in the revised version)

Finally, in response to the referee's request for a quantitative assessment, we now include two complementary estimates of the probability of a chance angular coincidence (lines 586-649 in the revised version). Neglecting, for this purpose, the additional constraint of a common interaction with the S249 complex, we first estimate the chance probability that two unrelated SNR explosion sites would occur this close together on the sky by treating the remnants as point-like tracers of their explosion centroids; this centroid–centroid metric is the physically relevant one for assessing a possible common origin in a binary system. We then consider the complementary and more conservative estimate of the probability that two unrelated *extended* SNR shells overlap in projection, which explicitly accounts for their angular sizes. In both cases, requiring distance compatibility within conservative uncertainties based on absorption and extinction measurements further reduces the probability. While necessarily approximate, these estimates support our conclusion that a chance spatial coincidence is unlikely.

2. The multiwavelength modeling is presented well; however, the manuscript would benefit from including the key formulas used to derive the principal model parameters, such as the electron spectral break, maximum electron/proton energies, and related quantities, rather than only stating them briefly and citing external references. Providing these expressions directly in the text would make the analysis more transparent, easier to follow, reproducible, and more self-consistent for the reader.

The authors should show the interaction timescales for different interactions (pp, bremsstrahlung, synchrotron, IC, etc.), along with necessary dynamical timescales (for example, the system age, diffusion timescale, if relevant) for both regions, and discuss how that supports the separate emission from two zones.

The manuscript does not address the possibility of very-high-energy (VHE) gamma-ray detection in the >TeV range. If relevant data exist, the authors should include TeV-band overlays (e.g., from HAWC, MAGIC, VERITAS, or LHAASO WCDA) on their morphological figures to illustrate whether the GeV and TeV structures are spatially consistent. If not, the authors should discuss what future or current TeV instruments, such as CTAO, SWGO, and the LHAASO WCDA, are expected to observe from this region, if any, and whether such observations would serve the interpretations put forward in the manuscript.

Neutrino detection would provide definitive evidence for a hadronic origin of the gamma-ray emission from the northern region, as the manuscript correctly notes. Could the authors estimate the corresponding neutrino flux predicted by their hadronic model and compare it with the sensitivities of current and upcoming neutrino observatories? Such a comparison would help assess whether neutrino detection from this region is feasible and would further strengthen or challenge the proposed interpretation.

Response:

We agree that transparency and reproducibility are important. We thank the referee for this suggestion and the editor for the clarification. In the revised manuscript we now include the key expressions used to estimate the principal model parameters. These equations are now explicitly written in the text, together with a brief description of the quantities involved and the assumptions adopted in the calculation. Our intention is not to reproduce the full derivations available in the cited literature, but to provide sufficient information for the reader to reproduce the analysis without needing to consult external references. The relevant equations have been added in Section 4.5 (lines 1262-1291 of the revised manuscript).

We agree that comparing radiative and dynamical timescales can, in principle, be a useful diagnostic.
Comparing interaction timescales can be useful in situations where multiple radiative processes compete under similar physical conditions.
However, in the present case, the separation between the northern hadronic and southern leptonic emission zones is already implicitly and self-consistently established by the multiwavelength modeling itself. The adopted physical conditions in the two regions (in particular the gas density and magnetic field strength) directly determine which radiative channels can operate efficiently on the system age. In the dense northern region, the gamma-ray emission is successfully reproduced by hadronic interactions under the same conditions that independently indicate strong interaction with dense material, while a leptonic model would require electron populations that are inconsistent with the observed radio and X-ray emission. Conversely, in the southern region, the much lower ambient density renders hadronic and bremsstrahlung contributions inefficient on SNR timescales, and the observed emission is naturally explained by leptonic processes. These conclusions follow directly from the modeling assumptions and the results presented.
Providing a full set of interaction and dynamical timescales for each process and zone would therefore largely restate, in a different form, the same physical

constraints already encoded in the spectral fits, while substantially increasing the length and technical complexity of the manuscript.

We have now addressed both points in the revised manuscript. A discussion of existing very-high-energy (TeV) observations has been added, and the spectral energy distribution has been updated to include the available constraints from HAWC and LHAASO data. These measurements are discussed qualitatively, as their interpretation is currently limited by differences in the spatial models adopted in those analyses and by the morphology inferred from the eROSITA observations.

Regarding the neutrino emission, we have clarified in the manuscript that a quantitative neutrino flux estimate would be highly uncertain in this case. The gamma-ray spectrum of the northern region is strongly curved and dominated by GeV energies, implying that any neutrino flux estimate would depend critically on assumptions about the poorly constrained TeV tail of the particle distribution. As a result, such an estimate would be strongly model-dependent and would not provide a robust or testable prediction with current neutrino observatories.

Further details on the treatment of TeV observations and neutrino expectations are provided in our responses to Reviewer #1 (References point) and Reviewer #4 (minor point 6).

3. The proposed explanation that the northern gamma-ray component arises from proton re-acceleration within the H-alpha filament is interesting, yet somewhat incomplete in its current form. The manuscript invokes adiabatic compression and cosmic-ray re-acceleration, yet no physical modeling of compression diagnostics, possible proton spectral modifications, or cosmic-ray enhancement and gamma-ray luminosities is clearly presented.

The authors should also discuss the potential impact of ion–neutral damping and non-Bohmian diffusion within the cloud. These effects can significantly influence the overall gamma-ray emissivity and should therefore be addressed in the analysis.

Moreover, A simpler and well-established alternative of enhanced hadronic emission from shock-accelerated protons, which escape the shock front and interact with dense gas, remains entirely plausible. In fact, the hadronic modeling provided (at least seen from Table 3 and Figure 11) is more akin to DSA, rather than that of proton

reacceleration via adiabatic contraction. Given that proton re-acceleration is an important claim of the manuscript, the authors should provide some diagnostics as to why it is preferred over other acceleration mechanisms, accompanied by necessary quantitative modeling.

Response:

This level of modeling is beyond the scope of the present paper. A quantitative treatment of adiabatic compression and cosmic-ray re-acceleration would require additional, poorly constrained inputs, such as the pre-shock and post-shock densities, the detailed shock structure, and the spatial distribution of the interacting gas. In the absence of such information, any calculation of compression factors, spectral modifications, or cosmic-ray enhancement would not be meaningful.
The key observational elements motivating this interpretation are the presence of H-alpha filaments, together with their associated UV emission and optical forbidden-line diagnostics, which indicate radiative shocks and evidence for interaction with dense gas; and the spatially coincident with the radiative region gamma-ray emission exhibiting a hadronic spectral signature. Under such conditions, the gamma-ray emission cannot be explained by freshly accelerated particles. Compression of pre-existing cosmic rays naturally increases both their normalization and effective energy density, leading to enhanced hadronic gamma-ray emission, while only modestly modifying the spectral shape. Our modeling therefore focuses on reproducing the observed gamma-ray spectrum under these physical conditions, rather than on predicting detailed spectral evolution from first principles.

The discussion requested on ion–neutral damping and non-Bohmian diffusion within the cloud has been added (lines 431-451 in the revised version).

We believe that this point is discussed in the manuscript, and we have now further clarified that a standard DSA scenario cannot be ruled out. This is also why the term "(re-)acceleration" is used in the title and elsewhere in the text, to emphasize that the proposed scenario does not exclude standard diffusive shock acceleration. While the gamma-ray spectrum alone cannot uniquely distinguish between these mechanisms, the spatial morphology provides an important diagnostic. In the northern region, the gamma-ray emission is confined and spatially coincident with Hα filaments, UV emission, and optical forbidden lines tracing radiative shocks, as well as with dense shocked gas. This morphology favors local gamma-ray production at the radiative shock interface over an illumination scenario involving

runaway cosmic rays interacting with more distant clouds; essentially gamma-ray emission observed ahead of the shock and correlated with external clouds. In scenarios where shock-accelerated protons escape upstream and illuminate nearby dense gas, the gamma-ray emission is expected to be offset from the shock front. Relevant discussion has been added to the text (mainly lines 383-400 in the revised version). In summary, the environment motivates the investigation of specific particle acceleration mechanisms. "DSA" has been tested for both components.

4. The manuscript is extremely long, verbose, and difficult to navigate. The manuscript repeatedly re-states similar arguments in multiple sections. The narrative would benefit from substantial condensation and clearer presentation of core findings and necessary quantitative estimations. It would be advisable to soften the core claims and avoid overstating the conclusions. Moreover, the authors should discuss the caveats of their approach in a dedicated section, and they should also comment on the specific observational and theoretical developments that could help confirm, refine, or challenge their interpretation in the future.

Response:

Some degree of repetition between the Results and Discussion sections is intrinsic to the Nature article format, in which key findings are first presented and then interpreted in a broader context. That said, we have carefully reviewed the entire manuscript and implemented focused revisions in places where repetition could be reduced and the presentation streamlined (mainly in sections 1,2,3, but also in the methods section where necessary). Results (section 2) has been restructured to improve clarity and avoid repetitions. Bold run-in headings are used in the discussion to guide the reader.
In the revised manuscript, and after taking into account all four referee reports, we have incorporated the relevant caveats directly where they are most pertinent, primarily within the Discussion section, rather than repeating them in a dedicated caveats section. We find this approach more effective, as it avoids reiterating material already discussed and allows the caveats to be addressed in context, together with the arguments explaining why they do not undermine the main conclusions of the paper.

Minor comments:

"1. The eROSITA observations indicate that the X-ray shell is dominated by thermal emission. However, the modeling yields a magnetic field of ~270 microG in the northern region. Could the authors comment on whether any non-thermal X-ray synchrotron component is expected under such a strong field, and how does that compare with the thermal X-ray flux inferred from the eROSITA data?"

Response:

Relevant discussion has been added in section 4.5 (lines 1217-1240 in the revised version).

2. The derived magnetic field in the southern region (~5 microG) is close to the Galactic average. Such a low field implies long synchrotron cooling times and may allow high-energy electrons to escape efficiently from the emission zone. Could the authors discuss if and how electron escape is expected to change their spatial modeling of the southern region?

Response:

While the relatively low magnetic field inferred for the southern region implies long synchrotron cooling times and could in principle allow for more efficient electron escape, there is no observational indication that such escape significantly affects the spatial distribution of the gamma-ray emission. The southern gamma-ray component observed by Fermi-LAT is spatially extended and correlated with the X-ray shell, rather than exhibiting a diffuse or displaced morphology that would be expected if electron escape were dominant. Within the angular resolution and energy range of the current data, the adopted spatial template therefore provides an adequate description of the emission, and the inclusion of additional transport effects is not required.
We have added a short discussion in section 4.5 (lines 1300-1310 in the revised version).

3. For the southern region, what mechanism is assumed for proton acceleration? Is it standard diffusive shock acceleration at the SNR forward shock, and if so, why not use the same in the northern region? To reiterate, clarifying the physical distinction between the two acceleration mechanisms in two separate environments for the same species would strengthen the interpretation.

Response:

Spectral compatibility with DSA does not imply that fresh DSA is physically efficient in all environments.
For the southern region, we assume a hard proton (and electron) injection index of approximately 2.0, which is fully consistent with standard diffusive shock acceleration (clearly stated at the paper) at a non-radiative forward shock propagating into relatively low-density material. We therefore interpret the southern spectral component as compatible with standard DSA.
In the northern region, we also explicitly tested proton spectra with injection indices of both 2.0 and 2.3, which can explain the spectrum. However, the physical environment in the northern region differs substantially from that in the south. The interaction with dense material associated with the S249 HII region implies a strongly decelerated and radiative shock, conditions under which fresh diffusive shock acceleration is expected to be inefficient. In such environments, compression and re-acceleration of pre-existing cosmic rays can dominate the non-thermal emission. Also for the northern region we obtain a low energy cutoff with respect to the southern component can be due to the recent interaction of the SNR with the dense material of the S249 HII region.
So, the distinction on the mechanism for proton acceleration between the two regions therefore does not arise from the spectral modeling alone, but from the combination of spectral results with environmental and morphological information. While a DSA origin for the northern emission cannot be ruled out, the local shock conditions and spatial coincidence with radiative shock tracers favor a re-acceleration (crushed-cloud) interpretation.
In the southern region, we assume DSA at the forward shock, which propagates into relatively low-density material and remains non-radiative. This interpretation is consistent with the inferred shock properties and with the leptonic-dominated gamma-ray emission observed from this region.
In the northern region, we explicitly test both standard DSA (section 4.5) and proton re-acceleration in radiatively cooled, compressed gas (the crushed-cloud scenario; section 4.6).
We have made this clearer in section 4.5 (lines 1331-1348 in the revised version).

4. The northern gamma-ray emission is modeled using a 2D Gaussian template, while the southern region is described using the eROSITA-based polygon corresponding to the full SNR shell. Could the authors clarify the physical and geometric motivation for this transition? For example, is the Gaussian intended to represent the molecular cloud interacting with the shock, while the polygon traces the broader SNR structure? Explicitly defining where the two components separate spatially would improve the clarity of the morphological analysis.

Response:

The use of two different spatial templates reflects the distinct physical origins and morphologies of the gamma-ray emission in the northern and southern regions. The southern component is modeled using a polygonal template derived from the eROSITA X-ray image, which traces the full extent of the supernova remnant shell and represents gamma-ray emission associated with the large-scale shock structure.
In contrast, the northern gamma-ray emission is spatially confined (appearing as a blob) and coincident with a narrow Hα filament tracing the interaction with dense material associated with the S249 HII region. As part of the revised manuscript (addressing major comment 3 from reviewer 4#), we now explicitly show both best fit spatial templates in the right panel of Fig. 2. We initially tested the Hα filament itself as a spatial template for the northern emission; however, its narrow width is not well matched to the angular resolution of the Fermi-LAT data. So, it could not model the entire hadronic blob. We therefore explored a range of regular geometrical templates and found that a two-dimensional Gaussian, with its position and extension allowed to vary freely, provides the best description of the data and effectively traces the location of the Hα filament. This motivates our discussion as well regarding the re-acceleration.
Overall, the southern spatial template is driven by the global geometry of the SNR shell, whereas the northern component is motivated by the presence of the Hα filament tracing the localized interaction with dense gas. This is now more clearly stated in the paper in the morphological analysis section.
The spatial separation between the two components is explicitly defined by these templates and is now illustrated in Fig. 2, improving the clarity of the morphological decomposition.

Reviewer 3#:

Summary:
This work reports on an observational study of a newly detected Galactic supernova remnant (SNR) in gamma-rays using Fermi-LAT, which claims new discoveries as follows: (1) the SNR shows a north-south segregation in the character of its gamma-ray spectra, for which the authors claim to be the first ever case found to display a hadronic vs leptonic nature of the emission inside a single SNR; (2) the authors claim that this SNR is originally a binary companion to the progenitor of the famous IC 443 in the same region based on sky proximity, distance estimates and binary population synthesis result; (3) they also use a spatial correlation between H-alpha emission with the newly found GeV gamma-rays in the northern part to support its hadronic origin, in particular through the "crush-cloud" mechanism where re-accelerated Galactic cosmic rays compressed inside a radiatively cooled region behind the SNR shock is responsible for the high-energy radiation.

Main comments:

1) On discovery #1 listed above - I am sure the authors are aware of other systems which are known to show (or at least have shown strong hints on) such a mixed contribution from both hadronic and leptonic gamma-rays. One might list Vela Junior, SN1006, RX J1713, CTB 109, etc. It would be an over-statement to say this work encompasses the first discovery of such a mixed emission mechanism. Along the same line, the approach of using spatial correlation of gas distributions with gamma-rays for the identification of gamma-ray emission mechanism is not new and has been used in other works by people like H. Sano, Y. Fukui and co-workers for years, arguably in a much more sophisticated and quantitative way than what the authors have done here. Further support for the novelty of this work to claim such a first discovery is in place, or else I would suggest the authors to tone down their claim and acknowledge similar previously found results.

Response:

We find this comment understandable. The issue raised is primarily one of perception and wording, rather than of missing or incorrect science.

We do not claim that this work represents the first discovery of an SNR exhibiting mixed leptonic and hadronic gamma-ray emission. Such a claim would indeed be incorrect. In fact, we already explicitly acknowledge the existence of other SNRs showing mixed leptonic and hadronic gamma-ray emission in the third paragraph of the Discussion section (original manuscript), including well-known cases such as SN 1006. In the same paragraph, we also state what distinguishes the present system from these previously studied remnants.

The key novelty of this finding lies not in the coexistence of leptonic and hadronic gamma-ray emission, but in the clear spatial segregation of the two emission mechanisms within a single SNR, which can be modeled using two distinct spatial components and confidently interpreted as one region dominated by hadronic emission and another dominated by leptonic emission.

Among the mixed leptonic/hadronic gamma-ray-emitting SNRs, SN 1006 comes closest to such an interpretation, with one limb clearly dominated by leptonic emission. However, in the opposite limb, the gamma-ray emission is still best described as having significant contributions from both leptonic and hadronic components, and the authors explicitly state that a strong leptonic component remains present but now hadronic emission becomes more important compared to the other limb. Thus, a clean spatial separation between dominantly leptonic and dominantly hadronic regions is not achieved.

For a few other rare young TeV SNRs (e.g., RX J1713.7–3946 and Vela Jr.) which show mixed hadronic and leptonic emission, the origin of the gamma-ray emission remains heavily debated. The gamma-ray data in these systems generally still allow either leptonic or hadronic scenarios, or hybrid models, without yielding a uniquely compelling decomposition into spatially distinct emission mechanisms.

Furthermore, while mixed leptonic–hadronic gamma-ray emission is commonly inferred in SNRs interacting with dense material, such regions typically retain a substantial co-spatial leptonic contribution. In contrast, the northern region of G189.6+3.3 shows little evidence for a co-spatial leptonic gamma-ray component, suggesting unusually efficient suppression of electron-driven gamma-ray emission or enhanced proton dominance in this region. This behavior, combined with the presence of a clearly leptonic-dominated region elsewhere in the same remnant, makes G189.6+3.3 a particularly distinctive case.

We have rephrased the relevant parts (mainly lines 312-346 but also elsewhere, e.g., abstract) of the manuscript to clarify this distinction more explicitly and to avoid any possible overstatement of the novelty of our results, while ensuring that similar previously studied systems are properly acknowledged.

2) On discovery #2 - while providing circumstantial supports such as distance estimates and a viability test through population synthesis is highly appreciated, but as indicated in the paper title which puts it as "the first observed binary system's aftermath" so that it is actually pointing toward a solid confirmation, it is again risking the danger of an over-statement. Claiming a companion origin of this SNR with IC 443 in particular needs a smoking gun evidence. Is there any observational characteristics of this SNR discovered which uniquely proves that it originated from a binary progenitor system? Any evidence of envelope stripping from the SNR dynamics and emission? etc. It is intriguing indeed that this SNR does look to be related to IC 443 in some way, but within the context of this work in its current state, I worry that it would be premature to over-interpret the findings as a discovery. ‘

Response:

We would like to clarify that we do not claim a definitive or unambiguous confirmation that G189.6+3.3 originated from a binary progenitor system, nor do we present any single “smoking gun” observational signature that would uniquely prove such an origin. We fully acknowledge that no such direct evidence is currently available.
In fact, the answer to the general question “Is there any observational characteristic that uniquely proves a binary progenitor?” is no. While theoretically conceivable, such scenarios are instead evaluated through consistency with multiple observational constraints and population-level expectations.
Our intention is instead to demonstrate that the observational properties of G189.6+3.3 are fully consistent with, and indeed support, a binary progenitor scenario when considered in the broader astrophysical context. Only a few supernova remnants are known to overlap in projection on the sky, and in all previously identified cases the remnants are located at substantially different distances. This represents the first candidate system whose observed properties are simultaneously consistent with a binary-supernova origin (lines 498-501 in the revised version). Our study integrates well-established results from the literature with new intriguing observational evidence that collectively strengthens the case for this scenario.

Specifically, (i) G189.6+3.3 and IC 443 are the only known overlapping SNRs in this region of the sky, (ii) we establish that they are located at the same distance, and (iii) their inferred ages, angular separation, energetics, and environments do not violate

any expectations for a common binary origin. Given that the majority of massive stars are found in binary or multiple systems, such a scenario is therefore plausible and well motivated. The massive progenitor and jet-like feature further add to this dynamical or geometry imprint of this scenario. Even clear evidence for envelope stripping in the ejecta would be suggestive for a binary origin and not a direct proof. While evidence for a stripped-envelope progenitor - such as reduced hydrogen and helium content - would be consistent with a binary evolution scenario, such signatures are not unique, as similar observational characteristics can also arise from single-star Wolf–Rayet progenitors and may be further modified by late-time mixing and environmental interactions during the SNR phase. In fact, the eROSITA spectroscopy indicates an ejecta-enriched plasma (supersolar O/Ne/Mg/Si and subsolar Fe), consistent with a stripped-envelope core-collapse origin. This is the kind of evidence one would expect if the progenitor lost much of its H envelope. But it does not provide a unique diagnostic of envelope stripping or a binary progenitor channel.

The binary population synthesis simulations presented in this work are not intended to provide direct proof, but rather to test the viability of a binary-origin hypothesis under realistic assumptions. These simulations demonstrate that a binary system capable of producing two SNRs with the observed properties is not only possible, but statistically reasonable.

For this reason, we have deliberately phrased our conclusions conservatively.

While the title highlights the broader implication of our findings, the Abstract and Discussion explicitly clarify that the presented evidence supports and bolsters a binary progenitor hypothesis rather than providing definitive confirmation. We also outline avenues for strengthening this interpretation with future observational evidence, including constraints on the progenitor environment such as metallicity, and related compact objects properties (when detected). We believe that this balance between interpretive framing in the title and scientific caution in the text follows common practice in Nature.

We have also expanded the discussion of the X-ray properties of G189.6+3.3, noting that the eROSITA spectral analysis reveals ejecta-enriched plasma consistent with a stripped-envelope core-collapse explosion; however, we emphasize that such signatures provide supportive evidence only and do not uniquely establish envelope stripping via binary interaction. We further emphasize that while a single, definitive observational signature is unlikely to be found, the convergence of independent lines of evidence consistently points toward this interpretation (e.g., lines 554-570 in the revised version).

In response to the first main point raised by Referee#2, we have also added a quantitative statistical estimate to place the proposed association in a broader probabilistic context.

3) On discovery #3 - the usual explanation of H-alpha filaments is from a fast SNR shock running into a partially neutral medium which triggers charge exchange interactions, so that the filamentary look in H-alpha is coming from the post-shock ionization length scale. This work claims otherwise that the shock has slowed down to enter the radiative regime so that post-shock compression inside the cooling gas (the filament) is responsible for the enhanced GeV emission in the context of the so-called "crushed cloud scenario". Especially in a situation without any radio spectrum or other signatures of radiative shocks such as optical forbidden lines or OH masers to back up, it is unclear to me how the authors can pin down the emission mechanism using one-zone models for gamma-ray-only spectra with a high confidence.

Response:

We acknowledge that this argument may not have been sufficiently supported in the original version of the manuscript. We have now expanded the discussion and incorporated additional multiwavelength evidence that supports the interpretation of radiative shocks.
Optical spectroscopy of the filament reveals strong forbidden-line emission, including N II, S II, O I, and O III, with elevated S II to H alpha ratios in the range of approximately 0.5 to 1.4 (see Bakis et al 2024). Such values exceed the standard threshold for shock excitation and are characteristic of radiative shock conditions, where post-shock gas undergoes efficient cooling and compression. These optical line diagnostics rule out a purely Balmer-dominated, non-radiative shock interpretation for the filaments.
In addition, the filament is bright in the ultraviolet. UV-emitting filaments are commonly observed in evolved supernova remnants in which shocks have slowed and entered the radiative regime. When considered together with the strong optical forbidden-line emission and elevated S II to H alpha ratios, the UV emission provides independent and complementary support for the presence of radiative, cooling shocks.
Taken together, this multiwavelength evidence demonstrates that the filaments trace radiative post-shock regions, providing the physical conditions - namely strong compression and enhanced target density - required for the radiative-compression ("crushed cloud") scenario. We have revised the manuscript accordingly (several parts of the papers, e.g., 452-477, 231-243, 410-430 in the revised version) to clarify

that this interpretation is not based on gamma-ray spectral modelling alone, but is instead supported by independent optical and ultraviolet shock diagnostics.

4) Age estimates - how can one estimates the age of the SNR using an assumption of Sedov-phase evolution while simultaneously employing the "crushed cloud" scenario for the gamma-ray emission which requires the SNR shock to have evolved into the radiative phase for a substantial period? Is the use of a Sedov-like discussion applied in an averaged sense for the SNR as a whole? If so, how can one justify this without a separated estimation of dynamical ages between N and S which are apparently very different e.g. from the spectral model findings? Please clarify.

Response:

In the manuscript we estimate the remnant age using two complementary approaches: (i) the standard Sedov–Taylor scaling relations applied to representative global remnant properties, and (ii) the Leahy (2017) SNR evolution calculator, which provides consistent estimates of age and evolutionary stage (including the onset of radiative cooling). We use these two methods (+a third method for an upper limit estimate) as cross-checks to ensure that the inferred age and characteristic shock conditions are not sensitive to a single implementation.
We emphasize that both the Sedov–Taylor relations and the Leahy (2017) calculator necessarily provide global, averaged estimates for the SNR as a whole and do not imply that all sectors of the remnant must be in the same dynamical phase. In an inhomogeneous environment, shocks propagating into dense regions can decelerate rapidly and enter the radiative regime locally, while other parts of the shell expanding into lower-density material may remain approximately adiabatic and be described, to first order, by Sedov-like evolution. Accordingly, the radiative-compression ("crushed cloud") scenario is invoked only for the northern interaction region, whereas the Sedov-based age estimate is intended to characterize the remnant globally.
We clarify that the quantity relevant for our binary interpretation is the time since explosion for each remnant, because this is what constrains the time delay between the two supernovae. In that sense, sector-dependent dynamical states (e.g. a locally radiative shock in the north versus more adiabatic expansion elsewhere) do not imply different explosion times; they reflect local variations in ambient density and cooling efficiency within a single remnant of a single age. For this reason, we adopt a

global age estimate for the SNR as a whole rather than attempting to assign separate "dynamical ages" to the northern and southern regions.
We have added relevant clarifications in section 4.4.2.

Minor comments:

1) On the crushed cloud model - some major references are missing, especially the more dedicated numerical works following up Uchiyama et al. (2010).

Response:

We have added key follow-up references to the crushed-cloud/radiative-shell re-acceleration scenario that expand upon Uchiyama et al. (2010), including dedicated modelling efforts by Tang & Chevalier (2014), Lee et al. (2015), and Cardillo, Amato & Blasi (2016).

2) On the energetics estimation for gamma-ray emitting CRs - since there are large uncertainties involved in the ambient environments in the spectral models, it is more informative to provide numbers for the energetics in a way scalable with target gas density etc.

Response:

We agree and have revised the text to present the CR energetics in a scalable form, explicitly indicating the dependence on the assumed target gas density (lines 1331-1348 in the revised version).

3) There is a mentioning of the "cooling break" for the electrons, but it is not clear to me how its estimation influences the spectral modeling. The necessity of introducing such a break is also unclear. The notion of "cooling break" arises from the argument of a balance between cooling and dynamical (or particle injection) timescales. It's actual appearance in the spectra is usually exaggerated by one-zone models with a single particle population and a constant B-field ignoring the spatial variation of both and time evolution of the shock and post-shock gas. In other words, the cooling time scales and so on are usually both time and spatial dependent. The "break" in reality is hence much more smeared out

when one volume-integrates the electron spectra over the whole remnant at any given age, such as in a hydrodynamic simulation.

Response:

We essentially follow the methodology described in Devin et al 2020. Using a cooling break in a one-zone model is a standard modeling assumption. Its physical interpretation should be phenomenological, and it does not imply a sharply observable spectral feature if cooling is weak. When cooling is negligible, the break effectively disappears in the fit. Its location is estimated by equating the synchrotron cooling timescale with the age of the remnant, again following the same approach adopted in previous broadband SED studies (e.g. Devin et al. 2020). While the break can influence the model internally (e.g. the relative normalization and slope of the high-energy synchrotron component), it is not used as a physical diagnostic, and our conclusions do not hinge on its precise position or sharpness. We have now clarified this in section 4.5 (page 28 in the revised version).

4) Table 3 - given the very high uncertainties, it would be more helpful to also provide the "allowable" ranges of the key parameters, rather than only printing the fitting values from the few models being tried out.

Response:

The quoted parameters are representative values (this is now more clearly stated in line 1312 in the revised version) obtained from the naima SED modeling constrained by radio data, X-ray upper limits, and gamma-ray measurements. We are performing a forward modeling/parameter exploration not a statistical fitting with sampling. The parameters are model-derived but not statistically fitted with errors. We preferred to inspect manually a reasonable range of values. Leaving free the fit with so few MWL constraints is often risky, leading to divergence. The reported values are therefore intended to be informative, demonstrating that the observed emission can be reproduced with physically reasonable parameters, rather than representing best-fit values with associated errors. Most importantly, in the absence of more constraining radio and X-ray measurements beyond upper limits, the parameter values can become highly degenerate, making it difficult to define a broader allowable range.

Reviewer 4#:

This paper details the observation and spectral analysis of the supernova remnant G189.6+3.3 in the region of the well known SNR IC 443 and the HII region S249. The authors analyse data from the Fermi-LAT telescope, combining this with multi-wavelength data to model particle acceleration in this SNR. From this data the authors come to two important conclusions, that the northern region of the SNR is dominated by hadronic emission of gamma-rays and that combined with the spatial correlation with the S249 this indicative of an interaction with the HII region. The authors then propose the the two SNRs in this region originate from high mass star binary system and derive a range of explosion times from these two progenitor objects.

Generally speaking this is a well written paper, with the data analysis and interpretation being of a high standard with the authors going to significant efforts to back up their suggestion that G189.6+3.3 and S249 lie at the same distance. There are a few areas where some more explanation and justification could be provide to better demonstrate that both IC 433 and G189.6+3.3 are located at the same distance and hence originate from binary progenitors.

I will split my comments into more major or general comments, followed by minor comments, typos etc.

General comments:

When reading some areas of this paper some of the flow of the paper can be lost by the sentence structures used. For example:
Ln 75 “Follow up studies at higher energies, that is X-rays, have resulted…” or
Ln 86 “gamma-ray emission, given that relativistic hadrons only radiate…, above the energy threshold…”
I would ask the authors to take a read through the introduction and check to the flow of the text and perhaps use a more simple sentence structure e.g. “Follow up studies at X-ray energies have resulted ”.

Response:

We agree that, in places, the flow of the text can be improved by simplifying sentence structure and wording.
In response, we have carefully re-read the Introduction and revised several sentences to improve clarity and readability, adopting simpler constructions where appropriate (including the examples pointed out by the referee). In conjunction with major comment 4 from Referee 2#, we have streamlined the manuscript and improved long and verbose sentences throughout.

The authors invest significant effort in showing that the the distances of the SNR G189.6+3.3 and the HII region s249 are at a compatible difference with one another. These arguments combined with the interaction increased brightness in the s249 region and hadronic emission is generally quite convincing. However, it is not clear from either the introduction or the work presented in the papers is how strong the correlation is between IC443 and the HII region. I think it is important to discuss the strength of this evidence as it is equally at important to support the conclusion of a binary system.
For example reference 58 in the paper suggests that s249 is located 600 pc in front of IC 443, this should be discussed.

Response:

We have clarified this issue in the manuscript and expanded the discussion of the evidence linking IC 443 and S249.
The distance difference quoted in Ref. 58 (approximately 1.9 kpc for IC 443 and approximately 1.3 kpc for S249) is based on kinematic distances inferred from very small differences in systemic radial velocities (about 6–9 km/s), assuming a Galactic circular rotation curve. As reported in that work, the velocity component attributed to S249 is ~6 km/s, while the systemic velocity of IC 443 is ~9 km/s. Near the Galactic anti-centre, a given Δv does not reliably map to a well-determined Δd. Kinematic distances in this direction are intrinsically poorly constrained and highly sensitive to non-circular motions and projection effects. Indeed, the authors of Ref. 58 explicitly note the limitations introduced by projection effects when interpreting the kinematics. The literature explicitly compares the kinematics of IC 443 and S249 noting that assigning a unique systemic velocity to S249 is non-trivial (multiple components along the sightline), and that projection/geometry effects complicate interpretation (i.e., the kinematic argument alone is not definitive).
Moreover, the distance estimate for S249 in Ref. 58 relies on identifying a velocity component at ~6 km/s, described as being common across the entire field, as the systemic velocity of the HII region. This necessarily involves a model-dependent

assumption in a line of sight that contains multiple kinematic components. The reported line width (FWHM ~ 20 km/s) is relatively broad for a single quiescent HII region and likely reflects unresolved structure or multiple overlapping components, further reducing the precision with which a unique systemic velocity can be defined. We therefore regard the resulting rotation-curve distance for S249 as uncertain at the several-hundred-parsec level.

For these reasons, we do not consider that the kinematic distance quoted in Ref. 58 can rule out a common physical distance for IC 443 and S249. Instead, we interpret it as illustrating the limitations of rotation-curve–based distances towards the Galactic anti-center.

Our interpretation of a common environment is further supported by the strong morphological correspondence between the northeastern rim of IC 443 and the structure of the S249 HII region, which goes beyond a simple line-of-sight coincidence and suggests interaction with the same large-scale dense medium. In addition, we derive an independent distance estimate for G189.6+3.3 that is fully compatible with that of IC 443. A common environment for both SNRs is also supported by the CO velocity-integrated maps, which show that the CO structures associated with both IC 443 and G189.6+3.3 are found in compatible velocity ranges and therefore compatible distances (see also our response to the first Suggested Improvement from Reviewer #1). We emphasize that the distance compatibility has also been investigated through previously reported observational indications, such as the presence of small filaments in the southeast region of IC 443 that one can interpret as possible signs of interaction between the two SNRs (Lee et al 2008), as well as the roughly compatible systemic velocities of an H I structure that seemingly traces part of the G189.6+3.3 shell and the H I structures associated with IC 443 (Bakis et al 2024); see lines 143–145 and 310–315 of the original manuscript. Although both of these arguments are speculative in nature and do not constitute strong evidence for a physical interaction, they add additional context to the discussion.

Our work therefore provides new and stronger support for a common distance among the components of the IC 443 complex, based on their shared large-scale environment and with a particular focus on G189.6+3.3, which has not been previously explored in this context.

Relevant discussion has been added to the manuscript (mainly lines 1069-1096 & 481-488 in the revised version).

I think it would be much clearer to understand the analysis if there was a plot included showing all the best fit spatial models on a TS map (or add this to an already existing map), as at the moment it is not clear exactly which region is where. This is particularly

important as many different models are used. It is, for example, not clear to me exactly what regions are used for the extraction of the northern and southern regions, this needs to be made clearer. Also calling these north and south may be a little confusing, because if I understand correctly they are overlapping.

Response:

We agree that clearly visualizing the spatial models used in the analysis is important, particularly given the number of models explored. This point is partially addressed in the right panel of Fig. 2, where we show the eROSITA polygon together with the Hα filament, which are the two spatial templates most relevant to the discussion of the best-fitting model.
As discussed in the text, the Hα filament is interpreted as tracing the origin of the hadronic gamma-ray emission. However, its very narrow morphology does not allow it to be used directly as a spatial template to describe the full extent of the hadronic component. This test has been done and it is included between the different spatial templates tried. For this reason, we replace the Hα filament with a two-dimensional Gaussian model, whose position and extent are fitted. The best-fit Gaussian is centered on the Hα filament, indicating a close spatial correspondence between the two. This relationship is described in the text (see lines 209–213, 316–318, and more explicitly 320–324 - original version of the manuscript) and is implicit in Fig. 2, which shows the relative locations of the eROSITA polygon and the Hα filament.
We considered adding an explicit overlay of the best-fit Gaussian to the same panel. To avoid redundancy and excessive visual complexity, we initially chose to clarify this correspondence in the text instead. Following the referee's suggestion, we have now added the position and spatial extent of the best-fit Gaussian overlaid on the Hα filament in the right panel of Fig. 2, making the spatial relationship between the templates immediately clear.
Regarding the terminology of "northern" and "southern" components, we acknowledge that these regions are not strictly disjoint and partially overlap. We have now clarified this point in the manuscript and emphasize that these labels are used as descriptive identifiers rather than to imply fully separate spatial regions (see section 4.4.2., mainly lines 867-878 in the revised version).
We believe these additions and clarifications improve the readability of the analysis and make the spatial modelling choices clearer to the reader.

Minor comments:

“Ln 82: And -> and”

Response:

Starting a new sentence with “And” is grammatically acceptable; however, we acknowledge that the referee may dislike this stylistic choice. To avoid unnecessarily lengthening the sentence, we have instead rephrased it using “By contrast,” (lines 83-84 in the revised version).

“Ln 100: is the G189… -> is G189…”

Response:

Adopted (line 102 in the revised version).

“Ln 122: it may be worth mentioning why this source is no longer considered linked”

Response:

We have clarified the text to avoid any ambiguity. In Ref. [10], the origin of the extended GeV emission (FGES J0619.6+2229) was mentioned only briefly in a speculative context, noting that it might be unrelated to IC 443 and possibly associated with the nearby SNR candidate G189.6+3.3 due to the presence of a second source in the field; however, no association was claimed. In Ref. [12], the source was updated to 2FGES J0618.3+2227 using a longer LAT dataset, and no association with Galactic SNRs was reported. Consequently, this GeV source was not previously linked to G189.6+3.3. We have revised the wording accordingly to reflect this more accurately (lines 119-128 in the revised version).

“Ln 167: is it possible to shorten gigaelectronvolt to GeV after the first usage? Or is this a journal requirement?”

Response:

Energy units like GeV, TeV do not need to be written out when used as adjectives or symbols, even without numbers, once defined. So, we adopted the suggestion and corrected throughout.

"Ln 174: I think the eROSITA paper should be referenced here"

Response:

We note that the eROSITA paper is based on a different dataset and analysis. In this work, we use eROSITA DR1 data and perform an independent analysis from scratch. As such, we do not directly rely on results from that study (such as maps), and therefore we did not include it as a reference at this point in the text.

"Ln 202: Is it really an excellent candidate to be a significant neutrino emitter, although seemingly hadronic dominated it is also distant and not so bright. Was an expecting neutrino flux calculated?"

Response:

Our comment is in the context of neutrino targets from this source class. SNRs are established hadronic accelerators based on gamma-ray spectra with the π^0-decay feature. Neutrinos are the only direct messenger of hadronic interactions that has not yet been detected from individual SNRs. So conceptually, neutrinos are the missing smoking gun that would unambiguously confirm ongoing hadronic acceleration. The distance of ~1.5-2.0 kpc places this source among relatively nearby Galactic remnants. In general, good Galactic neutrino targets are preferentially nearby (within ≲2 kpc). It is true that the gamma-ray flux of the hadronic component remains modest compared to brighter gamma-ray SNRs such as IC 443; however, none show such a clear spatial separation of a dominant hadronic component. Our point is that SNRs with dominant hadronic gamma-ray emission constitute the most relevant class of Galactic SNRs for neutrino studies, as both gamma rays and neutrinos are produced via pion decays. The lack of a co-located strong leptonic component diluting the signal is crucial. The ability to spatially disentangle a dominant hadronic component makes this target particularly valuable. Identifying such systems is therefore important not only for individual source studies, but also for population studies and for constraining neutrino production scenarios, even when individual sources may lie below current neutrino detection thresholds. However, we acknowledge that there exist significantly brighter Galactic remnants and are therefore likely to be investigated first. This is now more clearly conveyed in the paper (see lines 217-230 in the revised version).

We did not attempt to calculate an expected neutrino flux for this source. While neutrino emission is intrinsically linked to hadronic gamma-ray production, a reliable flux estimate would require detailed modeling of the parent cosmic-ray spectrum, maximum acceleration energy, particle escape and diffusion, and the spatial distribution of target material. This information is not available.
Moreover, estimating the neutrino flux directly from the GeV gamma-ray flux could significantly overestimate the true neutrino flux if the source spectrum cuts off and does not extend into the TeV regime (as in our case). Given the curved and GeV-dominated gamma-ray spectrum of the northern region, any neutrino flux estimate would be dominated by assumptions about the poorly constrained TeV tail of the particle distribution. As a result, such an estimate would be highly model-dependent and uncertain, and would not provide a robust or testable prediction with current neutrino observatories. Given the associated uncertainties and the fact that no individual Galactic SNR has yet been detected in neutrinos, we consider such a calculation to be beyond the scope of this work and not to add meaningful results.

"Ln 256: "giving birth" -> producing"

Response:

Adopted (line 285 in the revised version).

"Ln 274: I'm not sure how this is mimicking an IC peak, is it not the interpretation that this is actually an IC peak?"

Response:

We have revised the text (lines 303-305 in the revised version) to make clear that the observed spectral shape is interpreted as being consistent with an inverse-Compton–dominated component, rather than using the rather ambiguous wording "mimicking an IC peak."

"Ln 294: I'm not sure triggers is the right word here, it acts a a target for hadrons"

Response:

Rephrased for clarity (lines 334-341 in the revised version).

"Ln 327: extremely well localised is a bit difficult to interpret without any numbers, how good is the positional localisation?"

Response:

We have quantified the positional localization by explicitly stating the typical LAT angular resolution at energies above 10 GeV (lines 376-378 in the revised version).

“Ln 370: It is not very well explained what the COMPAS code actually does and what is simulated here, it is simply stated that binary systems were simulated, more explanation would be needed”

Response:

We have expanded the text to briefly explain what the COMPAS code simulates and which physical quantities are extracted for our analysis (lines 512-522 in the revised version).

“Ln 460: remove “sophisticated”, it is just a python interface”

Response:

Adopted (line 739 in the revised version).

“Section 4.2.1.: I’m not sure why there is a split here to talk about eROSITA between LAT analysis sections, also this section is more a description of the data and not morphological analysis”

Response:

Section 4.2.1 is intentionally placed here because it provides the eROSITA imaging analysis that underpins the subsequent gamma-ray morphological modeling. In this section, we describe the construction of the eROSITA maps that are later used as spatial templates in the LAT morphological analysis. While the section primarily describes the data processing and map production rather than a standalone morphological interpretation, it is included at this point to maintain a clear logical flow from the preparation of spatial templates to their application in the LAT analysis. We have clarified this motivation in the text (lines 761-763 in the revised version).

“Ln 514: employed -> included?”

Response:

Adopted (lines 797-798 in the revised version).

"Ln 515: this sentence is a bit confusing please consider restructuring"

Response:

Adopted (lines 798-800 in the revised version).

"Ln 526: PS map needs explanation here at first usage, it is not a map that many Fermi users are familiar with"

Response:

We have moved the definition and explanation of the PS maps to their first occurrence in the manuscript (lines 816-819 in the revised version).

"Ln 667: Was the morphology of the fitted 2d gaussian compared to the Halpha filament?"

Response:

Yes. The morphology of the fitted 2D Gaussian was explicitly compared to the Hα filament. This comparison is shown in Table 1, where Models 13 and 8 correspond to the 2D Gaussian and Hα filament spatial templates, respectively. In fact, the Hα template was the first to be tested since it provides the physical motivation.

"Ln 684: Is there a statistical test of the preference for the LogParabola model over PL?"

Response:

Yes. We quantified the preference for a curved spectral model by computing the curvature test statistic (TScurv) using fermipy. For the G189N component we find TScurv close to zero, indicating no statistically significant preference for a LogParabola model over a power-law model (no curvature), whereas for the G189S component we obtain TScurv > 20, indicating a strong preference for a curved spectral shape. We now explicitly describe this methodology and the criterion used to select the best-fit spectral model in the text (lines 986-989 in the revised version).

“Ln 703: Once the spectrum is extracted with the second model, how is this used in calculating systematics? And what ware the values?”

Response:

As stated in lines 698–699 (original version of the manuscript), we consider three distinct sources of systematic uncertainty and combine them in quadrature. For each spectral point, the uncertainty associated with each of the three methods is extracted independently, and the total systematic uncertainty is obtained by adding these contributions in quadrature, following the standard procedure adopted in many previous Fermi-LAT collaboration papers. The resulting systematic uncertainties are reflected in the quoted error bars shown in the corresponding spectral plots. For clarity and conciseness, we do not list the individual systematic contributions from each methodology separately.

“Ln 750: The Alpha measurement is mentioned here, but again this data suggests s249 is much closer”

Response:

Here we simply quote distance estimates for IC443 reported in the literature. As discussed above, the methodologies used for the S249 distance measurement from that specific work is subject to significant uncertainties and is not considered fully reliable. We therefore present these values for completeness while emphasizing the limitations of the underlying methods in the revised manuscript.

“Ln 764: The value os 3x10^4 yrs is repeated several times, but is only derived in one location”

Response:

The value of $3x10^4$ yr is quoted from the literature and originates from a single previous study. The other age estimates discussed in this work are derived independently and yield values of $3.1x10^4$ and $3.2x10^4$ yr, rather than exactly 3×10^4 yr. We have rephrased the opening paragraph of this section (lines 1100-1117 in the revised version) to clearly distinguish between the literature value and the estimates obtained in this work.

“Ln 779: “one derives a local” this is confusing, please consider restructuring the sentence”

Response:

Adopted (lines 1125-1128 in the revised version).

"Ln 801: Why was one set of values chosen to use in the modelling over the other?"

Response:

This was a wording issue. Both sets of values were tested in the modeling as can be seen in lines 889-890 (original version of the manuscript), and the "latter" statement refers to the distance. We have rephrased the text for clarity to avoid any misunderstanding (line 1164 in the revised version). When adopting the two distinct density values, the only resulting change is in the maximum electron energy, which varies from 4 TeV to 7 TeV (as stated in the text, lines 1297-1300 in the revised version) and has a negligible impact on the resulting SED. For this reason, we chose to adopt an intermediate value of 5 TeV and present only a single SED. This choice is now stated more clearly in the revised manuscript (lines 1300-1303 in the revised version).

"Ln 847: ""Nevertheless…" I'm not sure what is sentence is adding other than SED modelling was performed, condor simplifying"

Response:

Rephrased (lines 1208-1212 in the revised version).

"Ln 867: Naima should be \em{naima} I think"

Response:

Adopted (line 1244 in the revised version).

"Ln 888: Were these values fit to the data? Or are hey just a representative set of parameters? Is it possible to provide error on these parameters? Or do the others think with the other assumptions being made this"

Response:

The quoted parameters are representative values (this is now more clearly stated in line 1312 in the revised version) obtained from the naima SED modeling constrained by radio data, X-ray upper limits, and gamma-ray measurements. We are performing a forward modeling/parameter exploration not a statistical fitting with sampling. The parameters are model-derived but not statistically fitted with errors. We preferred to inspect manually a reasonable range of values. Leaving free the fit with so few MWL constraints is often risky, leading to divergence. The reported values are therefore intended to be informative, demonstrating that the observed emission can be reproduced with physically reasonable parameters, rather than representing best-fit values with associated errors. In the absence of more constraining radio and X-ray measurements beyond upper limits, the parameter values can become highly degenerate, making it difficult to define a broader allowable range.

"Ln 892: Again is there a statistical preference of the hadronic vs leptonic model?"

Response:

In the context of naima, the comparison between leptonic and hadronic scenarios is typically not performed through a formal statistical model selection (e.g. likelihood ratio, AIC/BIC). Instead, as is common in the literature, the preference is assessed based on the ability of each scenario to consistently reproduce the multiwavelength data - including radio measurements, X-ray upper limits, and gamma-ray spectra - as well as on physical considerations and the plausibility of the parameter values inferred from the modeling.

"Ln 920: I guess this does not yet indicate this is viable, rather it is intended to show this"

Response:

Adopted (lines 1359-1364 in the revised version).

"Ln 985: I got a bit lost in the text where the 2 Jy value comes from, is this quoted earlier?"

Response:

The 2 Jy value is derived later in the manuscript (for G189N) and is explicitly referred to as such in the text (line 985; original version of the manuscript). It is then computed in lines 1011-1016 (original version of the manuscript). We have

changed the wording to "computed" rather than "reported" to make this clearer (line 1450 in the revised version).

"Ln 987: It's not clear to me why future radio observatories will do better here"

Response:

Current radio constraints for G189.6+3.3 are limited by the remnant's low surface brightness, its large angular extent, source confusion in the crowded IC 443/S249 field, and the likely presence of diffuse thermal free–free emission associated with the S249 HII region, which complicates the isolation of the non-thermal synchrotron component. Future radio observations with improved sensitivity, angular resolution, and multi-frequency coverage - ideally including polarization information - will enable a more reliable separation of thermal and non-thermal emission and improved background characterization. While such observations may not necessarily recover the full extent of the SNR, they are expected to provide significantly stronger constraints on the synchrotron component and its contribution to the total radio continuum emission. We have clarified this point in the manuscript (lines 1451-1455 in the revised version).

"Table 3: I think this is a bit confusing, as far as I can tel some of these values have come from the SED, whereas some (like the B field for the norther component) come from elsewhere."

Response:

In principle, nearly all values reported in Table 3 are either inputs to, or outputs constrained by, the multiwavelength SED modeling. We therefore do not expect the table to be confusing in this respect. The only exception is the magnetic field strength associated with the G189N hadronic component, which is derived separately in Section 4.6 when exploring the proton re-acceleration scenario, rather than directly from the baseline SED modeling. To avoid any ambiguity, we have now explicitly noted this in the table caption.

"Fig 2: I generally think that adding a flat cut-off to the map below a fixed TS value is quite misleading as it can lead to the interpretation of structures in the map as seeming clearer than they really are. This is especially misleading as the TS threshold is changing between the three energy bands"

Response:

As expected, the significance of the detected emission decreases as the lower energy threshold is increased, which naturally leads to different minimum TS values in the maps shown for the three energy bands. The TS cuts are therefore chosen to suppress low-significance background fluctuations in each band and are applied solely for visualization purposes. There is no reason to share a common minimum TS cut. This does not affect any of the quantitative results presented in the paper. This is now explicitly stated in the figure caption to avoid any potential misinterpretation.

"Fig 3: It seems a bit concerning that the PL fit is violating one of the upper limits, this should maybe be commented on. I would imagine this is not statistically significant, but it is not possible to say as no T/S preference is given for log parabola vs PL"

Response:

All data points shown as upper limits correspond to energy bins with TS < 4 and therefore do not represent statistically significant detections. We have verified that the "violation" of the model butterfly by one of the upper limits is not statistically meaningful. One should have in mind that those are conditional limits derived assuming the source is isolated, which is not strictly true here. This behavior is likely due to partial overlap between the G189S and G189N components, such that a weak, sub-significant contribution from G189N may be present at lower energies in G189S spectrum without being formally detected in individual bins. In this low-TS regime, upper limits derived for individual components should be interpreted with caution, and small excursions relative to the global best-fit model are not unexpected. We have added a brief comment in the figure caption to clarify this point.

We thank the reviewers once again for their careful reading of the manuscript and for their continued constructive comments and suggestions, which have further improved the quality and clarity of the paper. Changes to the manuscript are indicated in bold.

Reviewer 1:

I thank the authors for their detailed responses to my original report and for the revisions made to the manuscript. Several of my original concerns have been addressed. I am also satisfied that the most egregious overstatements in the Discussion have been toned down. However, I have significant remaining concerns, in particular about the overall readability of the manuscript.

Spectral index of the Hα filament: The authors' response to my question about the radio spectral index of the filament conflates two distinct issues. My original point was that the challenges described in Section 4.6 regarding flux density measurements of extended sources do not straightforwardly apply to the measurement of a spectral index for a compact, narrow filamentary structure that might be well sampled. The authors argue instead that mismatched angular resolutions and diffuse background contamination make a spectral index measurement unreliable. This is a legitimate practical concern, and one that can be contained within an uncertainty measurement (that's what error bars are for).

I also note that what the authors present as a radio flux density "estimate" for the filament region is in fact a theoretical prediction scaled from the Cygnus Loop using ratios of compressed density and magnetic field strength. The simplest and most convincing approach would be to measure the flux density within a defined aperture in the 1.4 GHz radio map, subtract the background using appropriate control regions, and quote the result with an uncertainty. As far as I remember, the CGPS map (which I think is what Fig. 1, top left is), has zero spacings.

Response:

We agree that the challenges discussed in Sect. 4.6 regarding integrated flux-density estimates for large diffuse remnants do not directly translate to the measurement of a localized compact filamentary structure, although we note that the northern filament itself still represents a relatively extended and morphologically complex region rather than a compact isolated knot. Our original wording did not sufficiently distinguish between these two cases, and we have revised the text accordingly. However, this remains a particularly complex region observationally, and the filament itself is not strikingly bright in absolute terms. It is clearly enhanced relative to the rest of the remnant area (in the remainder of the SNR, the radio emission is generally close to the local background level, making any reliable integrated flux estimate substantially more uncertain), and this is precisely the reason why we restricted our flux-density extraction to the filament/G189N region alone. Perhaps this was not sufficiently clear in the original manuscript, but we have already performed an aperture-based radio flux-density estimate for the filament region using the CGPS 1.42 GHz data. We then compare this observational estimate with the theoretically predicted synchrotron flux density derived from scaling the crushed-cloud re-acceleration scenario of the Cygnus Loop. The purpose of this comparison is precisely to assess whether the observed radio

enhancement in the filament is broadly consistent with expectations from the crushed-cloud scenario. We have revised the manuscript to make this observational-versus-theoretical comparison significantly clearer.

We further clarify that our decision not to derive a radio spectral index is not because such a measurement is formally impossible. Indeed, a spectral index can in principle be derived from two flux-density measurements. Of course, additional flux-density measurements at multiple frequencies would clearly improve the robustness of any spectral-index determination. However, here the filament is only marginally visible in two available radio surveys at sufficiently separated frequencies, already making the inferred slope highly sensitive to systematic effects. In the present case the main issue is that the two available measurements are both highly uncertain and heterogeneous, such that any derived slope would be dominated by systematic rather than statistical uncertainties.

In particular, the aperture-based CGPS 1.42 GHz estimate alone spans a factor of more than three (2.0--6.3 Jy) depending on the adopted background treatment and assumptions regarding thermal contamination from S249. This already demonstrates that the uncertainty budget is dominated by background-selection systematics rather than measurement noise. Any corresponding higher-frequency estimate from the GB6 survey is expected to carry even larger uncertainties on the spectral index estimate because of differences in angular resolution, spatial filtering, sensitivity to diffuse emission, and local background structure.

Under these conditions, a formal spectral-index value would not constitute a meaningful physical diagnostic. We also note that particle re-acceleration scenarios in radiative shocks do not necessarily predict a radically different radio spectral slope compared to standard DSA models. Although re-acceleration/compression scenarios may in some cases produce somewhat steeper radio spectra, differences at the level of $\Delta\alpha \sim 0.1$–0.2 that would be needed for a discrimination cannot be robustly constrained with the current observational uncertainties and systematic effects. Consequently, the radio spectral index cannot presently be used as a reliable discriminating diagnostic between standard DSA and re-acceleration scenarios in G189N. Additional matched-resolution radio measurements with improved sensitivity and better-controlled systematics would therefore be required to derive a physically meaningful spectral index for the filament.

Section 4.6 has been revised and restructured to make this discussion more explicit.

The binary supernova claim. My more serious concern relates to the central claim of the paper, namely that G189.6+3.3 and IC 443 constitute the first observed binary supernova system. While I acknowledge that the authors have made genuine efforts to strengthen this argument I still do not find the overall case convincing. For the probability calculation, both estimates rest on a surface density of Galactic SNRs derived from the Green catalogue, which contains 310 entries, which we all understand to be a severe underestimate of the true SNR population. More importantly, SNRs are not uniformly distributed across the Galactic plane: they cluster in star-forming regions and along spiral arms, and the IC 443 complex lies within the Gem OB1 association, a region of recent massive star formation where the local surface density of SNRs is substantially higher than the all-sky average.

Beyond the probability calculation, the broader binary argument rests on a chain of evidence in which each link carries substantial uncertainty. The distance estimates for the two remnants are

compatible within their error bars, but those error bars are large. The binary population synthesis demonstrates that the proposed scenario is physically possible, but given the breadth of the allowed parameter space, this is not a strong discriminating result. The jet-like features and ejecta abundances are suggestive but explicitly acknowledged by the authors themselves to be non-unique signatures of a binary origin. The most compelling evidence for a physical association between the two remnants remains, as I noted in my original report, the fact that both appear to interact with the same HII region, S249. However, the gap between "both remnants interact with the same dense structure" and "these remnants originate from a binary progenitor system" is a large one, and the paper does not bridge it convincingly.

However, that is my own interpretation of the evidence that the authors have provided (although I do note that the probability of chance alignment calculated is an underestimate, as it does not accurately take into account the specific Galactic location of the sources), and I understand that part of the point of a paper is that the authors have a point that they want to make and some evidence that supports it. Which brings me to my next comment.

Response:

We agree that the case for a binary-supernova origin is necessarily based on a combination of several observational indications rather than on a single definitive signature, and we acknowledge that each component of the argument carries its own uncertainties and limitations. In accordance with the comments from Reviewer 2, we have carefully revisited and revised (when needed) statements related to the binary interpretation throughout the manuscript to ensure that we are not overstating the current level of evidence or presenting the scenario as a secure or unambiguous conclusion.

As we already explicitly state in the revised manuscript, there is currently no single observational signature that can uniquely confirm such a binary-supernova system. Rather, our interpretation is motivated by the cumulative combination of several independent observational and theoretical indications which, when considered together, form a strong and compelling case for a physical association between G189.6+3.3 and IC 443 within a binary-supernova framework. We believe that our work demonstrates that G189.6+3.3 and IC 443 presently represent the first and only particularly compelling candidate system for such a scenario and therefore warrant detailed investigation and discussion. We also emphasize that we have gone in depth to examine whether any currently available observational evidence or theoretical prediction contradicts such a scenario, and we do not find this to be the case.

We agree with the referee that the probability estimates should be interpreted with caution for two main reasons. First, the Green catalogue is incomplete, and the 310 catalogued Galactic SNRs should be regarded as a lower limit to the true Galactic SNR population. Increasing the assumed total number of SNRs would correspondingly increase the expected surface density and hence the chance-alignment probability. Second, although our calculation is already more conservative than an all-sky estimate because we distribute the catalogue population only over the Galactic plane region |b|<5 deg, it still assumes a mean surface density within this region and therefore does not explicitly account for smaller-scale clustering within the plane. This could be particularly relevant for the Gem OB1/S249 environment, where the local density of massive-star remnants may be higher than the Galactic-plane average.

To address these concerns, we have substantially revised and expanded the probability discussion in the manuscript. In particular, we now explicitly distinguish between: (i) the centroid-based coincidence probability, (ii) a conservative shell-overlap probability, and (iii) a stricter containment-like overlap configuration motivated by the observed morphology and recent eROSITA spectral analysis. We clarify that the previously quoted overlap-based probability of ~0.9% is intentionally conservative because it only requires any shell intersection, whereas the observed geometry suggests a substantially stronger overlap. Adopting a stricter containment-like configuration reduces the corresponding probability to the level of ~0.1%, comparable to the centroid-based estimate.

We have also added an explicit discussion of how SNR catalogue expected incompleteness and possible local surface-density enhancements in Gem OB1 could inflate these probabilities. In particular, we now discuss the expected Galactic SNR population (~1000-1200 remnants) and the extent to which the adopted |b|<5 deg restriction already partially accounts for Galactic-plane clustering. We further show that using the broader Gem OB1 molecular-cloud complex itself does not imply a substantially larger local SNR surface density than the Galactic-plane average adopted in our baseline estimate. Overall, while these effects can increase the chance-alignment probabilities, the revised calculations still indicate that a purely coincidental spatial and distance association between G189.6+3.3 and IC 443 remains highly unlikely. We believe that these additional calculations further strengthen our overall argument by demonstrating that, even under intentionally conservative and likely over-penalizing assumptions, the probability of a purely coincidental spatial and distance association between G189.6+3.3 and IC 443 remains sufficiently low (≲1%).

These low probability estimates for a chance spatial and distance coincidence, together with the broader observational evidence presented in this work — including the compatible distance constraints, the common interaction of both remnants with the S249 environment, the large projected overlap between the remnants, the eROSITA spectral indications that the X-ray emitting structures may completely overlap, the jet-like features and ejecta asymmetries discussed in the manuscript, and the binary-population synthesis results demonstrating that such a configuration is physically plausible — collectively provide strong support for the interpretation of a physically associated binary-supernova system. This interpretation is further motivated by the fact that the majority of massive stars are now known to form and evolve within binary or higher-order multiple systems.

General remarks on the manuscript: I am concerned about the overall readability and coherence of the revised manuscript. The authors have responded to each referee comment individually, but the paper does not read as a coherent whole. Some basic examples: there are frequent forward references that are not always necessitated by the structure of the paper (e.g., Section 4.5 references Section 4.6) and the opening of Section 4.5 does not even explicitly state which of the two SNRs that the paper discusses is being modeled. Also, although I appreciate that the authors have stepped up their figures game, for a paper whose central claims rest so heavily on morphological and spatial coincidences, it is surprising how little use is made of the figures to guide the reader through the argument.

I would also recommend some serious editing. If I had not refereed this paper, I would not have made it past the introduction simply because of the quality of the writing. The title itself is a harbinger of what's to come. Between lines 1153 and 1160, four consecutive sentences start with: "Consequently, …," "Importantly, …," "Consequently, …," "Subsequently, …" I would urge the authors to try to read the text as a new reader would, and ask themselves whether they can follow the argument.

The paper attempts to establish many results simultaneously, and the through-line of the argument is frequently lost. I would strongly encourage the authors to undertake a thorough structural revision of the manuscript, with particular attention to ensuring that each claim is clearly stated, the supporting evidence is presented in a logical sequence, and the distinction between what is established and what is speculative is made explicit throughout.

Response:

We agree that, in the previous revision, the manuscript became overly fragmented at places as a consequence of addressing a large number of detailed referee comments individually, which at times affected the overall narrative flow and coherence of the paper.

In response, we have now performed a substantial editorial and structural revision of the manuscript aimed specifically at improving readability, logical progression, and clarity of the main arguments. In particular, we have revised multiple sections to improve transitions between subsections, and ensure that the object or physical scenario under discussion is explicitly identified at the beginning of each section. We have also carefully revised repetitive sentence constructions and significantly edited the text for readability, style, and flow throughout the manuscript.

We also agree that, given the importance of the morphological and spatial associations to the interpretation presented in this work, the figures should play a more central role in guiding the reader through the argument. We have therefore revised and expanded the figure presentation and corresponding discussion in the text to better highlight the relevant spatial correlations and morphological features supporting the proposed interpretation.

More broadly, we have carefully revisited the structure of the manuscript with the referee's comments in mind, with particular attention to clearly separating observational results, theoretical interpretation, and more speculative aspects of the discussion.

Overall, we believe that these revisions have substantially improved both the readability and the coherence of the manuscript.

In more detail:

a)We agree that the opening of Section 4.5 was not sufficiently explicit regarding which remnant is being modeled, particularly given that the manuscript discusses both G189.6+3.3 and IC 443 throughout the paper. Since the large majority (if not all) of the analysis in the manuscript focuses on G189.6+3.3, we had originally considered the context to be sufficiently clear. However, the referee is correct that this should

nevertheless be stated explicitly at the beginning of the section to avoid any ambiguity for the reader. We have therefore revised the opening of Section~4.5 to clearly state that the multiwavelength SED modeling refers specifically to G189.6+3.3 and its associated gamma-ray emission.

b)We have also searched for and evaluated the need of forward references. We note that the manuscript follows the standard structure commonly adopted in Nature-format papers, where the main results and physical interpretation/discussion are presented prior to the detailed methodological discussion (methods). As a result, a certain number of forward references are intentionally retained where the results or discussion section naturally points the reader toward the subsequent methodological or modeling details required to support the interpretation. In general, we believe that the forward references currently present in the revised version are well placed and do not interrupt the flow.
The problem is not the references themselves — it is mainly that there are too many "refer to section X" interruptions inside already very dense paragraphs. So, we have softened this by alternating the language.
To address the specific example raised by the referee regarding Section 4.5, both references serve a purpose:

1. Radio flux estimate → Section 4.6
   This is okay because Section 4.5 uses the 2 Jy value for the broad MWL SED modeling, while Section 4.6 gives the detailed radio extraction and re-acceleration interpretation.
2. Alternative acceleration mechanisms → Section 4.6
   This is also okay because Section 4.5 first shows that standard DSA can fit the northern gamma-ray spectrum phenomenologically, but Section 4.6 then explains why proton re-acceleration is preferred in the dense radiative shock.

We just made the references sound intentional, not like "go read later." And we retain these forward references intentionally because they reflect the logical structure of the analysis. We first test whether the gamma-ray emission from G189.6+3.3 can be described within a standard DSA-based multiwavelength SED framework. This provides a baseline phenomenological model for the northern and southern components. We then turn, in the following section, to the more specific proton re-acceleration/crushed-cloud scenario, which is physically motivated by the dense radiative-shock environment of G189N. The flux estimates from the modeling scaling and the aperture photometry is therefore discussed there, where it directly tests whether the observed radio emission is consistent with the predicted synchrotron emission from the re-acceleration model.

c) The referee is correct that a number of consecutive sentences in the referred section began with similar transition words (e.g. "Consequently", "Importantly", "Subsequently"), which interrupted the narrative flow. We have carefully revised these passages throughout the manuscript to improve readability, vary sentence structure and transitions, and make the argumentation easier to follow for a first-time reader, while preserving the scientific content and logical progression of the discussion.

Section-by-section:

Introduction section:

The Introduction section has been revised to improve the overall readability, connectivity, and logical progression of the manuscript. In particular, the paragraph structure was reorganized to avoid mixing different topics within the same paragraph and to ensure a clearer transition between the broader SNR context, the observational history of G189.6+3.3, the recent multiwavelength developments, and the scientific motivation of this work. The basic system geometry is introduced early in the Introduction through the relevant figure, allowing the reader to immediately identify the relative locations of G189.6+3.3, IC 443, and the S249 HII region before the detailed discussion of their possible physical connection.

Results section:

We edited the section to ensure that each major paragraph follows a clearer logical progression, beginning with the relevant observational evidence, followed by its physical interpretation, and finally its broader implication. We also revised the transitions between paragraphs to strengthen the continuity of the argument and to better connect the different aspects of the analysis.

More specifically, the revised Results section now follows a more coherent progression from: (i) the detection and morphological characterization of the extended gamma-ray emission, to (ii) the spatial decomposition into the G189N and G189S components, (iii) the spectral characterization of the two regions, (iv) the distinction between the hadronic- and leptonic-dominated components, (v) the evidence supporting proton re-acceleration in the northern interaction region, and finally (vi) the implications for the binary-SNR interpretation and the broader astrophysical context. We additionally strengthened the integration of the figures within the text by explicitly guiding the reader to the relevant morphological and spectral features supporting each conclusion.

Discussion section:

The key question here is whether a *new reader* can now follow the argument without getting lost. We think the answer is "mostly yes," but there are still a few overly dense passages where the flow of the argument could be improved further, and we therefore revised these parts to soften the presentation. The Discussion is already explicitly subdivided into:

- *Nature of the gamma-ray emission*
- *Evidence for proton re-acceleration*
- *A binary supernova scenario*
- *Implications at very high energies*

There are still refinements that can be made to further improve readability. To address this, we split several overly long paragraphs to improve readability and logical flow, and added additional figure references to guide the reader more clearly through the observational arguments and spatial correlations discussed in the text. We also reordered several paragraphs and reduced repetitions to improve the overall readability and logical flow of the discussion.

We note that the "binary supernova scenario" discussion is perhaps the densest part of the manuscript, as it combines an intro, population-synthesis modelling, and probability arguments. To improve readability and better guide the reader through these distinct lines of reasoning, we further separated this discussion into two clearer thematic components.
In addition, we identified that part of the readability issue in the "binary supernova scenario" discussion arose because the population-synthesis interpretation was interrupted by a separate discussion on progenitor properties before returning to the COMPAS results. To improve continuity and guide the reader more clearly through the argument, we reordered these paragraphs such that the COMPAS and population-synthesis discussion now remains self-contained before transitioning to the independent progenitor-mass and ejecta-based evidence supporting the binary scenario.
In the second thematic component of the "binary supernova scenario" discussion, which focuses on the probability of a chance association, we also substantially revised the presentation to improve readability and logical flow. In particular, we reduced repetition between the different probability estimates, clarified the distinction between the centroid-based, overlap-based, and stricter containment-like configurations, and reorganized the discussion of catalogue incompleteness and local clustering effects into a clearer sequence separating (i) the potential inflation factors and (ii) the mitigating considerations already partially accounted for in the baseline estimates. We additionally introduced clearer transitions between the baseline probability estimates and the subsequent conservative sensitivity tests.

A small interruption in the flow caused by repeated discussion of the limited angular resolution of current TeV instruments has also been corrected.

We believe that the primary readability issues were concentrated in the Discussion section and the LAT data section in methods (see below). The former section is inherently dense due to the combination of observational, theoretical, and statistical arguments presented there. We have therefore made substantial efforts to improve its clarity and logical flow, including restructuring several parts of the discussion, reducing repetition, and introducing clearer thematic transitions to better guide the reader through the different lines of reasoning.

Methods section:

Extensive restructuring has also been carried out in the LATdata analysis section to improve the logical progression of the methodology and results. In particular, the discussion of source detection, evidence for a separate gamma-ray source, morphological modeling, residual analysis, and validation of the extended-source interpretation has been reorganized to avoid back-and-forth transitions and to provide a clearer and more continuous narrative flow.
We also significantly revised the multiwavelength SED modeling section to improve the transition between the observational constraints and the physical interpretation of the G189N and G189S components. Finally, the proton re-acceleration section was carefully restructured to clarify the connection between the radiative-shock scenario, the radio

synchrotron expectations, and the comparison between the predicted and observed radio flux densities.

We would like to emphasize that these revisions did not alter any of the results o or scientific interpretation or overall information presented in the manuscript. The scientific content and conclusions remain unchanged; the text has instead been more carefully organized, with improved paragraph structuring, smoother transitions between concepts, and a reduction of repetitive interruptions in order to enhance the overall readability and flow of the paper. The large majority of the bold text throughout the revised manuscript corresponds to passages that were reorganized, restructured, or reworded to improve the flow and readability of the paper. In addition, we also highlighted in bold the opening words of several newly introduced paragraphs in order to make the structural revisions more easily identifiable to the referee.

Reviewer 2:

I thank the authors for their substantial revision. The manuscript addresses several of the concerns raised in the previous version. These additions have improved the manuscript and made the interpretation more complete. In its revised form, the paper presents noteworthy results that are clearly relevant to the field, and the analysis is now more convincing and better contextualized within the broader literature. The study compares well with the established literature and, in my view, contains sufficient scientific value to make it a potentially publishable contribution.

That said, I remain unconvinced that the most far-reaching claims are demonstrated at the level currently implied by the title, abstract, and several statements in the main text. In particular, the binary-supernova interpretation is now better motivated, and the newly added chance-alignment estimates are useful additions that directly address an important concern from the previous report. However, I still find that this interpretation is presented somewhat too strongly relative to the available evidence. The probability estimates are informative, but they are approximate and do not constitute formal model comparisons. As such, the current results support plausibility, but not yet a unique inference.

I therefore recommend that the authors moderate the wording throughout, especially when expressions such as “first known example” or “strong evidence” are used. At present, the manuscript makes a compelling case for a very interesting candidate system, but not yet, in my view, for a secure first detection.

Response:

Following the reviewer’s broader concern regarding the strength of the binary-supernova interpretation, we moderated the wording throughout the manuscript where appropriate. Expressions implying definitive confirmation were revised to more carefully reflect that the current results provide strong support, but do not yet conclusively prove, the binary-supernova scenario. However, we note that phrases such as “the first known example” and “first detection” were retained only in appropriately qualified form, namely “what may represent the first known example” and “compelling evidence for the first detection,”. This wording was intentionally adopted to emphasize that the proposed

binary-supernova interpretation remains a strongly supported candidate scenario rather than a definitively confirmed system. In addition, the very next sentence explicitly states that no single observational signature can unambiguously establish a binary progenitor origin in evolved SNRs, and that the interpretation instead relies on the convergence of multiple independent lines of evidence. We would also argue that the use of expressions such as "strong evidence" remains appropriate for the level of observational and theoretical support presented in this work. Our intention in the revised manuscript was therefore not to weaken the significance of the results, but rather to ensure that the wording does not imply a fully secured or definitively confirmed binary-supernova scenario.

In particular, the title has been revised, also following recommendations from Reviewer 3.

As far as the abstract is concerned, there is a single statement currently present: "This positions G189.6+3.3 at the same distance as IC 443, with a linear separation between the two remnants and a time delay in their explosion events, bolstering the hypothesis that these two remnants are part of a binary system, both resulting from separate supernova events." We believe that this wording already appropriately reflects the current level of evidence, as it explicitly refers to a hypothesis being strengthened rather than a definitively confirmed scenario.

We do not list every modified statement individually here; however, where deemed appropriate, softened or revised wording is highlighted in bold throughout the revised manuscript. For clarity, all such statements, whether modified or retained, are highlighted in bold so that they can be easily identified by the reviewer.

Reviewer 3:

This version of the manuscript has received major improvements and extensive addition of text for clarification. I can see most of the raised concerns have been addressed and I can now recommend it for publication without problems. The title is still sounding a little awkward. I would suggest dropping the parentheses and even better the colon as well. I also cannot fully understand the meaning of "duel" here as there is no competition involved as far as I understand. I guess the authors are intending to mean "co-existence" instead? Other than that I do not have any further comment.

Response:

Regarding the title, we agree that the previous formulation was overly complex and that the term "duel" could imply direct competition rather than coexistence of the hadronic and leptonic gamma-ray components. Following the reviewer's suggestion, we have substantially revised the title to improve clarity, readability, and scientific precision. In particular, we removed the parentheses and colon, avoided the use of "duel," and adopted wording that more accurately reflects the spatial segregation and environmental connection of the gamma-ray components within the system. In addition, following the broader concerns raised by Reviewers 1 and 2 regarding the strength of the binary-

supernova interpretation — which is strongly supported by the current evidence, although not yet 100% secured — we softened the wording throughout the manuscript, including the title. The new title is:

"Spatially segregated hadronic and leptonic gamma rays unveil a shared-cloud interaction linking IC 443 and G189.6+3.3 in the first candidate binary-supernova system"

Reviewer 4:

The authors have made significant changes to the manuscript to address the comments from all referees and I would like to thank them for their very comprehensive response to referee comments. Generally speaking I think the authors have addressed well most of the comments I made in my report.

The most significant question I had was the relative distance of s249 and IC 443 and some measurements which suggested the two were not located at the same distance. The authors addressed these concerns in both their responses to comments and within the paper itself, describing the difficulties in interpreting velocity measurements as a distance in this region. They also provide explain the evidence for the co-location of these objects through signs of interaction between the two.

Most suggestions regarding wording were adopted or addressed in some way.

I still think the issue of having a hard cut in the TS maps could introduce some apparent structures in the maps which are not truly present, the choice of this cut-off value could change the apparent size of the sources to the reader. Especially seen as the source significance is quite high I don't think the maps would look that different without it. However, as the authors explain this thresholding an it is not important for the results of this paper and simply to make the plot easier to read, I would simply ask that the authors consider changing this rather than insisting.

Response:

We agree that applying a hard threshold in the TS maps can visually affect the apparent extent of the emission depending on the chosen cut-off value. However, as correctly noted by the reviewer, the thresholding was introduced solely as a visualization aid to improve the readability of the maps and does not affect any quantitative analysis or conclusions presented in this work. We also made sure to adopt, for each map, a cut-off value that remains representative of the actual extension of the emission. Since this is already explicitly stated in the figure caption (following the comment of the reviewer in the previous round), and because the underlying morphology and significance of the source remain unchanged, we have retained the current representation of the TS maps. As an example, we attach here the corresponding >1 GeV TS map without applying a hard cut, showing that the overall morphology and extension of the emission remain consistent with the maps presented in the manuscript. Since the adopted thresholds are

relatively low (TS ~7, 5.5, and 4.5 for the >1 GeV, >5 GeV, and >10 GeV maps, respectively, corresponding to significance levels of approximately <2.5σ), the additional low-level emission visible without thresholding is likely dominated by statistical fluctuations rather than significant extended structure. We have also made the figure caption more specific to clarify the motivation and choice of the applied TS thresholds.

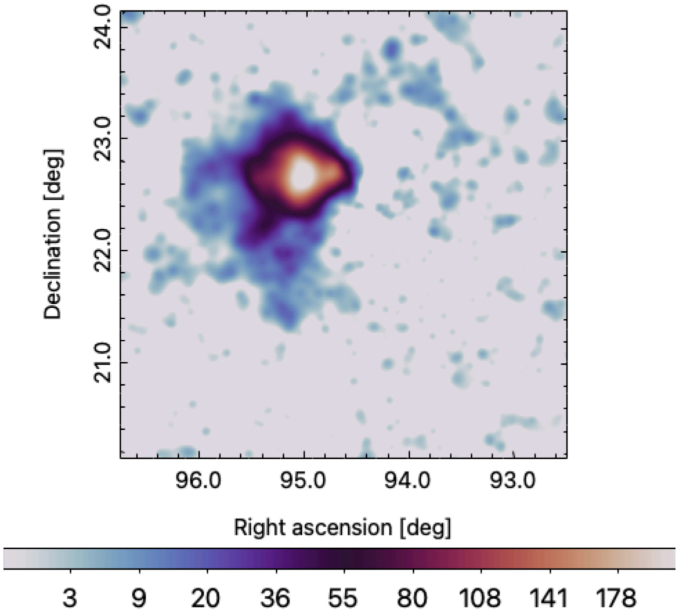

I accept the authors statement on the IC vs hadronic model comparison, as for example it may be possible to fit the northern spectrum with an IC model with totally implausible parameters which might also give a reasonable fit statistic, even though being completely unphysical. However, this is important to make note of. Currently (as far as I can see) the only mention of preference is that is does not show an IC peak, this perhaps would be worth a little more explanation as to why there is no plausible IC model that can fit this region.

Response:

We agree that a comparison between models should be accompanied by a discussion of why an IC scenario is disfavored, since formally acceptable IC fits could in principle be obtained using unrealistic or physically implausible parameter combinations.
While qualitative arguments based on the spectral shape and morphology were already included in the Results and Discussion sections, we believe that the most appropriate place for a more detailed discussion is within the dedicated multiwavelength SED modeling section, where the relevant physical parameters and environmental constraints are quantitatively evaluated. We therefore expanded the discussion at the end of the G189N modeling section, specifically in the paragraph where we already explain why hadronic emission is expected to dominate over both inverse-Compton and Bremsstrahlung scenarios in the dense S249 interaction region (last paragraph of section 4.5). In this context, we now explicitly clarify why a leptonic IC interpretation is

disfavored beyond the simple absence of a characteristic IC peak in the SED (as mentioned in the Results and Discussion sections). In particular, we now emphasize that reproducing the observed gamma-ray emission through IC scattering would require electron populations and environmental conditions that are not physically plausible for this region, especially considering the strong spatial correlation between the gamma-ray emission, the molecular material, and the prominent Hα filament associated with the S249 cloud interaction. The combined spectral and morphological evidence therefore strongly favors a hadronic origin for the northern component.
